# Distribution Pattern and Climate Preferences of the Representatives of the Cosmopolitan Genus *Sirthenea* Spinola, 1840 (Heteroptera: Reduviidae: Peiratinae)

**DOI:** 10.1371/journal.pone.0140801

**Published:** 2015-10-23

**Authors:** Dominik Chłond, Agnieszka Bugaj-Nawrocka

**Affiliations:** Department of Zoology, Faculty of Biology and Environmental Protection, University of Silesia, Katowice, Poland; University of Waikato (National Institute of Water and Atmospheric Research), NEW ZEALAND

## Abstract

The main goal of this study was to predict, through the use of GIS tool as ecological niche modelling, potentially suitable ecological niche and defining the conditions of such niche for the representatives of the cosmopolitan genus *Sirthenea*. Among all known genera of the subfamily Peiratinae, only *Sirthenea* occurs on almost all continents and zoogeographical regions. Our research was based on 521 unique occurrence localities and a set of environmental variables covering the whole world. Based on occurrence localities, as well as climatic variables, digital elevation model, terrestrial ecoregions and biomes, information about the ecological preferences is given. Potentially useful ecological niches were modelled using Maxent software, which allowed for the creation of a map of the potential distribution and for determining climatic preferences. An analysis of climatic preferences suggested that the representatives of the genus were linked mainly to the tropical and temperate climates. An analysis of ecoregions also showed that they preferred areas with tree vegetation like tropical and subtropical moist broadleaf forests biomes as well as temperate broadleaf and mixed forest biomes. Therefore, on the basis of the museum data on the species occurrence and ecological niche modelling method, we provided new and valuable information on the potentially suitable habitat and the possible range of distribution of the genus *Sirthenea* along with its climatic preferences.

## Introduction

Peiratinae (Hemiptera: Heteroptera: Reduviidae) is one of the medium-sized subfamily of assassin bugs (Reduviidae), with 30 known genera [1–10] consisting of various sized predators with almost unknown biology.

**Fig 1:**
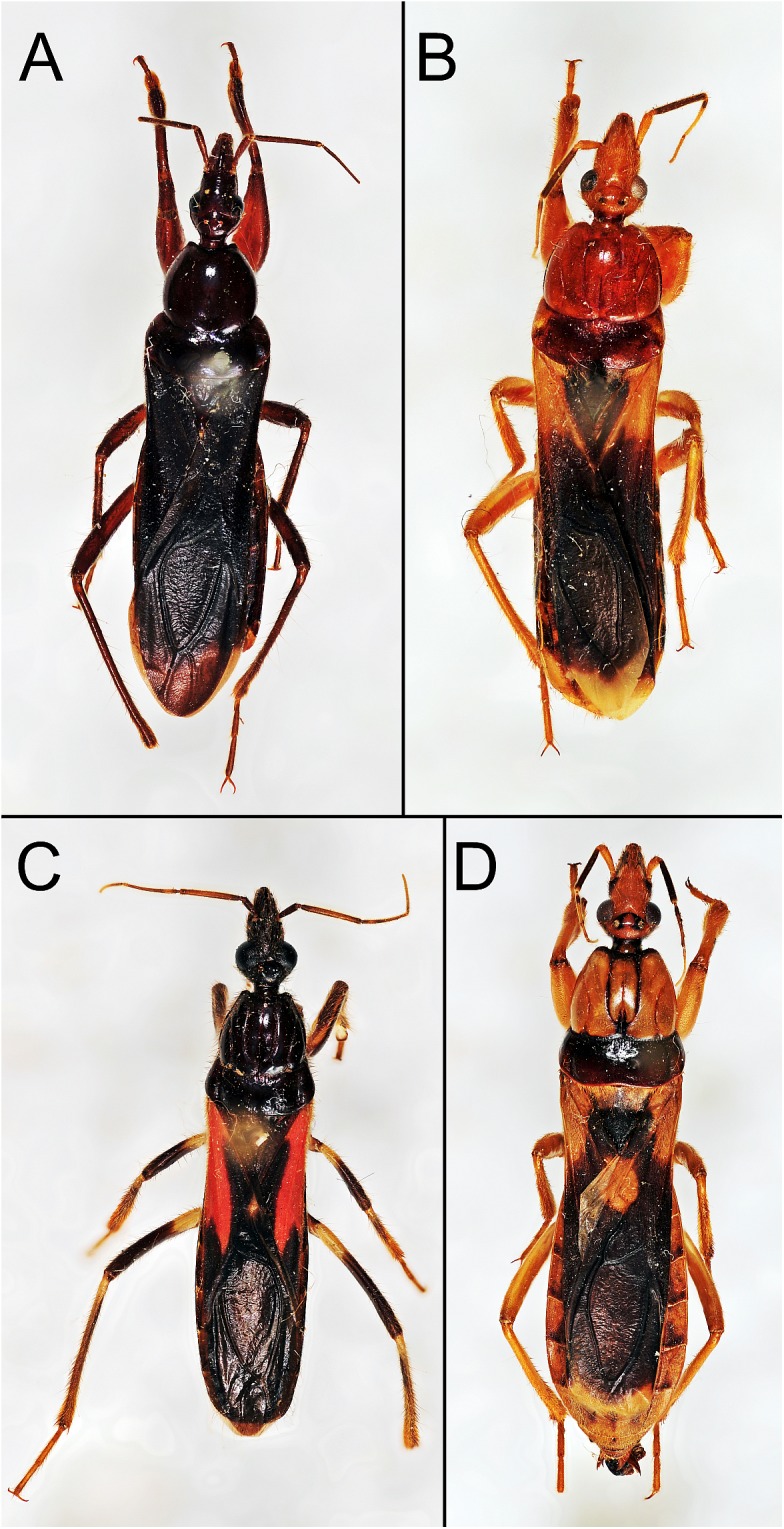
Dorsal habitus of the selected representatives of the genus *Sirthenea*. (A) *S*. *atrocyanea* from Madagascar. (B) *S*. *rapax* from Africa. (C) *S*. *stria* from Americas. (D) *S*. *flavipes* from Asia.

Genus *Sirthenea* was established by Spinola [11] as a monotypic genus based on *Reduvius carinatus* Fabricius, 1798 [12] (placed also in genus *Rasahus* by Amyot and Serville [13]), and it has only one known subgenus *Monogmus* described by Horváth [14] and distributed exclusively on Madagascar. *Sirthenea* can be easily recognized among other genera of Peiratinae by the distinctly elongated body and anteocular part of the head (which is triangular in dorsal view), as well as shortened and rounded femora (Fig 1). However, the most important taxonomic character differentiating *Sirthenea* from other Peiratinae is the absence of fossa spongiosa on the medial tibiae (except present in the Australian species *S*. *laevicollis* Horváth, 1909), which is the character used by Spinola [11] to diagnose this genus.

The distribution of Peiratinae is limited to select zoogeographical regions, e.g., primarily confined to tropical areas (S1 Appendix). We can clearly distinguish groups of genera distributed in Nearctic and Neotropical regions: *Eidmannia* Taeuber, 1934 [15]; *Froeschnerisca* Coscarón, 1997 [5]; *Lentireduvius* Cai & Taylor, 2006 [9]; *Melanolestes* Stål, 1866 [16]; *Phorastes* Kirkaldy, 1903 [17]; *Rasahus* Amyot & Serville, 1843 [13]; *Thymbreus* Stål, 1859 [18]; *Tydides* Stål, 1865 [19]; *Zeikiria* Gil-Santana & Costa, 2003 [8]; Ethiopian region: *Fusius* Stål, 1862 [20]; *Lamotteus* Villiers, 1948 [21]; *Microcleptocoris* Villiers, 1968 [22]; *Neopirates* Miller, 1952 [23]; *Pachysandalus* Jeannel, 1916 [24]; *Parapirates* Villiers, 1959 [25]; *Pteromalestes* Miller, 1959 [26]; *Rapites* Villiers, 1948 [21]; Madagascan region: *Bekilya* Villiers, 1949 [27]; *Hovacoris* Villiers, 1964 [28]; *Pseudolestomerus* Villiers, 1964 [28]; Oriental region: *Calistocoris* Reuter, 1881 [29]; *Catamiarus* Amyot & Serville, 1843 [13]; *Ceratopirates* Schouteden, 1933 [30]; Australian region: *Brachysandalus* Stål, 1866 [16]; and genera distributed in Ethiopian and Oriental regions: *Androclus* Stål, 1863 [31]; Ethiopian, Madagascan and Oriental regions: *Lestomerus* Amyot & Serville, 1843 [13]; Ethiopian, Madagascan, Palaearctic and Oriental regions: *Peirates* Serville, 1831 [32], *Phalantus* Stål, 1863 [31]; as well as Ethiopian, Madagascan, Palaearctic, Oriental and Australian regions: *Ectomocoris* Mayr, 1865 [33] (S1 Appendix). Among all known genera of the subfamily Peiratinae, only *Sirthenea* occurs on almost all continents and zoogeographical regions (S1 Appendix) and is, thus, an exception within the Peiratinae.

Throughout the years, the number of newly described species in *Sirthenea* has grown, with 40 described species [34–36,1,37–41]. However, the representatives of this genus distributed outside of the Americas are currently under revision, and the number of described species will change. The increasing number of newly described species has not changed our knowledge about the biology, as well as distribution, of the genus [42,34]. Representatives of *Sirthenea* are probably ground-dwelling, non-specialized predators, preying on other insects at night on the ground and in very different microhabitats. Due to the very broad spectrum of environments in which individuals of each species are collected, one of the more interesting issues becomes the knowledge about the environmental niches preferences of *Sirthenea*.

With the ability new computer technology, in particular niche modelling, species occurrence data are now more valuable. Current studies of species and higher taxa now will be enriched on environmental aspect. This is important for taxa whose habitat preferences have not yet been fully understood, e.g., in terms of climatic conditions.

The aim of this paper is to present the distribution of all species of *Sirthenea* described so far, with comparison of the other known Peiratinae genera, to understand their climatic preferences and potential distributional range. For this purpose, ecological niche modelling is used, which is based on the maximum entropy model (Maxent)–a machine learning method [43]. This method is widely used in faunal and floral studies that investigate, e.g., biogeography, ecology or evolutionary biology [44–47]. Furthermore, analysis of distributions with respect to the terrestrial ecoregions allows us to show a correlation between species of *Sirthenea* and major global plant communities.

## Material and Methods

### Occurrence data

Places of occurrence come from the determination labels of specimens derived from the following museums: The Natural History Museum, London, United Kingdom (20 specimens); China Agricultural University, Beijing, China (1 specimens); Hungarian Natural History Museum, Budapest, Hungary (8 specimens); Moravian Museum, Brno, Czech Republic (10 specimens); Muséum National d'Histoire Naturelle, Paris, France (30 specimens); Musée Royal de l'Afrique Centrale, Tervuren, Belgium (12 specimens); Naturhistoriska riksmuseet, Stockholm, Sweden (3 specimens); Naturkundesmuseum, Erfurt, Germany (4 specimens); National Museum (Natural History), Prague, Czech Republic (18 specimens); Naturhistorisches Museum Wien, Wien, Austria (16 specimens); Online Zoological Collections of Australian Museums; http://ozcam.org.au/ (1 specimens); Rijksmuseum van Natuurlijke Historie, Leiden, Netherlands (27 specimens); Forschungsinstitut und Naturmuseum Senckenberg, Frankfurt-am-Main, Germany (1 specimens); Tiroler Landesmuseum Ferdinandeum, Innsbruck, Austria (3 specimens); United States National Museum, Washington D.C., USA (45 specimens); Universiteit van Amsterdam, Instituut voor Taxonomische Zoologie, Zoologisch Museum, Amsterdam, Netherlands (1 specimens); Museum für Naturkunde der Humboldt-Universität, Berlin, Germany (5 specimens); University of Copenhagen, Zoological Museum, Copenhagen, Denmark (1 specimens) and Zdenek Jindra private collection, Prague, Czech Republic (12 specimens).

A total of 521 unique occurrence localities were compiled for the representatives of the genus *Sirthenea* in Africa and Madagascar (61), North America (61), Central America (23), South America (206) (altogether 290 occurrence localities were used to the model for the Americas), as well as Asia, Oceania and Australia (170). All these points were used for the global model. All occurrence data were based on examination of specimens studied in the museum collections (see abbreviations for depositories) and obtained from scientific literature [1,34–41,48,49]. Records with unspecified or unknown localities were not used. All localities were georeferenced using Google Earth 7.1.2.2041 [50] (geographical projection, decimal degrees, datum: WGS84). For details of all occurrence localities used in the MaxEnt model, refer to Table A in S2 Appendix.

### Environmental predictors and climate classification

Environmental variables were used as potential predictors of habitat distribution for the representatives of *Sirthenea*. The study was based on 19 bioclimatic variables derived from the WorldClim 1.4 dataset ([51]; http://www.worldclim.org). A spatial resolution of 30 arc-seconds (~1 km^2^) for continental models and 2.5 arc-minutes (~5 km^2^) for global model was chosen. All of the maps were prepared in GRASS GIS 6.4.3 ([52]; http://grass.osgeo.org) and SAGA GIS 2.1.0 ([53]; http://www.saga-gis.org). Climatic preferences were defined using the Köppen-Geiger climate classification system [54].

### Ecological niche modelling

All models of the potential distribution of *Sirthenea* representatives were made using the MaxEnt software (version 3.3.3k; http://www.cs.princeton.edu/~schapire/maxent), based on a maximum entropy algorithm [43]. The logistic output of MaxEnt was used with prediction values from 0 (unsuitable habitat) to 1 (optimal habitat) and the following settings were chosen: regularization multiplier = 1, maximum number of iterations = 5000, maximum number of background points = 10.000 and a convergence threshold = 0.00001. Such settings allow for model convergence in adequate time [43,55,56]. A 50-fold cross-validation was ran using the full occurrence dataset randomly split into a 75% training and 25% test dataset [43,57]. A jackknife test was used in order to show the relative importance of each predictor variable. To assess the quality of the model, a receiver operating characteristic (ROC) analysis was also used [58], and the area under the ROC curve (AUC) was measured. AUC was calculated to show the performance of the model and the weight of the omission error and commission error equally. It is worth noting that recent studies indicate that the AUC values depend on the algorithm used, as well as the number of records and type of data used [59,60,61]. Therefore, it is appropriate to be cautious about determining which values characterize models with good discrimination. And this is why the significance of our models was tested by the use of random models as described by Raes and ter Steege [59]. Model AUC values were compared to the 95 percentile of the null AUC frequency distribution.

Using SAGA GIS, raw environmental data was extracted from all raster layers at species occurrence records. To minimize the number of variables by discarding these which were highly correlated (r > 0.75), a Spearman rank correlation was performed in the R software (version 3.1.1) [62] using Rattle package (version 3.0.2 r169) [63]. The variables that did not have any significant contribution to the model were removed. Table 1 shows which variables were used in the modelling for continents and for the whole world. Modelling was performed separately for the continents and separately for the whole distribution to examine whether there are significant differences in climatic preferences.

**Table 1:** Variables selected for the modelling. Variables with explanations and altitude ranges for the localities with occurrence of the representatives of the genus *Sirthenea*. Temperatures given in °C; precipitations given in mm; vegetation cover (Tree) given in %.

Variables*	Range	Min.	Max.	Median	Mean
**Bio02**	Africa	6.4	16.6	10.0	10.5
	Americas	6.4	16.7	10.4	104
**Bio06**	Americas	-9.5	22.6	13.7	158
	World	-12.9	22.7	14.7	12.4
**Bio07**	Africa	9.1	28.2	14.5	15.7
	Asia and Australia	7.4	41.6	21.6	19.9
**Bio08**	World	5.1	29.6	24.8	24.1
**Bio10**	Americas	12.7	30.0	25.6	26.3
**Bio11**	Asia and Australia	-6.0	26.9	14.9	17.5
**Bio12**	Africa	456	3354	1601.5	1734.3
	Americas	197	7429	1748.4	1508
	Asia and Australia	106	4709	1981.7	1776.5
	World	195	7294	1509	1721.3
**Bio13**	Africa	109	498	301	305
	Asia and Australia	31	1193	365.2	343
	World	33	786	248	268.4
**Bio15**	Africa	24	122	70	70.3
**Bio18**	Africa	99	1257	506	626.1
	Americas	30	1490	431.8	388
	Asia and Australia	1	2384	690.2	693.5
	World	30	1865	477	513.6

*Bio02 –mean diurnal range; Bio06 –minimal temperature of coldest month; Bio07 –temperature annual range; Bio08 –mean temperature of wettest quarter; Bio10 –mean temperature of warmest quarter; Bio11 –mean temperature of coldest quarter; Bio12 –annual precipitation; Bio13 –precipitation of wettest month; Bio15 –precipitation seasonality (coefficient of variation); Bio18 –precipitation of warmest quarter

Because species occurrence points came from museum data and were not collected randomly, a bias file was provided during Maxent modelling. The bias grid file was generated in SAGA GIS: the species records were weighted by a Gaussian kernel with a standard deviation (SD) of 200 km (by Kernel Density Estimation). In order to avoid extreme values, the resulting grid was scaled to have a maximum of 21 and a minimum of 1 (by Grid Normalisation) [64].

### Terrestrial ecoregions and zoogeographical regions

We assigned the points of occurrence of discussed heteroptera to the terrestrial ecoregions. Terrestrial ecoregions were modified by The Nature Conservancy (TNC–an American charitable environmental organization; http://maps.tnc.org/files/metadata/TerrEcos.xml), based on Olson and Dinerstein [65], Bailey [66] and Wiken [67]. This biogeographic regionalization contains 814 terrestrial ecoregions classified into 14 different biomes. The aim of using these data is to determine which main plant communities species of *Sirthenea* are associated with. New zoogeographical divisions (based on Procheş and Ramdhani [68]), which is divided into regions and subregions, was used to define chorology of the species.

## Results

### Evaluation of the model and the importance of environmental predictors

Four models were analyzed: three for continents and one for the whole world. The AUC values for Africa, Americas, Asia, Oceania and the whole world are given, respectively: 0.898 (standard deviation = 0.134 which is 13.4%), 0.874 (SD = 0.040, i.e. 4.0%), 0.916 (SD = 0.036, i.e. 3.6%) and 0.878 (SD = 0.031, i.e. 3.1%). Both the training and test AUC values of our models were significantly different from random. Based on the publication of Fielding and Bell [58], these values indicate that the models are characterized by a good and very good discriminatory power. The jackknife test (for details and other Maxent model outputs see S3 Appendix) shows that there were common, important variables shared by these models: annual precipitation (Bio12), precipitation of the wettest month (Bio13) and precipitation of the warmest quarter (Bio18). Other variables used in the models are: mean diurnal range (Bio02), minimal temperature of the coldest month (Bio06), annual temperature range (Bio07), mean temperature of the wettest quarter (Bio08), mean temperature of the warmest quarter (Bio10), mean temperature of the coldest quarter (Bio11) and precipitation seasonality (Bio15).

### Ecological niches and potential species group distributions

The arithmetic mean is not as resistant to outliers as the median, which is why the resulting maps show the median of the 50 model replicates output grids. See S3 Appendix for plots that show how the distribution of occurrence records of the representatives of *Sirthenea* refers to the used predictors.

**Fig 2:**
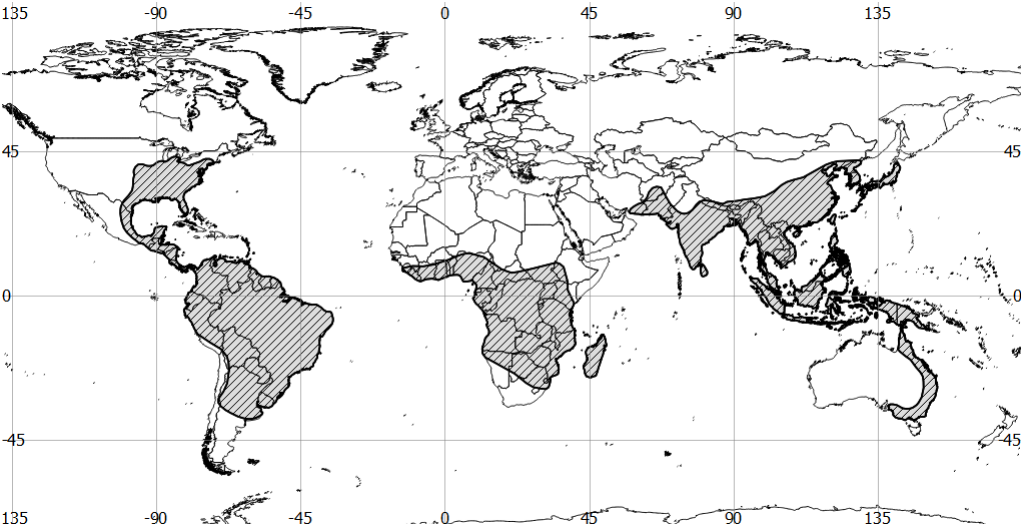
Distribution of the genus *Sirthenea*.

According to the model, the most favourable habitat conditions can be found roughly between the 43^th^ parallel North and 42^th^ parallel South (Fig 2). Individual favorable habitats are described below and are divided by continents.

North and Central America: the most favorable habitats designated by the model are in the areas of the Gulf Coastal Plain, which extends around the Gulf of Mexico in the Southern United States and eastern Mexico. The model has appointed suitable habitats across most of the region: from the western Florida Panhandle, through southern Alabama, southwest Mississippi, part of Tennessee and Kentucky, southwest Arkansas, Louisiana and westernmost Texas in the United States. It continues along the Gulf in northeastern and eastern Mexico, through Tamaulipas, Veracruz, Tabasco and the Yucatán Peninsula. The model also includes the east coast of the United States (Atlantic Coastal Plain), from New York to Florida. Together, the Atlantic Coastal Plain and the Gulf Coastal Plain are the largest physiographic region of the United States. Additional suitable habitats can be found in Oklahoma, Kansas and Missouri. The model also suggests areas around the common borders of California, Nevada and Arizona, as well as the west coast of Mexico, but there is a natural geographic barrier that is the Mexican Plateau, which is flanked by the Sierra Madre Occidental and the Sierra Madre Oriental.

Other most favorable habitats have been designated by the model in the areas of Belize, northeastern Guatemala, Honduras, Nicaragua, Costa Rica and Panama, as well as in the Caribbean (Antilles, the Bahamas, the Cayman Islands and parts of islands in the Caribbean Sea).

South America: the model has appointed the most favorable habitats at the west coasts of Colombia, Ecuador and Peru, as well as other areas within these countries, with the exceptoin of the Andes. Other areas designated by the model is almost the entire area of Guyana, Suriname and French Guiana, and further areas in the central region of Venezuela, but without the Guiana Highlands and the southern part of the country. In Brazil, favorable habitats were located in the Amazon Basin, the entire length of the east coast and the Pantanal. In addition, the central part of Bolivia (particularly around the city Cochabamba), the eastern part of Paraguay and the northern part of the Pampas on the border between Argentina, Uruguay and Brazil were indicated as favorable areas by the model.

Africa: favorable habitats start at the north of the continent from the coast of Gambia and Guinea-Bissau, then pass through the Upper Guinea (a large plain extending from southwestern Guinea through Sierra Leone, Liberia, southeastern Guinea, Ivory Coast and southwestern Ghana), Dahomey Gap (which extends to the coast in Ghana, Togo and Benin) and continue through the Lower Guinea (extending along the eastern coast of the Gulf of Guinea from eastern Benin through Nigeria and Cameroon). Other favorable habitats have been appointed through almost the entire Congo Basin and the Atlantic Equatorial coastal forests–ecoregion of central Africa, which includes hills, plains and mountains of the Atlantic coast of Cameroon, Equatorial Guinea, Gabon, Republic of the Congo, Democratic Republic of the Congo and Angola. Other areas designated by the model can be found in the vicinity of Lake Victoria in Uganda and Tanzania, in the south of Tanzania, the eastern part of Malawi, and the northern and central part of Mozambique, in particular, on the border with Zimbabwe. In Madagascar, the model has appointed particularly favorable habitats in the north and on the east coast of the island, as well as on Reunion, Mauritius, Comoro Islands and Mayotte.

Asia, Australia and Oceania: the model has appointed the most suitable habitats along the whole Western Ghats (mountain range at the western coast of the Indian peninsula), including the Cardamom Hills. Also appointed a favorable areas on the Island Ceylon, on the foothills of the Himalayas (India, Nepal and Bhutan), within the Patkai (the hills on India’s north-eastern border with Burma) and the Shan Hills–a mountainous area which extends through Yunnan (a province of the People’s Republic of China) to Burma and Thailand. Other suitable habitats were appointed by the model in the mountain chain known as the Tenasserim Hills (which extends by Burma, Thailand and Malaysia) and its foothills, as well as the mountain ranges between southern China, northern Laos and northern Vietnam, Xiangkhoang Plateau and Cammon Plateau in Laos, Western Highlands (also called Central Highlands) in Vietnam and Annamite Range (or Annamite Cordillera), which extends through Laos, Vietnam and a small area in northeast Cambodia. There is also a lot of suitable habitats in the southern part of China (Yunnan-Guizhou Plateau and Southeast China Hills regions), in the south of North Korea and all over of South Korea, Japan (the island of Kyushu, Shikoku and Honshu), Taiwan, Philippines, the islands of the Greater Sunda Islands and the Lesser Sunda Islands (including Sumatra, Java, Borneo and Sulawesi)–all included in Indonesia, both islands of New Zealand, plus islands in the Pacific Ocean belonging to the Melanesia, Micronesia and Polynesia. Suitable habitats in Australia have been appointed in the northern state of Queensland and along the east coast in the foothills of the Great Dividing Range.

The results from the model for the whole world coincides with the results of the different models for the continents. A greater difference is only observed in the territory of Brazil, where the model for the whole world determines almost the entire territory of the country as suitable for these true bugs. In addition, a model for the whole world has expanded the boundaries of the suitable habitat areas, but mainly in terms of acceptable habitat (which have less than 50% chance of occurrence of favorable conditions).

**Fig 3:**
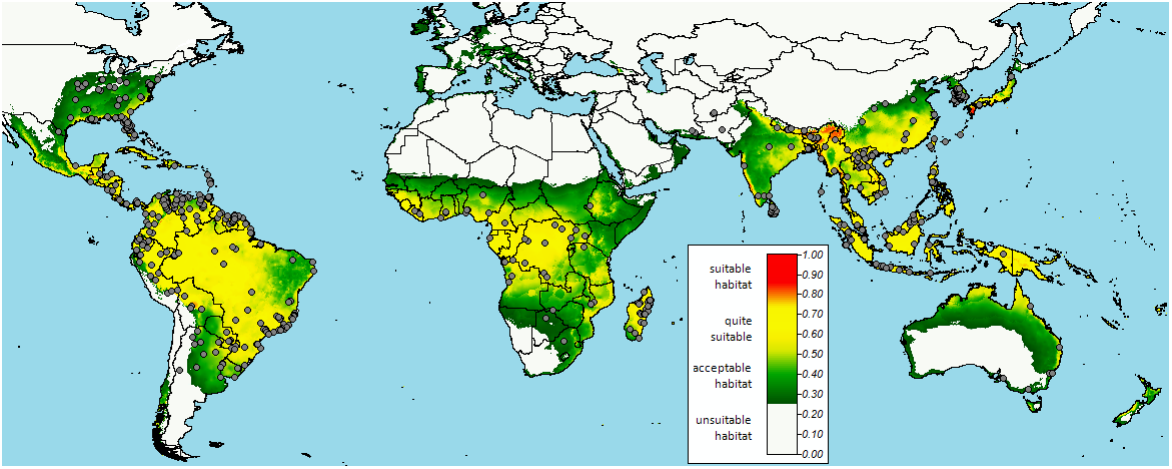
Predicted suitable habitats for the genus *Sirthenea* in the world. The legend indicates the presence suitability in percentage. Inland continuous lines represent country boundaries. Points represent known distribution of representatives of the genus Sirthenea.

For details of all the places specified by the model as potentially suitable see Fig 3 and S4 Appendix.

### Climatic preferences

Because of the continental and world range used in the model, the most important factors affecting species distributions were climate variables. Possible climatic preferences of *Sirthenea* species were inferred by comparing potential ecological niches to the Köppen-Geiger climate classification. The use of biotic variable was not possible in this case.

The analysis of climate types in the known occurrence places of *Sirthenea* representatives indicate that, in particular, they prefer tropical and temperate climates (those two types together constitute 89.7% of the four main types for the whole world). However, depending on the part of the world, the dependence of the percentage of each type of climate has slightly changed. In Africa, the largest share have a tropical climates (47 occurrences = 79.6%) and then temperate (8 occurrences = 13.6%), while the smallest share a dry climates (4 occurrences = 6.8%). In the Americas, tropical climates also have the largest share (195 occurrences = 60.0%) followed by a temperate climates (107 occurrences = 32.9%), while the sharing of dry and continental climates is comparable (11 and 12 occurrences respectively = 3.4% and 3.7%). In Asia, Australia and Oceania, proportions are more balanced: 50.3% for tropical climates (84 occurrences), 28.7% for temperate climates and 17.9% for continental climates. However, in Asia, a lot of occurrence places are concentrated in South Korea, where the continental climate type dominates. Reducing the proportion of this area significantly affects the increase in the share of tropical and temperate climates.

The model as well suggests that suitable habitats for the representatives of *Sirthenea* should be sought mainly in tropical and temperate climates.

Climatic diagrams in S3 Appendix show how the average temperature and precipitation are correlated with each other for different regions of the world.

### Biomes, zoogeographical regions and representatives of *Sirthenea*

**Fig 4:**
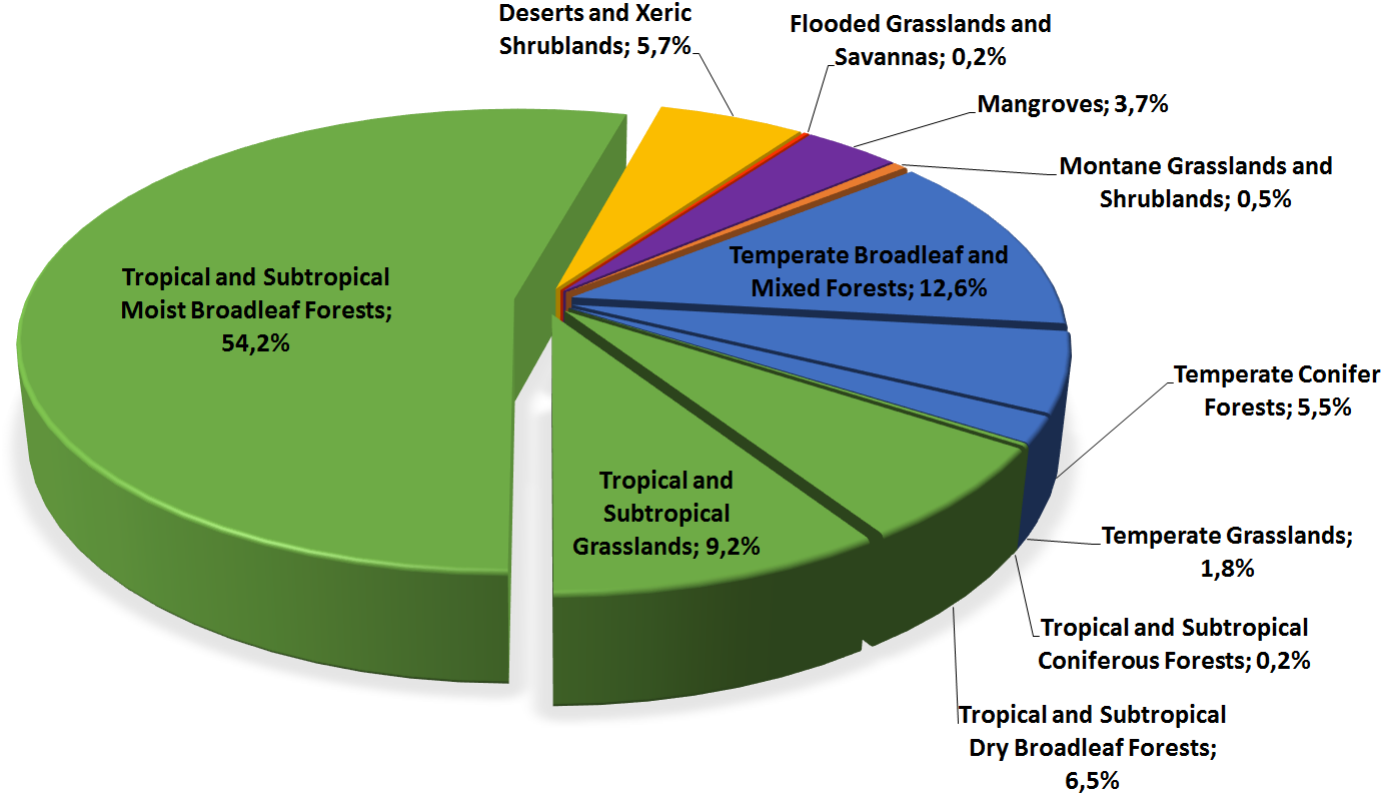
Percentage share of representatives of the genus *Sirthenea* in different biomes.

Among all individuals of the genus *Sirthenea* that were examined, 54.2% are classified in tropical and subtropical moist broadleaf forests biomes (Fig 4). These forests are located in a belt around the equator, mostly in the Congo basin of central Africa and in coastal West Africa, the Amazon basin of South America, in Central America, the Caribbean, parts of the Indian subcontinent, Indonesia and New Guinea. Of the remaining individuals, 12.6% are classified in temperate broadleaf and mixed forest biomes that are most distinctive in central China and eastern North America, whereas 9.2% are in tropical and subtropical grassland, savanna and shrubland biomes that are widespread in Africa, South Asia, the southern United States, and the northern parts of South America and Australia.

**Fig 5:**
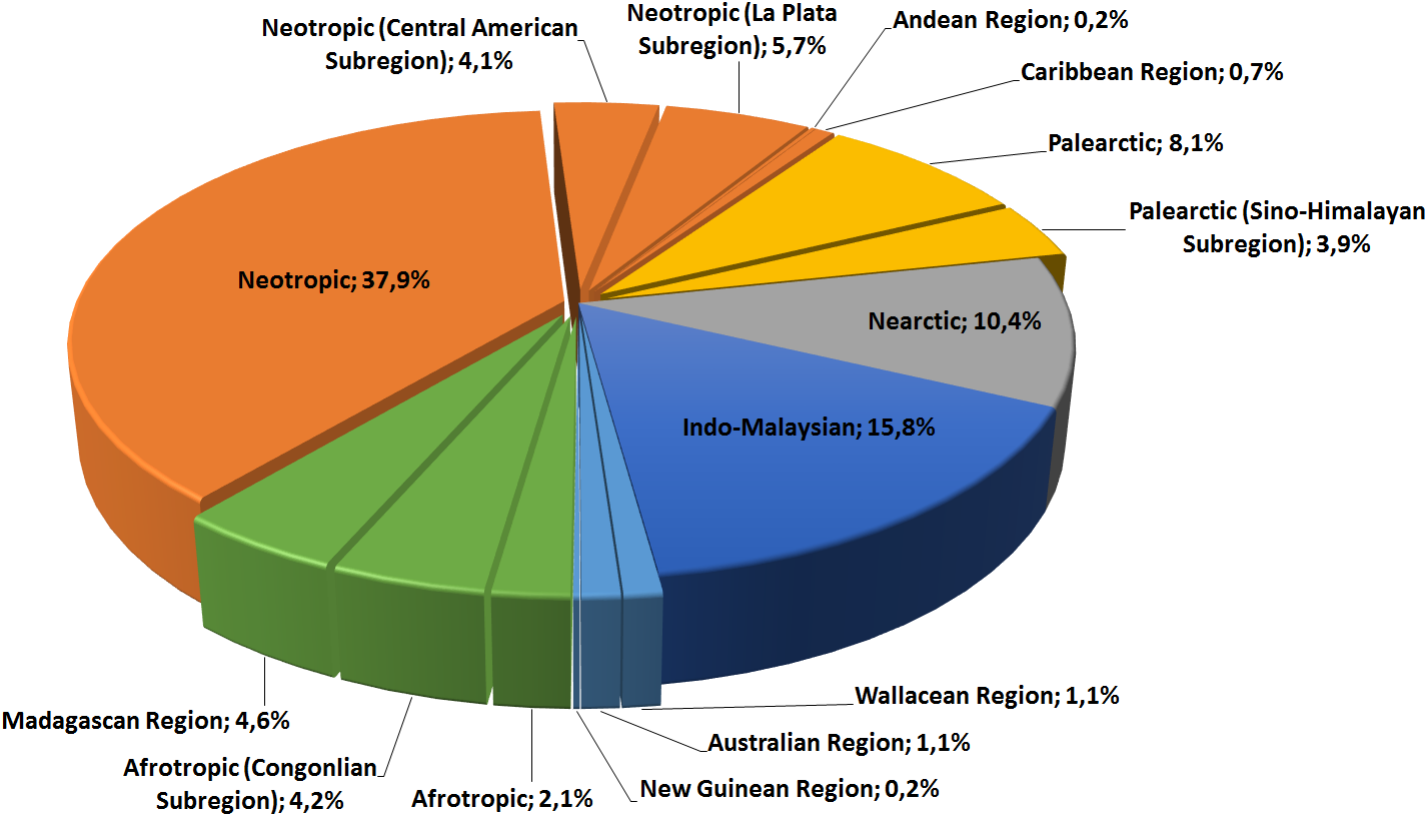
Percentage share of representatives of the genus *Sirthenea* in zoogeographical regions.

**Fig 6:**
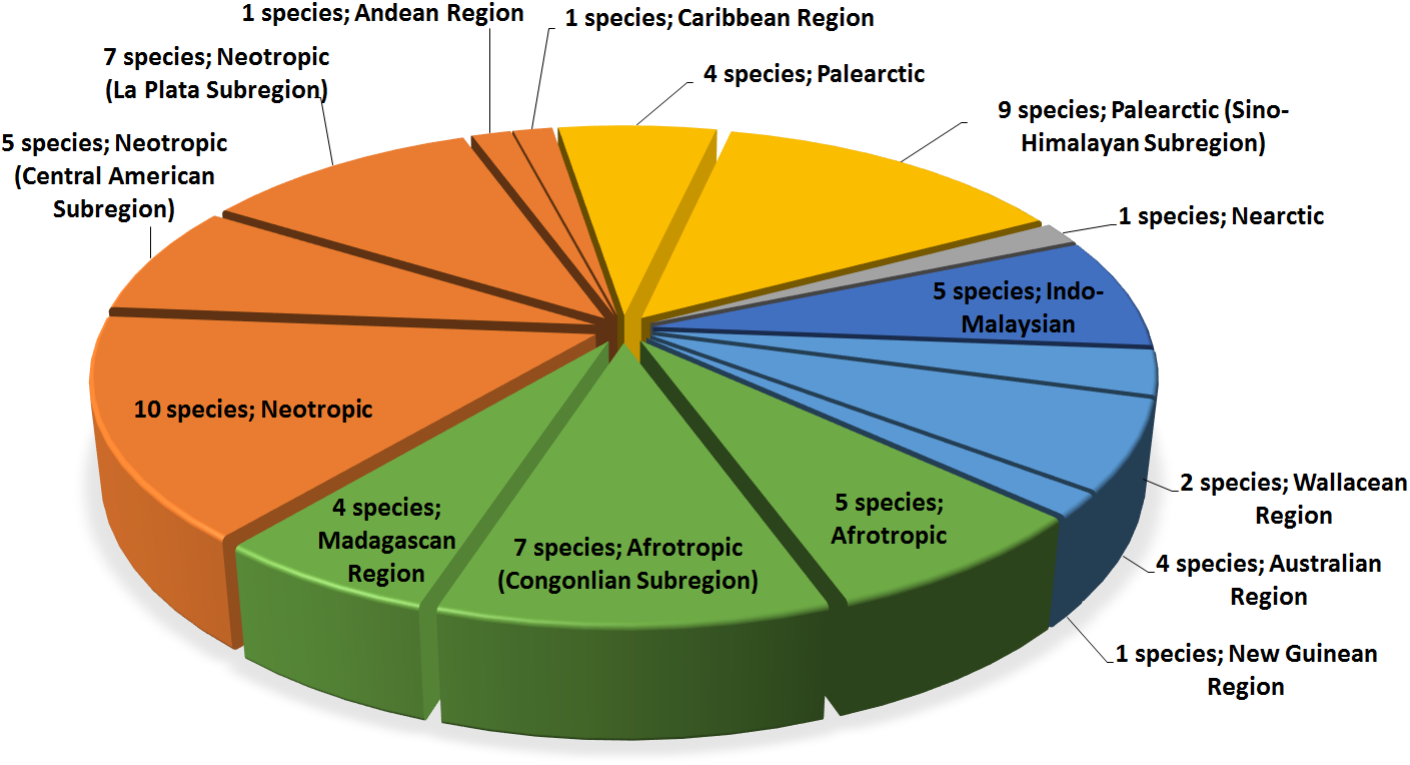
Percentage share of the species of the genus *Sirthenea* in zoogeographical regions.

Representatives of *Sirthenea* are shown together from 15 zoogeographical regions and subregions (Figs 5 and 6). The Neotropical region is the most numerous in terms of species (12 species, which gives 33% among all regions), followed by the Palearctic and Afrotropical regions (10 and 8 species; 28% and 22%). The assignment of terrestrial ecoregions and biomes to the zoogeographical regions for each species of *Sirthenea* can be found in Table B in S2 Appendix.

## Discussion

One of the most important issues concerning the genus *Sirthenea* is its presence in almost all zoogeographical regions of our planet. After gathering all available data, we prepared a model of the distribution and habitat preferences for the mentioned genus. This type of research on the representatives of the subfamily Peiratinae occurring in the Neotropical region has been published by Coscarón & Morrone [69]. Nevertheless, compared to all the genera described so far belonging to the Peiratinae (or even within the family Reduviidae), the distribution of *Sirthenea* seems to be unique (Fig 3 and S1 Appendix). Using the information provided on the labels beneath each individual we examined, we were not only able to prepare the model of distributional patterns and climate preferences but also to obtain and provide new information on the biology of *Sirthenea*. A vast majority of representatives in this genus prefer low-lying areas, which is consistent with observations by Willems [34], but most of the specimens (58%) were collected at heights of 0–200 m.a.s.l. Accordingly, successive range heights were represented by: 12.5% of specimens at heights of 201–400 m., 8.5% of specimens at heights of 401–600 m., 7.8% of specimens at heights of 601–800 m., 6.2% of specimens at heights of 801–1000 m., 2.9% of specimens at heights of 1001–1200 m., 3.5% of specimens at heights of 1201–1400 m., and only a few specimens were collected at heights over 1400 m.a.s.l. One specimen of *Sirthenea peruviana* Drake and Harris, 1945 [70] was collected in Peru at 3328 m above sea level, thus representing the highest known locality of the genus.

According to the data contained on legitimate labels, we can conclude that all representatives of this genus are preying mainly at night (most of the specimens are collected at different kind of light traps) on the soil surface often covered by fallen leaves or other plant parts, which confirms the information contained in Readio [42] and Willemse [34]. Moreover, all the data contained on legitimate labels, as well as in published papers [1,34–41,48,49], allowed us to make a model of the distribution and habitat preferences of *Sirthenea*, thus increasing our knowledge about the biology of representatives of this genus.

The possibilities of using ecological niche modelling methods are still being developed. Furthermore, the weaknesses of this method are still being addressed, and so it is gaining credibility. A big plus of MaxEnt software is the ability to use present-only data, because such data we are able to obtain from the field survey, literature and museum collections. We used ecological niche modelling method and MaxEnt software in our study to provide some new information about potential distributions, as well as climate preferences of *Sirthenea*. While many specimens came from the same location, duplicate records were removed to prevent unnecessary strengthening of points and to avoid model disturbances by strong intercorrelations [71]. A bias grid was also used to upweight records with few neighbors in geographic space [64]. Moreover, Elith et al. [72] noted that the probability of a species occurrence in suitable areas at an average site was 0.5. Therefore, in the results, suitable habitats with probabilities above 0.5 were listed.

Biogeographical and ecological studies are carried out at different scales, which are also important in ecological niche modelling [73]. Because the studies were conducted at the continental and global scale, it was possible to determine the climatic preferences of the genus. The precipitation of the warmest quarter (Bio18) is one of the most important bioclimatic variables in all conducted models, followed by annual precipitation (Bio12). Among the temperature variables, minimal temperature of the coldest month (Bio06), temperature annual range (Bio07 –a function of the maximum temperature of the warmest month and minimum temperature of the coldest month) and mean temperature of the warmest quarter (Bio10) were important. The average annual precipitation for all members of the genus *Sirthenea* in the world amounted to 1721 mm/m^2^ (median = 1509, min. = 195, max. = 7294), while the average precipitation in the warmest quarter amounted to 514 mm/m^2^ (median = 477, min. = 30, max. = 1865). As the model suggests, suitable habitats for this genus can be found in the tropical climate, which is characterized by permanently high temperatures (above 18°C (64°F) throughout the year), a distinct dry season and precipitation of about 60–100 mm/m^2^ (average annual rainfall). A large part of the habitats was also indicated by the model in areas with temperate climates, where an average temperature in the warmest months is above 10°C (50°F). In this type of climate, the model suggests mainly humid subtropical (Cfa, Cwa) and oceanic (Cfb, Cfc, Cwb, Cwc) subtypes. The first one is characterized by hot, humid summers and mild to cool winters, while the second one has warm (not hot) summers and cool (not cold) winters. Similar results were obtained in our other works regarding climate preferences of the true bugs of the family Reduviidae, as well as other researchers [74–78].

Some of the data from unidentified material confirmed the obtained results, though they were not used in the modelling process. The model clearly indicated the presence of favourable environmental conditions in Sierra Leone, Ivory Coast, Benin, Burundi and Zanzibar in Africa, Sri Lanka, Kei Islands in Asia and Oceania, as well as the areas of Australia where only states were mentioned, Jamaica and the states of the United States, which were listed without a specific location.

Next to the abiotic variables, biotic variables can be used in the modelling. For predatory true bugs, such as representatives of the genus *Sirthenea*, the best biotic variable would be the distribution of their prey. Unfortunately, their prey probably belongs to several different species and we do not have access to such data, but we believe that such data may reduce the range of the genus but would help to characterize the habitat better [79].

We also want to pay attention to the fact that even if the model suggests some locations as potentially suitable, the species could be absent [80,81]. This is due to the fact that it is not possible to take into account all potential environmental factors (e.g., barriers to dispersal, lack of prey resources or the presence of another predator), which determine the occurrence of individuals of a species in a particular locality.

## Supporting Information

S1 Appendix Distribution maps of known genera of the subfamily Peiratinae. (PDF)

S2 Appendix Table A. Details of all occurrence localities used in the Maxent model. Table B. Details of belonging of individual species to terrestrial ecoregions. (XLSX)

S3 Appendix Maxent model outputs and climatic diagrams. (PDF)

S4 Appendix Detailed maps of potentially suitable niches for representatives of the genus *Sirthenea*. (PDF)
