# Supplementary material for: Distribution Pattern and Climate Preferences of the Representatives of the Cosmopolitan Genus Sirthenea Spinola, 1840 (Heteroptera: Reduviidae: Peiratinae)
Source: PLoS One. 2015 Oct 23;10(10):e0140801. doi: 10.1371/journal.pone.0140801 (PMC4619806; doi:10.1371/journal.pone.0140801)
Supplement: S1 Appendix — (PDF) [file pone.0140801.s001.pdf]

Supporting Information

Distribution pattern and climate preferences of the representatives of the genus *Sirthenea* Spinola, 1840 (Heteroptera: Reduviidae: Peiratinae)

PLOS ONE

Dominik Chłond*, Agnieszka Bugaj-Nawrocka

Department of Zoology, Faculty of Biology and Environmental Protection, University of Silesia, Katowice, Poland

* Correspondence: Dominik Chłond, Department of Zoology, Faculty of Biology and Environmental Protection, University of Silesia, Bankowa 9, 40-007 Katowice, Poland.

e-mail: dominik.chlond@us.edu.pl; abugaj-nawrocka@us.edu.pl

Supporting Information S1: Distribution maps of known genera of the subfamily Peiratinae

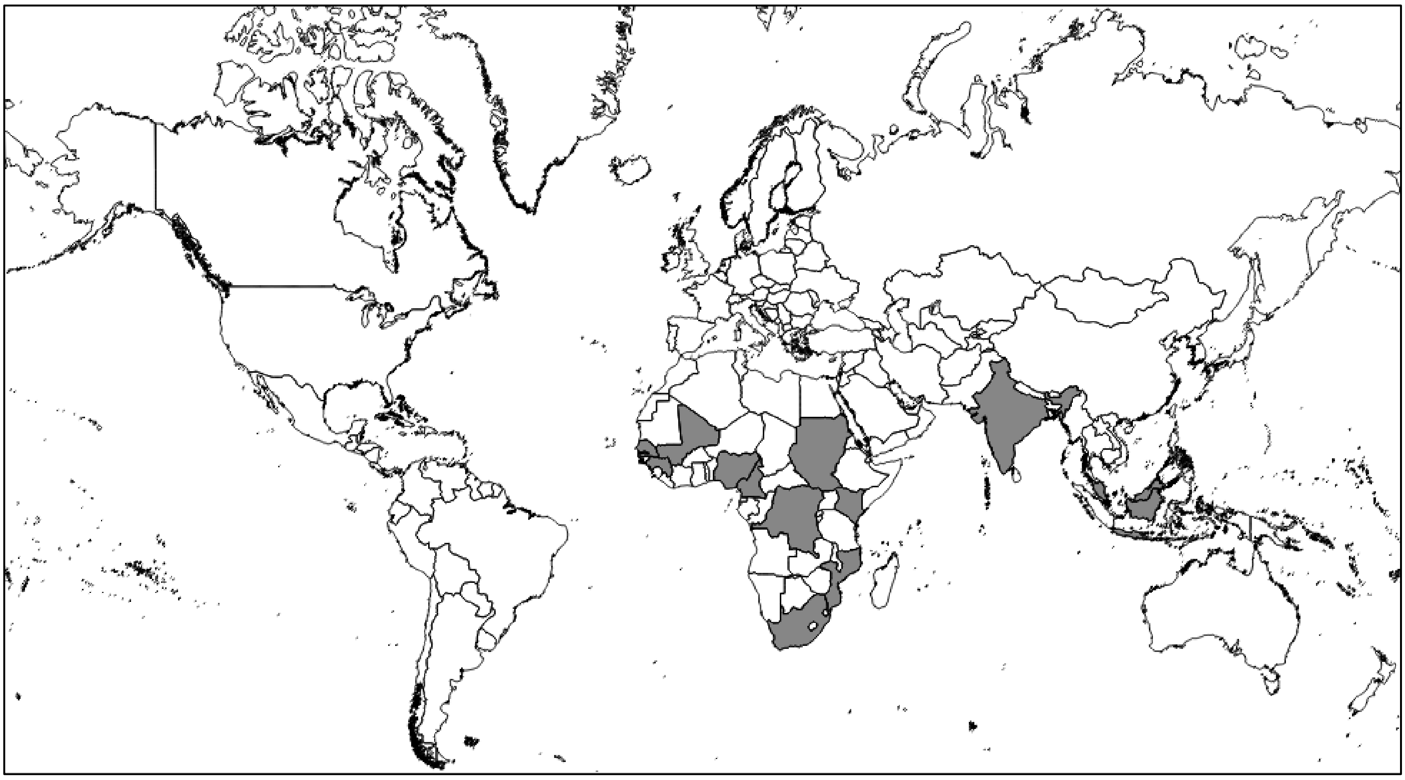

Figure S1.1 Distribution map of the genus *Androclus* (Cameroon, Democratic Republic of the Congo, Guinea, India, Indonesia: Borneo and Java, Kenya, Malaysia: Negeri Sembilan, Mali, Mozambique, Nigeria, Senegal, South Africa, Sudan)

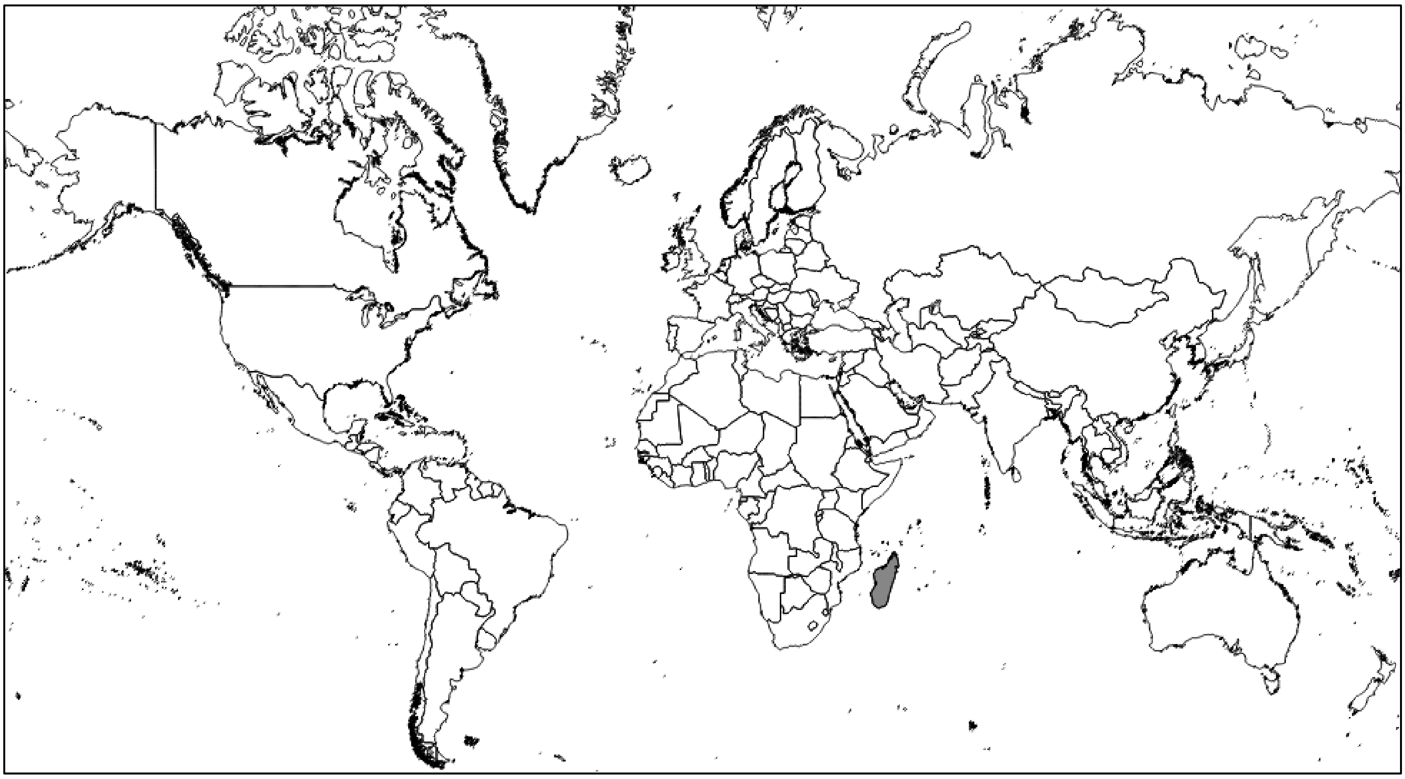

Figure S1.2 Distribution map of the genus *Bekilya* (Madagascar)

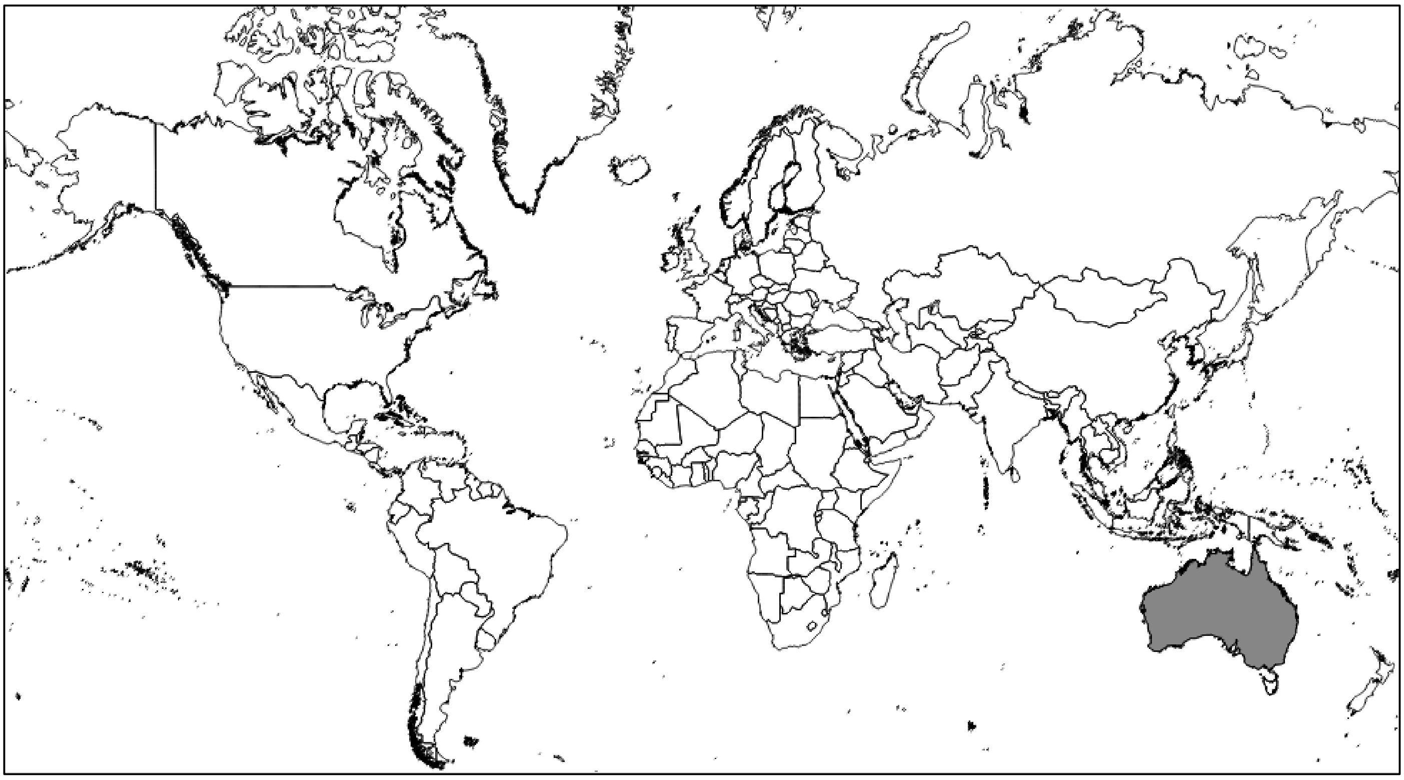

Figure S1.3 Distribution map of the genus *Brachysandalus* (Australia)

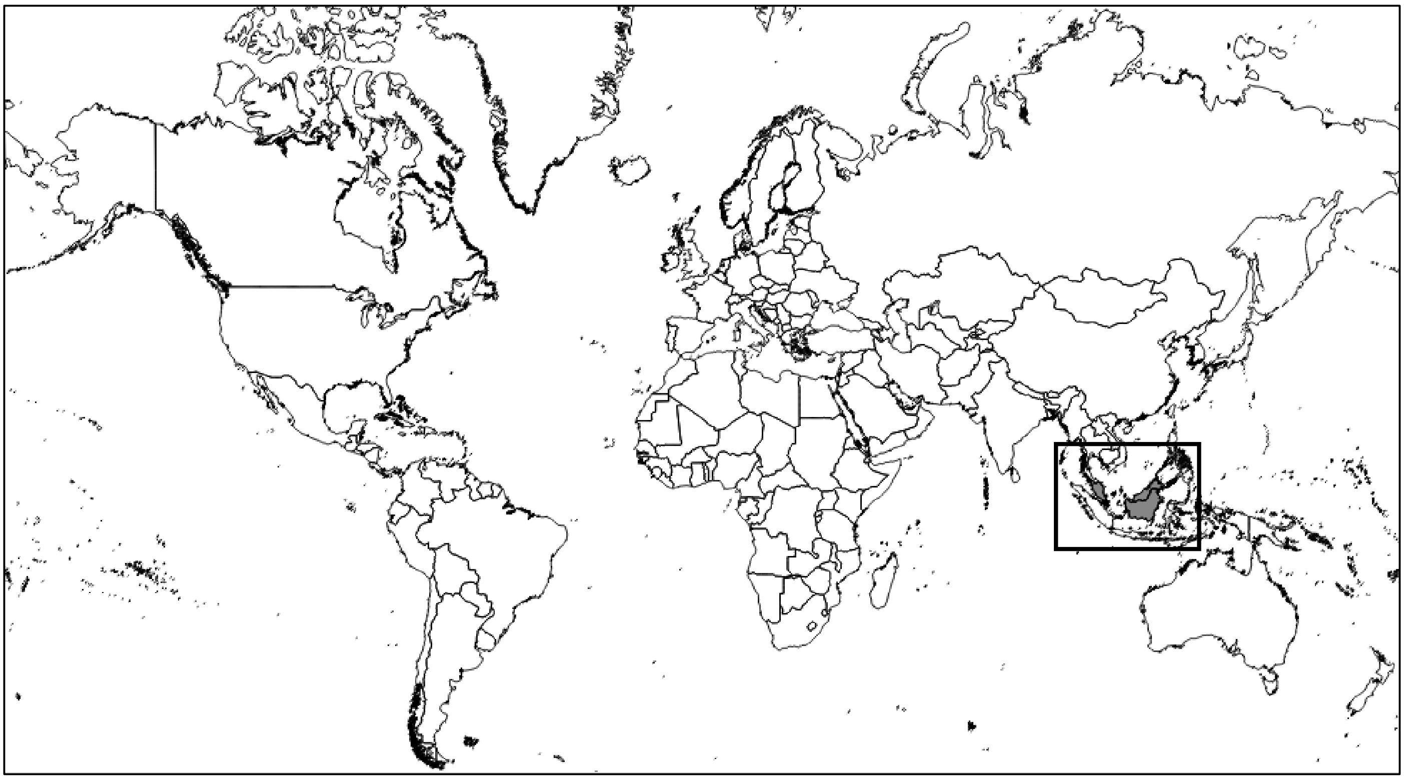

Figure S1.4 Distribution map of the genus *Calistocoris* (Indonesia: Borneo, Malaysia: Pahang and Sarawak)

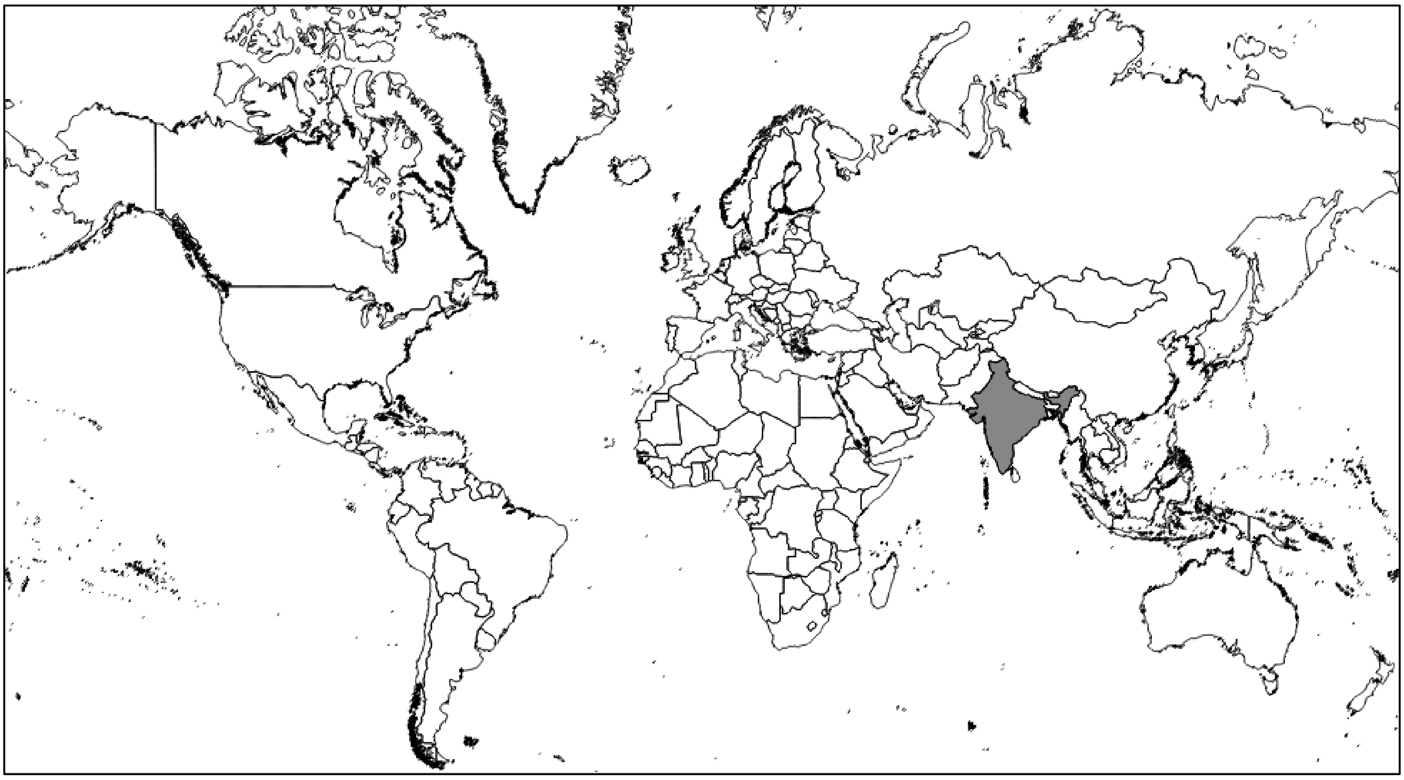

Figure S1.5 Distribution map of the genus *Catamiarus* (India)

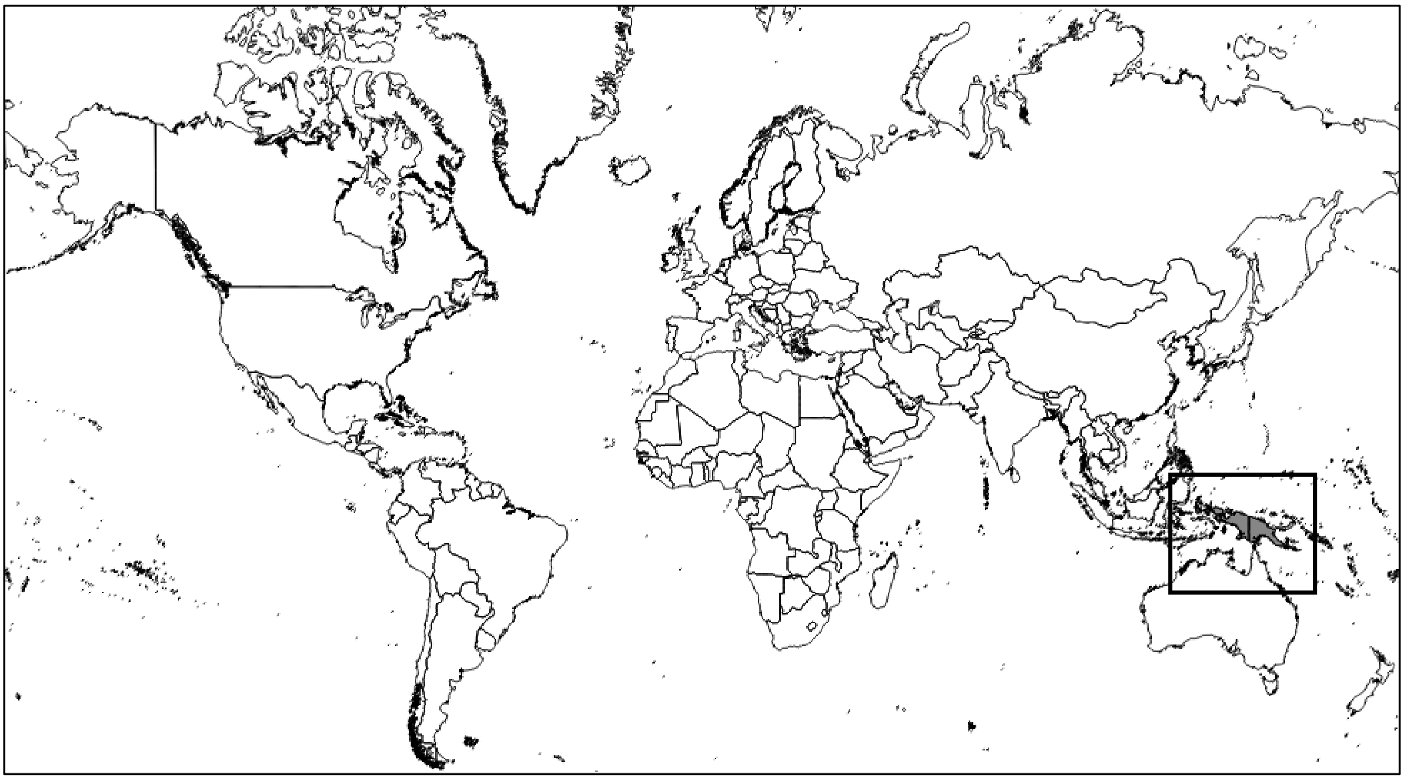

Figure S1.6 Distribution map of the genus *Ceratopirates* (Papua New Guinea)

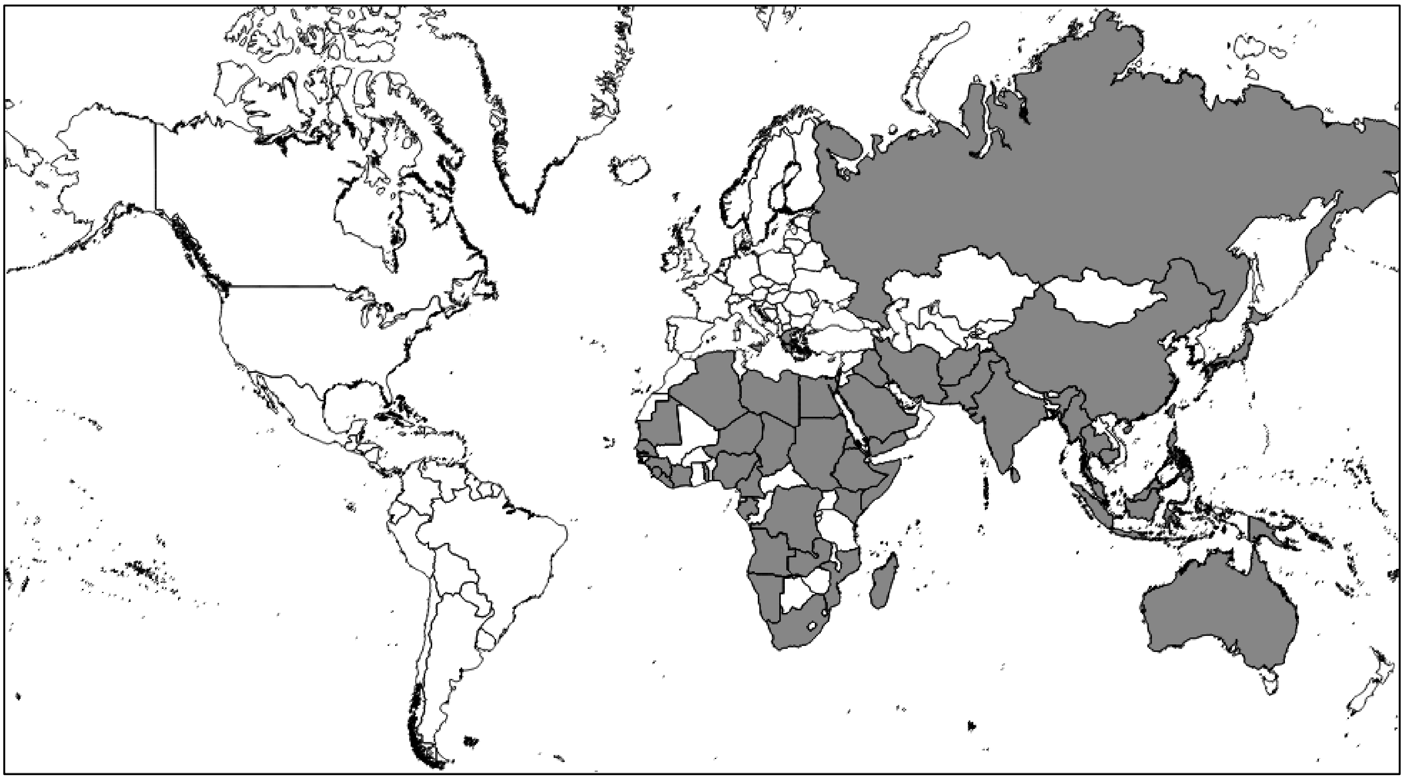

Figure S1.7 Distribution map of the genus *Ectomocoris* (Afghanistan, Algeria, Angola, Australia, Cambodia, Cameroon, Cape Verde, Chad, China, Democratic Republic of the Congo, East Timor, Egypt, Eritrea, Ethiopia, Gabon, Gambia, Greece, Guinea, India, Indonesia, Iran, Iraq, Israel, Italy, Ivory Coast, Japan, Kenya, Liberia, Libya, Madagascar, Malaysia, Mauritania, Mozambique, Myanmar, Namibia, Niger, Nigeria, Pakistan, Papua New Guinea, Philippines, Portugal, Russia, Saudi Arabia, Senegal, Sierra Leone, Somalia, South Africa, Spain, Sri Lanka, Sudan, Taiwan, Tanzania, Thailand, Togo, Yemen, Zambia)

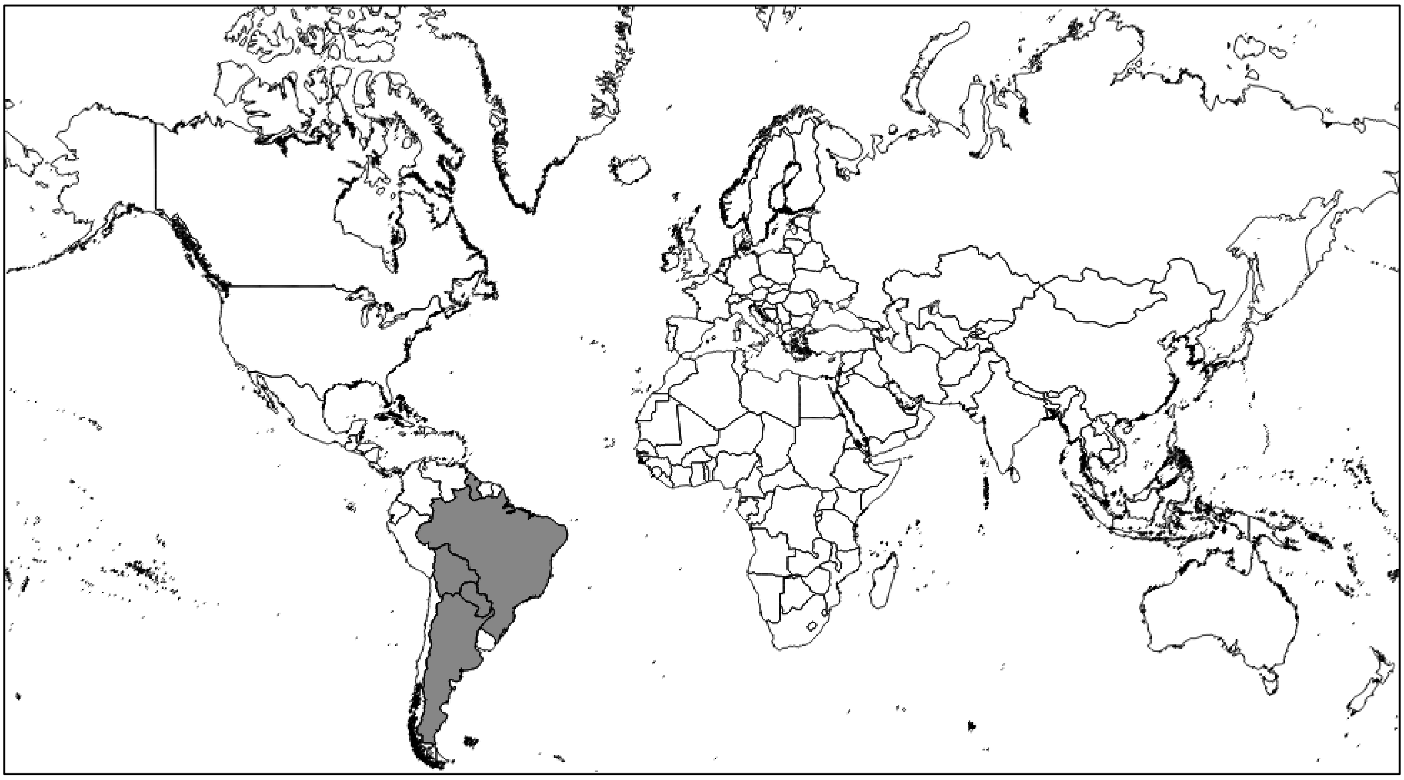

Figure S1.8 Distribution map of the genus *Eidmannia* (Argentina, Bolivia, Brazil, Guyana, Paraguay)

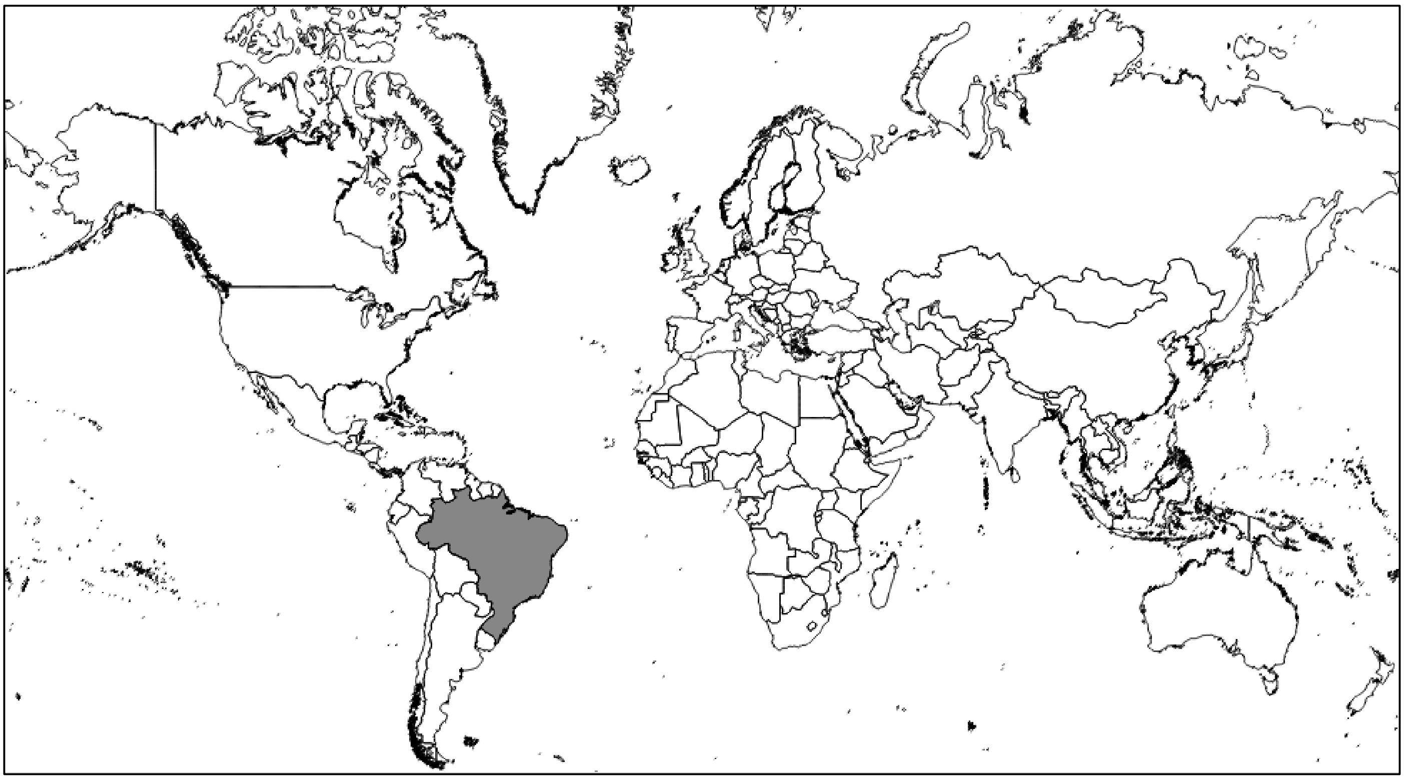

Figure S1.9 Distribution map of the genus *Froeschnerisca* (Brazil, Panama)

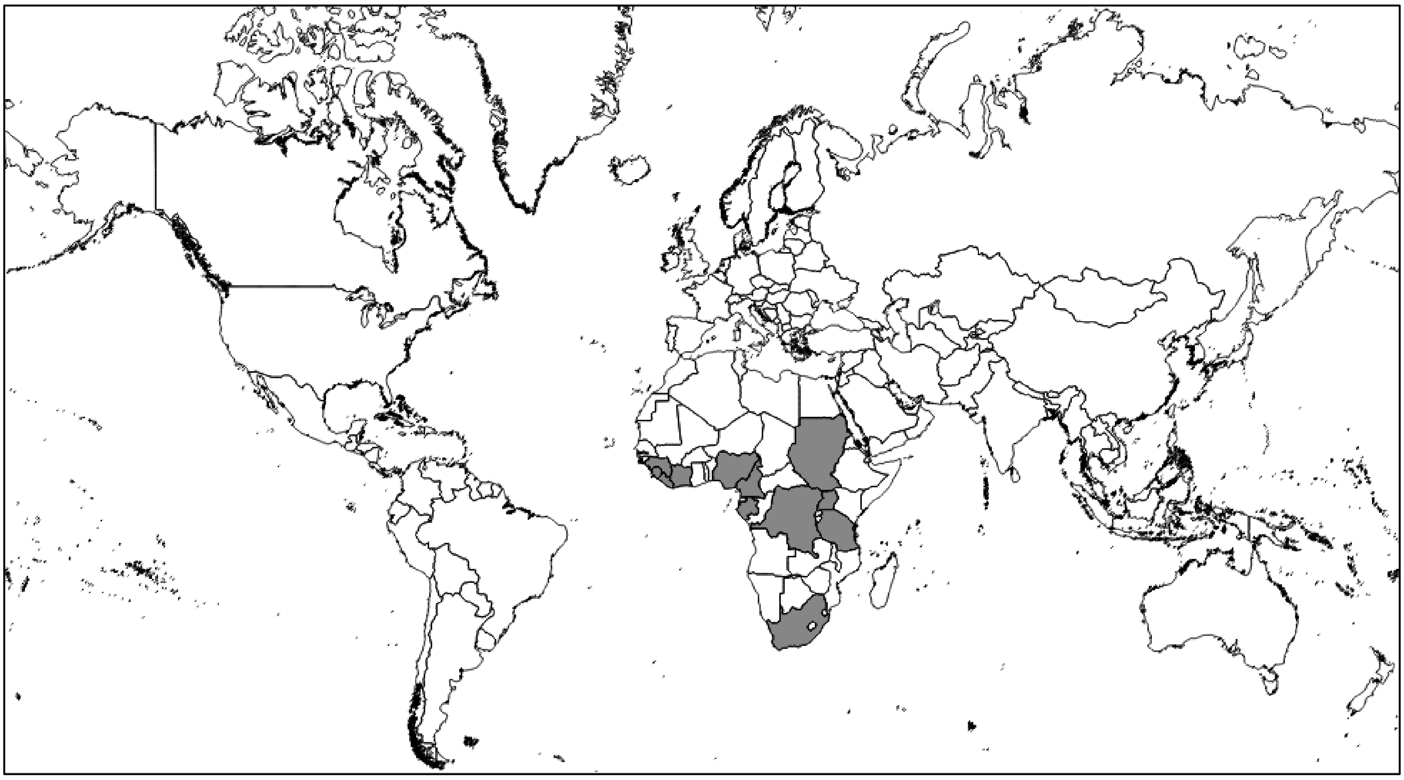

Figure S1.10 Distribution map of the genus *Fusius* (Cameroon, Democratic Republic of the Congo, Gabon, Guinea, Ivory Coast, Liberia, Nigeria, Sierra Leone, South Africa, Sudan, Tanzania, Uganda)

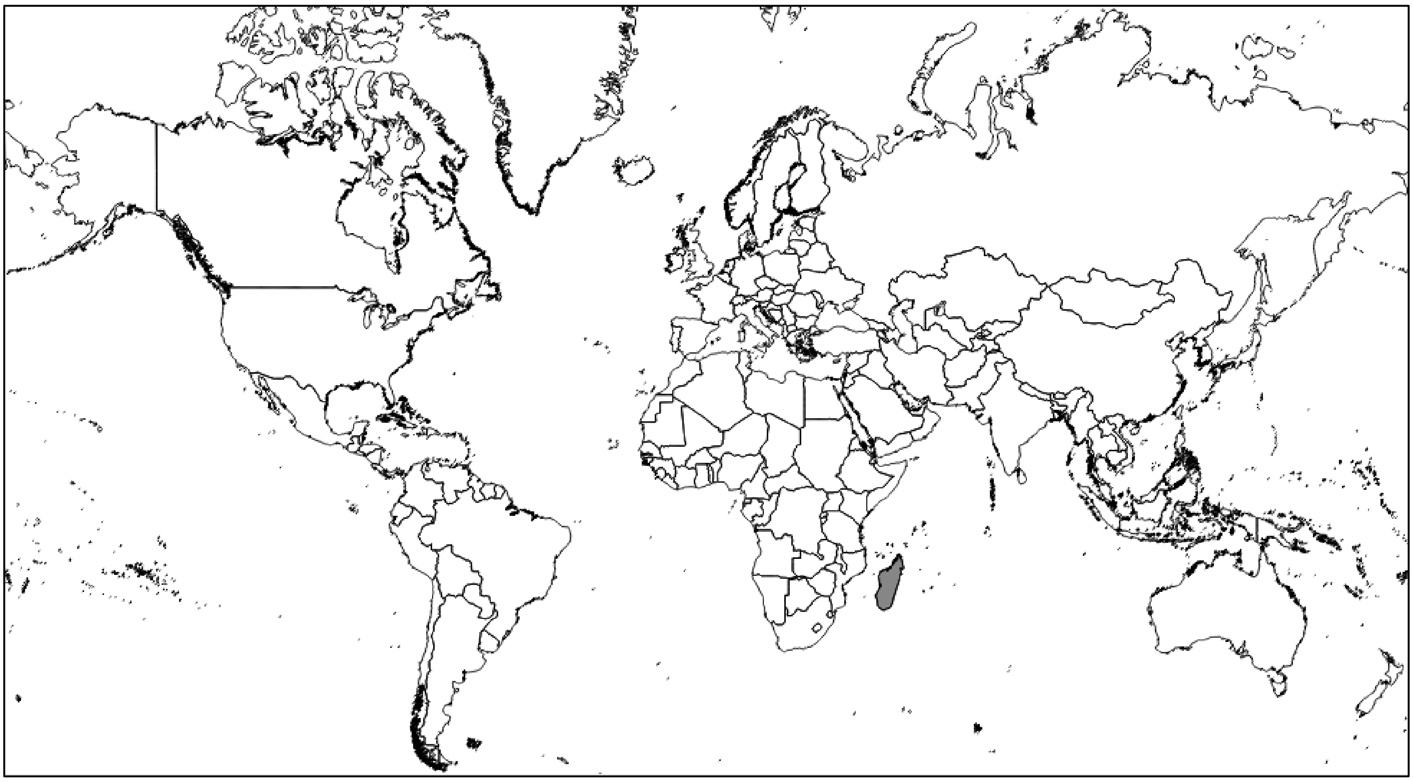

Figure S1.11 Distribution map of the genus *Hovacoris* (Madagascar)

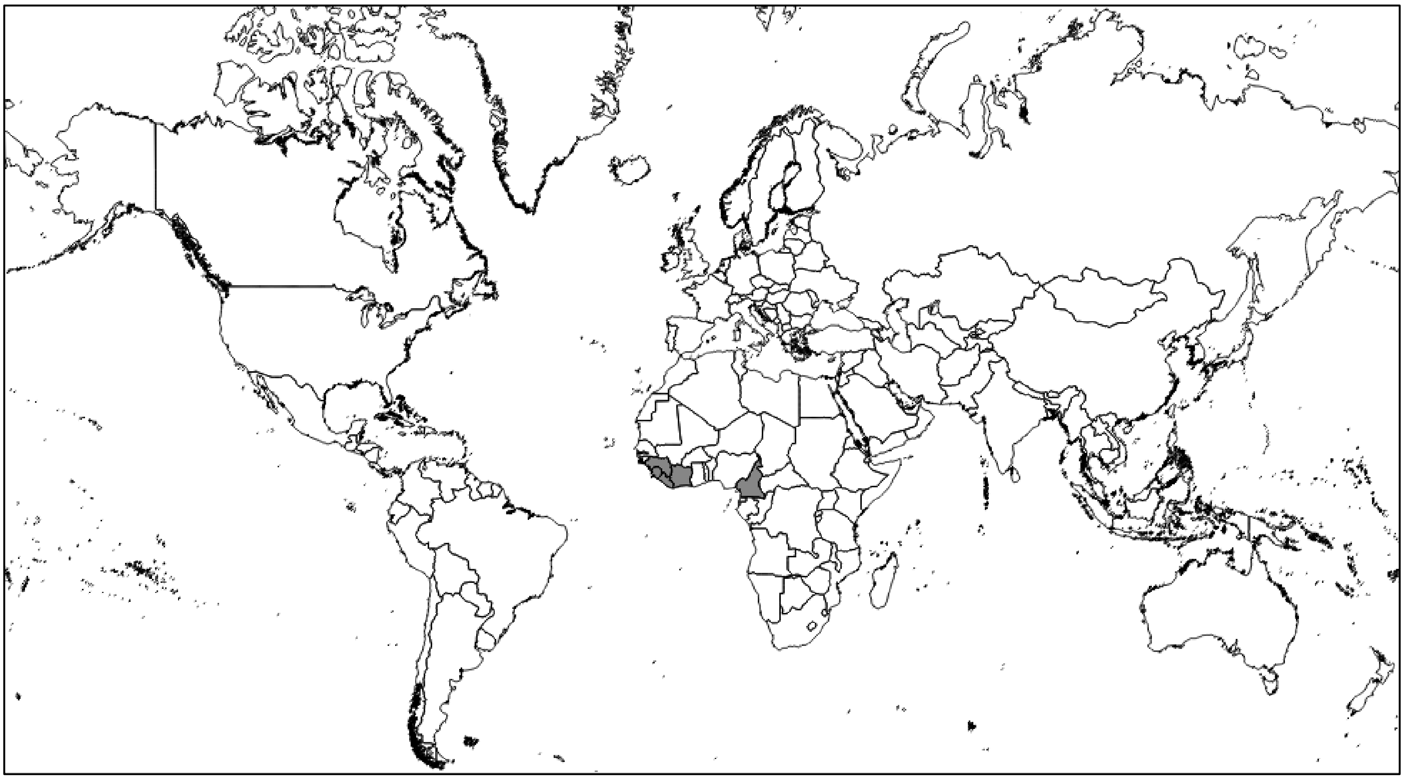

Figure S1.12 Distribution map of the genus *Lamotteus* (Cameroon, Guinea, Ivory Coast, Liberia, Sierra Leone)

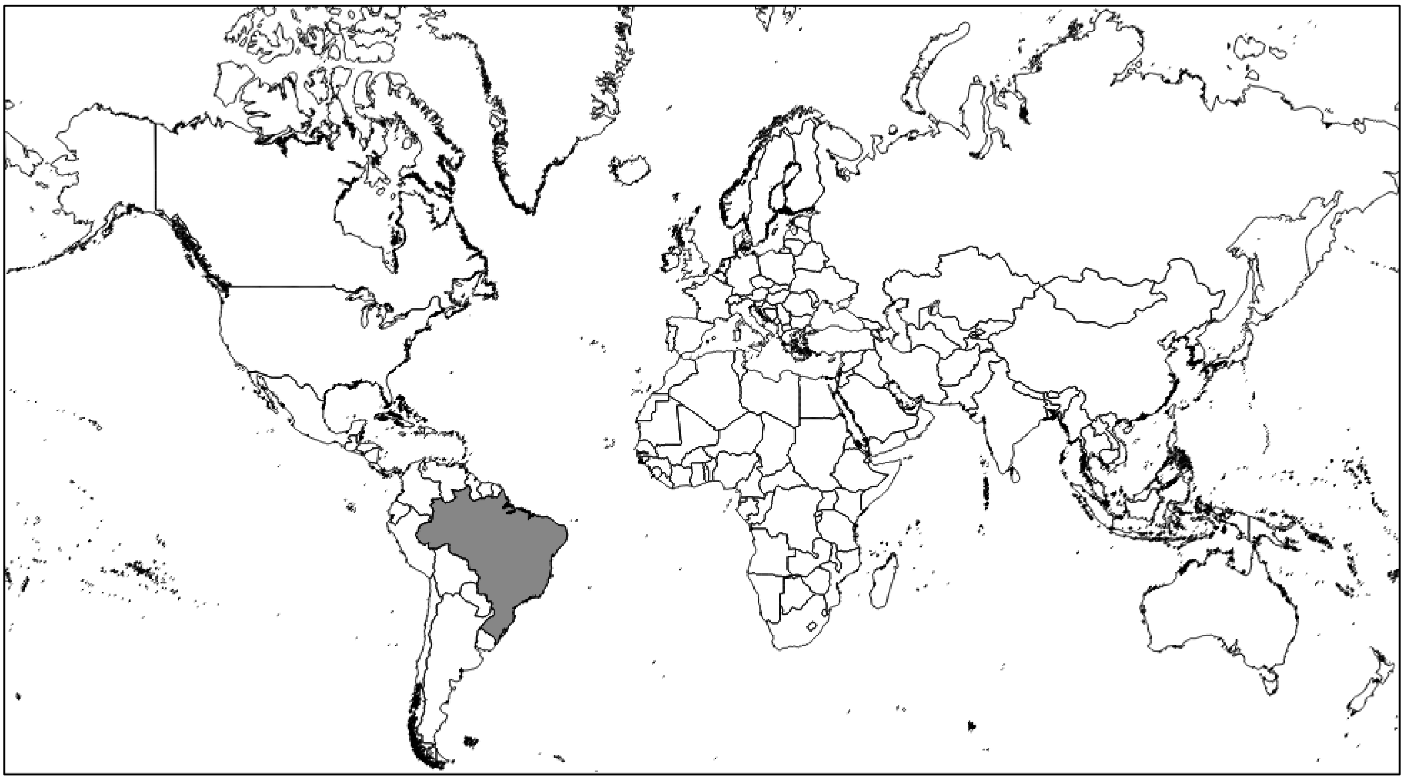

Figure S1.13 Distribution map of the genus *Lentireduvius* (Brazil)

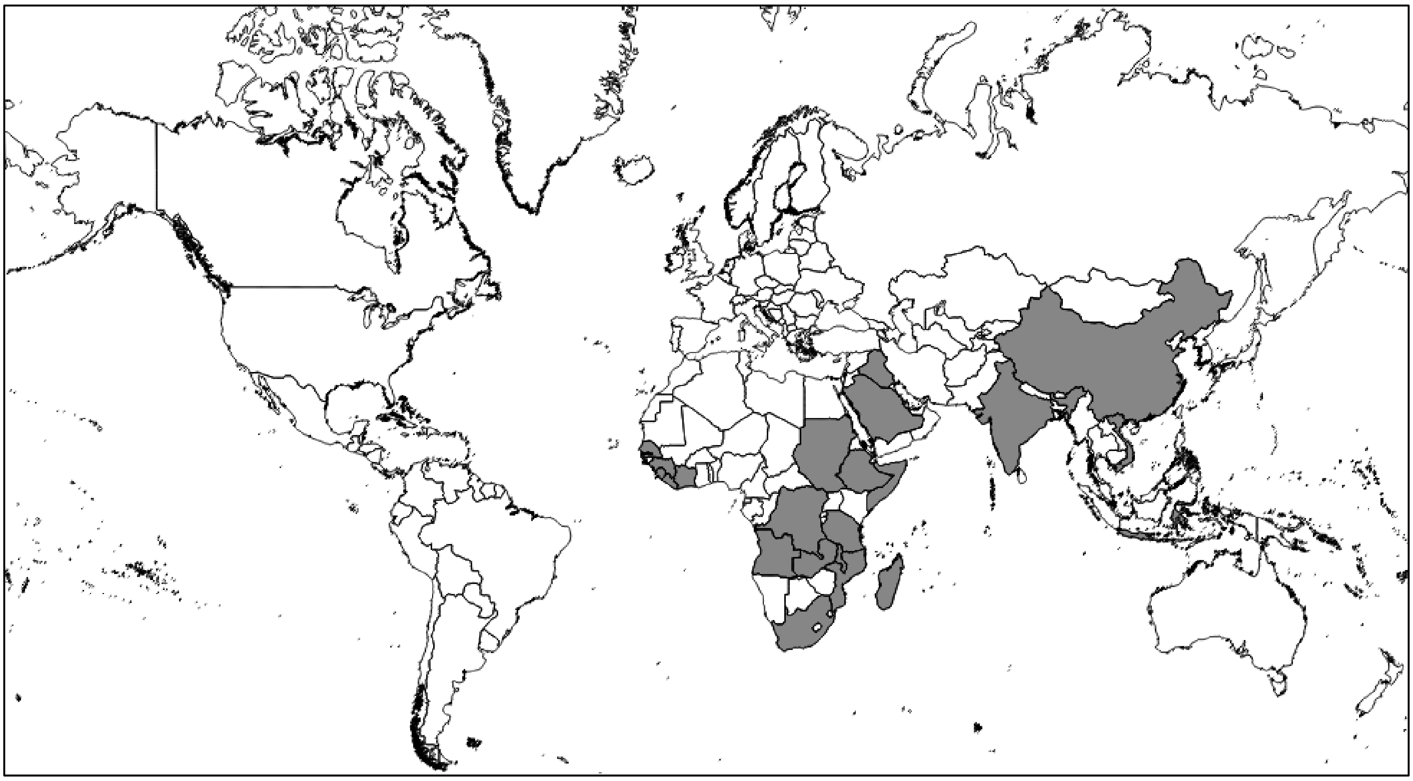

Figure S1.14 Distribution map of the genus *Lestomerus* (Angola, China, Democratic Republic of the Congo, Ethiopia, Gambia, Guinea, India, Indonesia, Iraq, Ivory Coast, Liberia, Madagascar, Malawi, Mozambique, Saudi Arabia, Senegal, Sierra Leone, Somalia, South Africa, Sudan, Tanzania, Vietnam, Zambia)

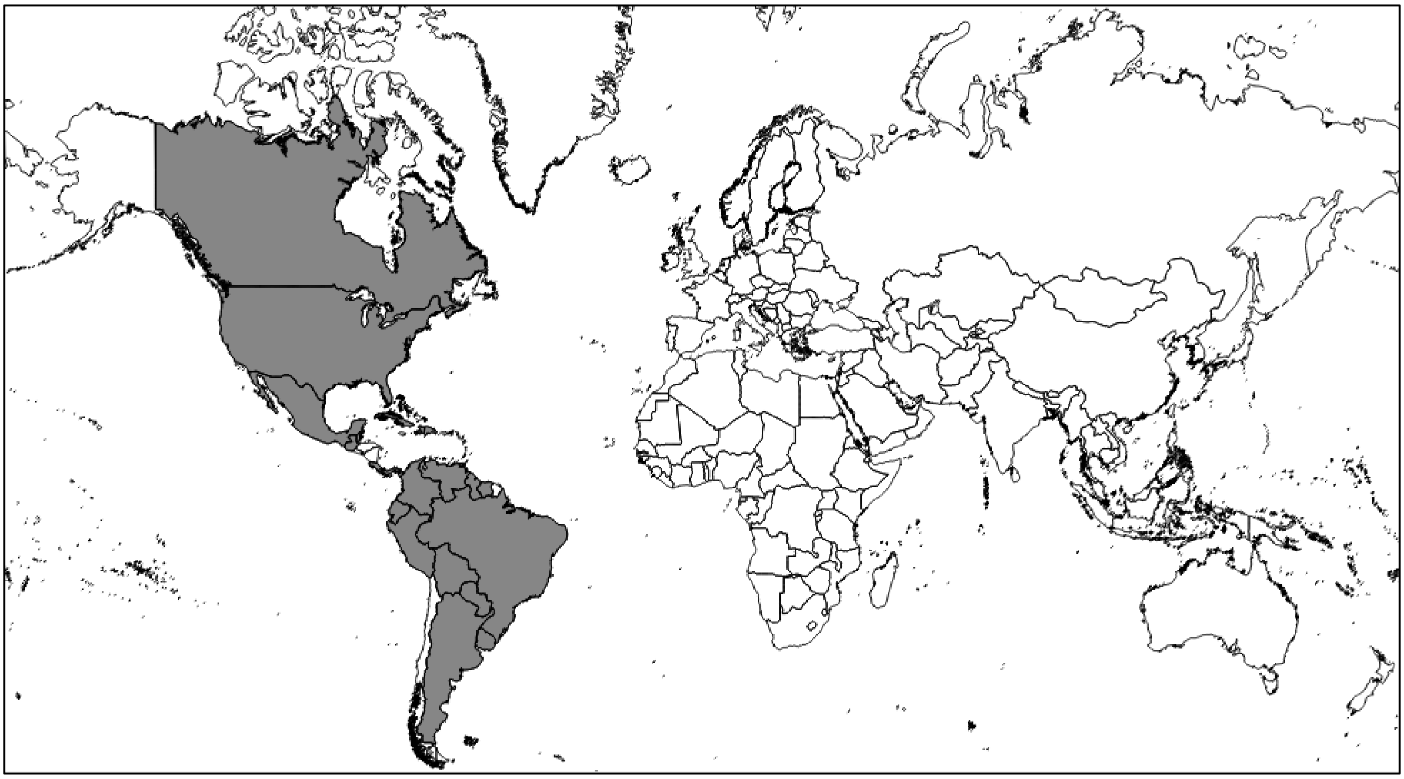

Figure S1.15 Distribution map of the genus *Melanolestes* (Argentina, Belize, Bolivia, Brazil, Canada, Colombia, Costa Rica, Cuba, Dominican Republic, Ecuador, Guatemala, Guyana, Haiti, Mexico, Panama, Paraguay, Peru, Suriname, Trinidad and Tobago, United States, Uruguay, Venezuela)

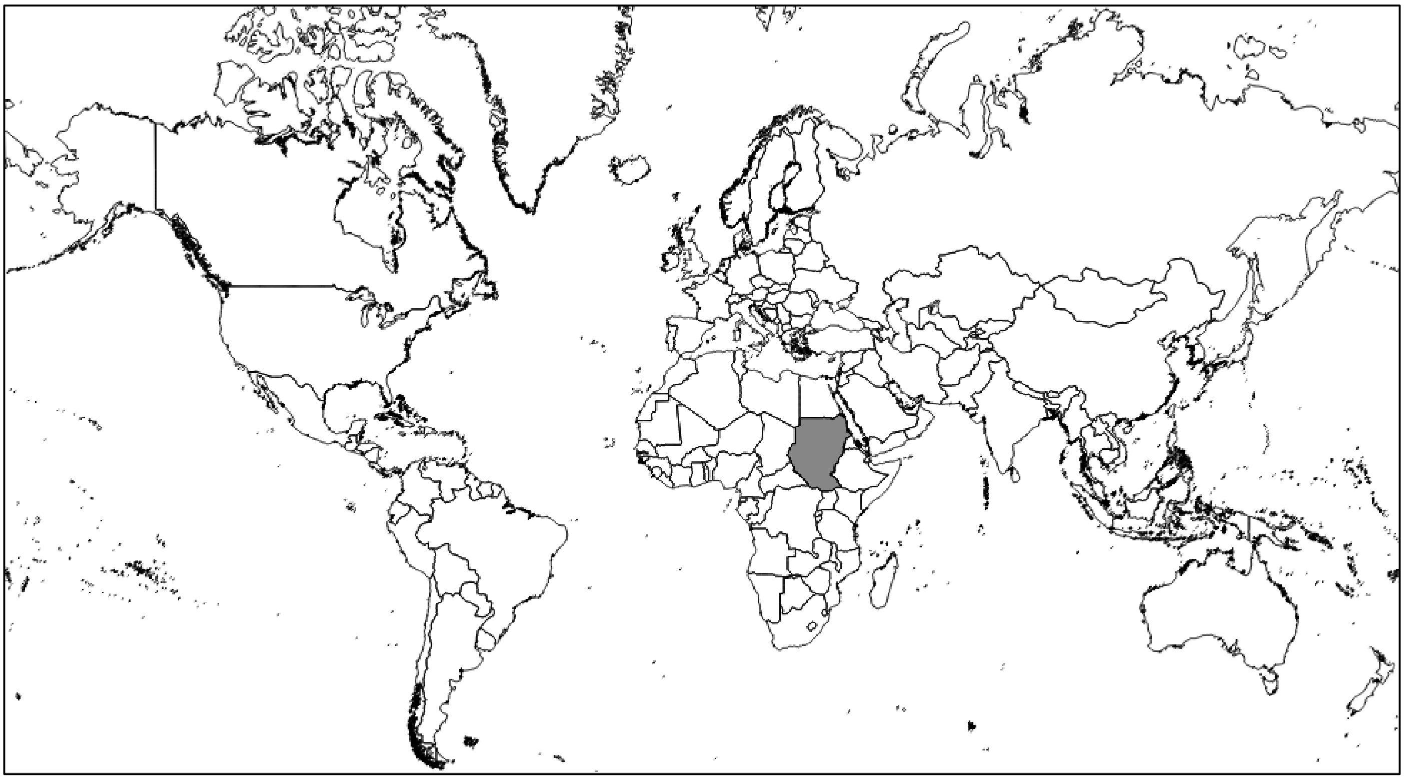

Figure S1.16 Distribution map of the genus *Microcleptocoris* (Sudan)

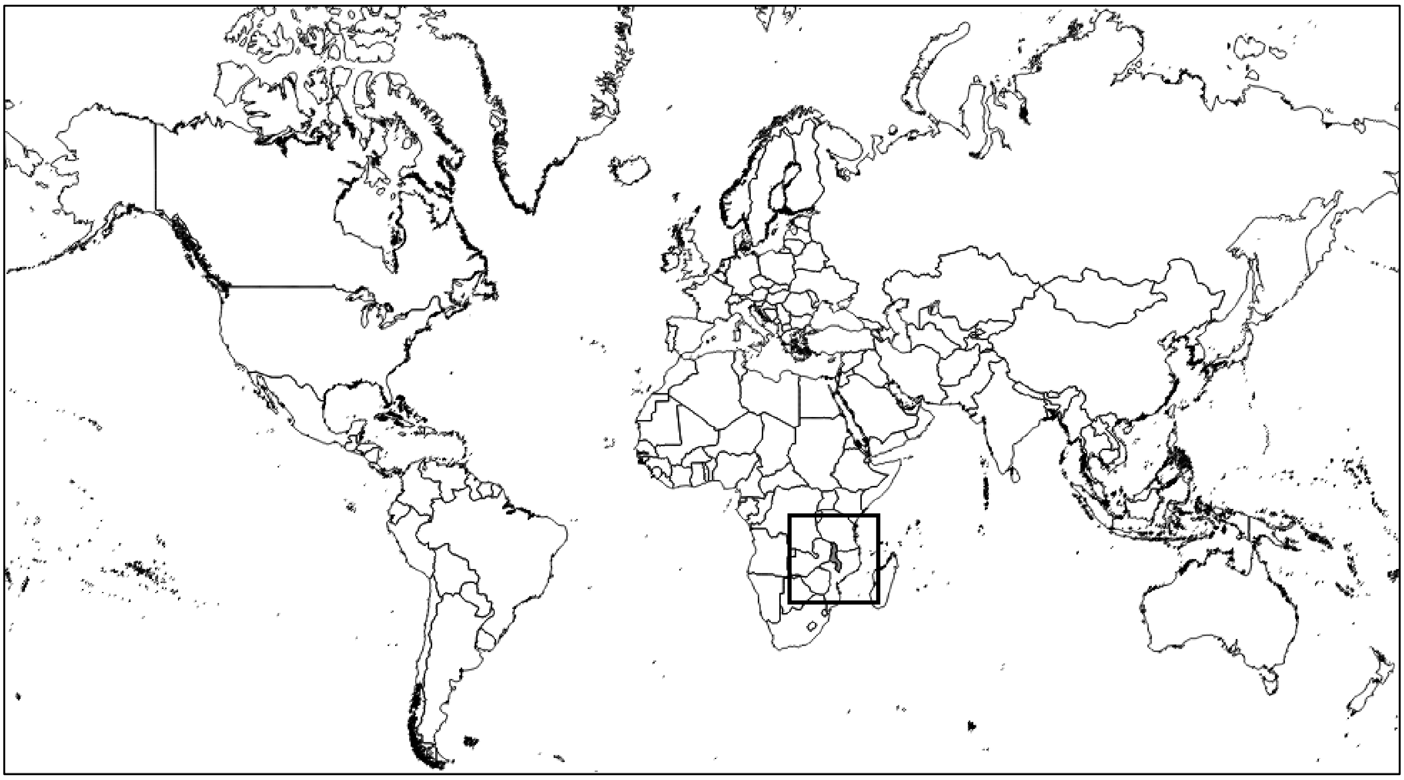

Figure S1.17 Distribution map of the genus *Neopirates* (Malawi)

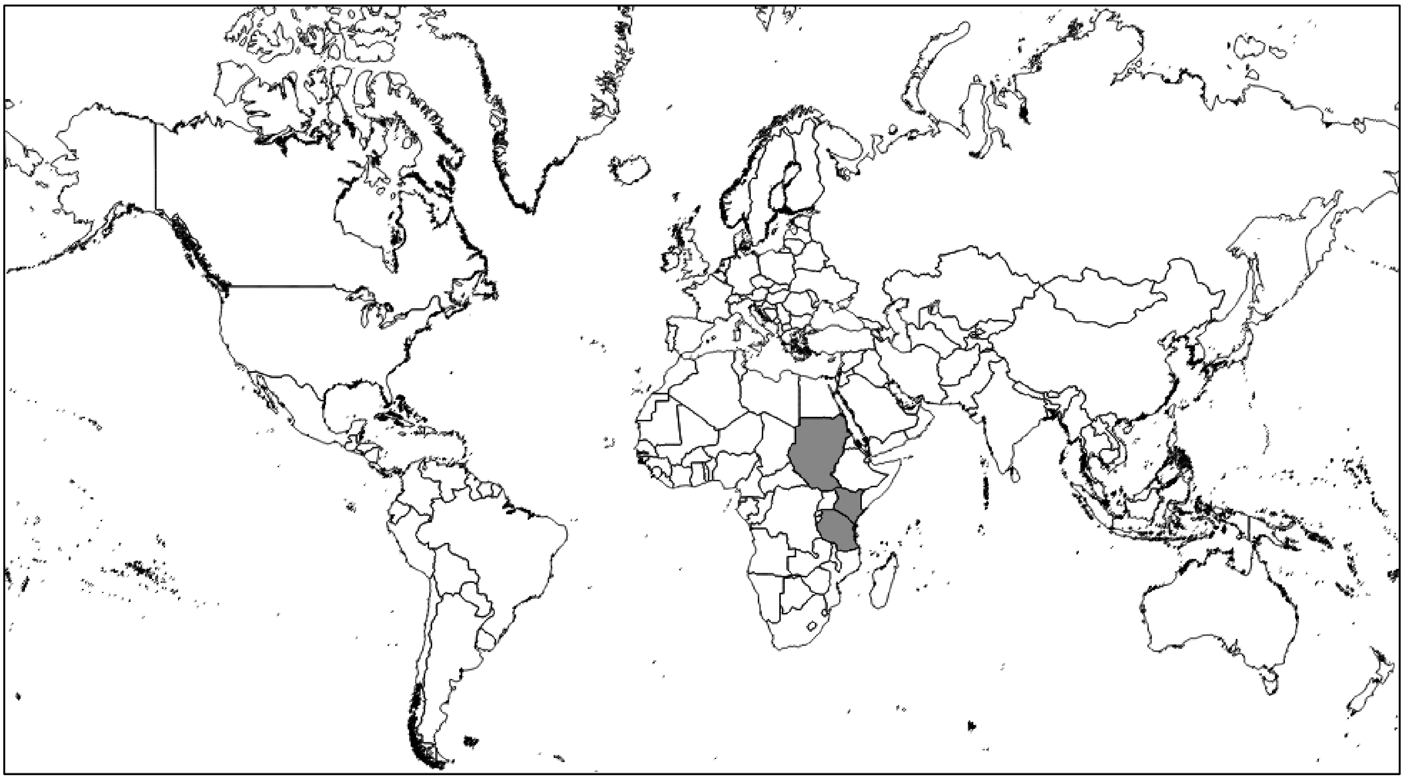

Figure S1.18 Distribution map of the genus *Pachysandalus* (Kenya ,Sudan, Tanzania)

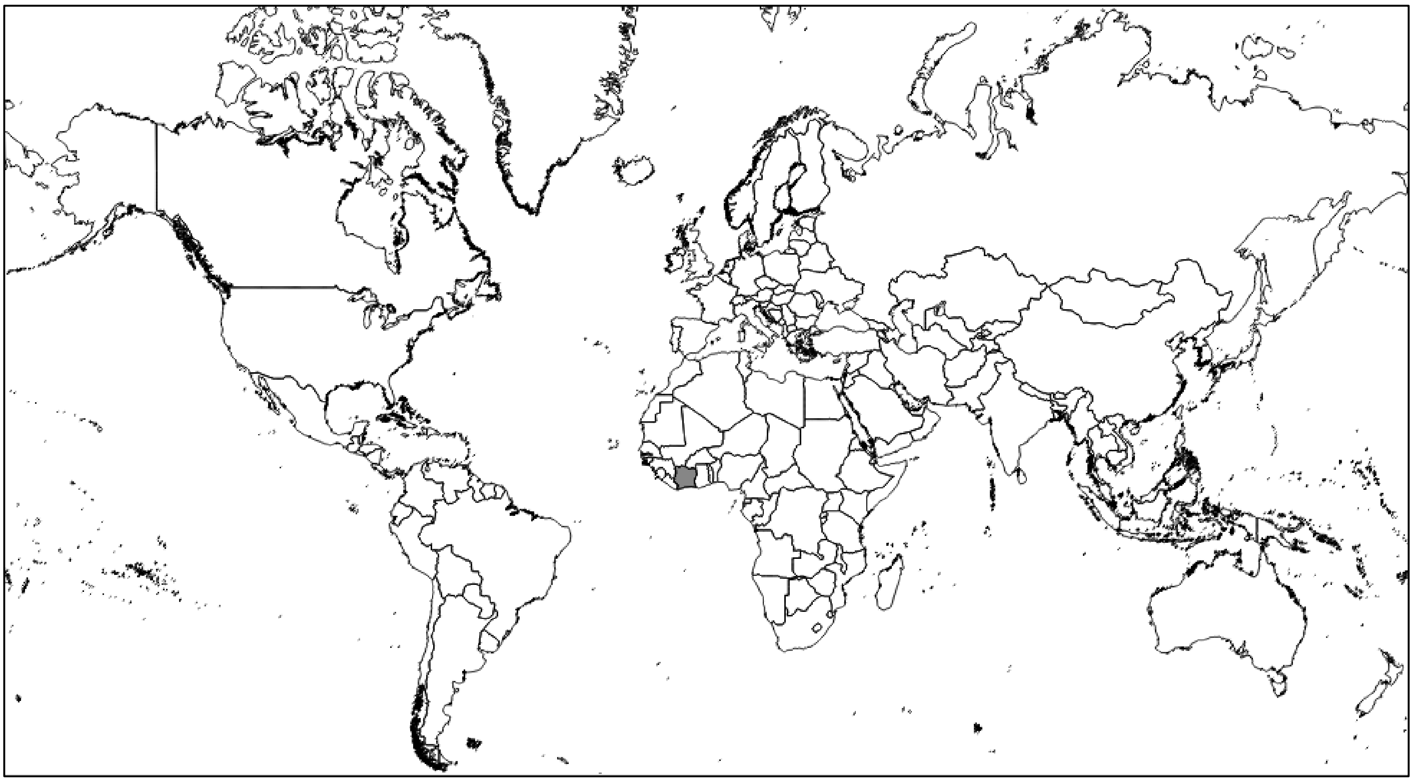

Figure S1.19 Distribution map of the genus *Parapirates* (Ivory Coast)

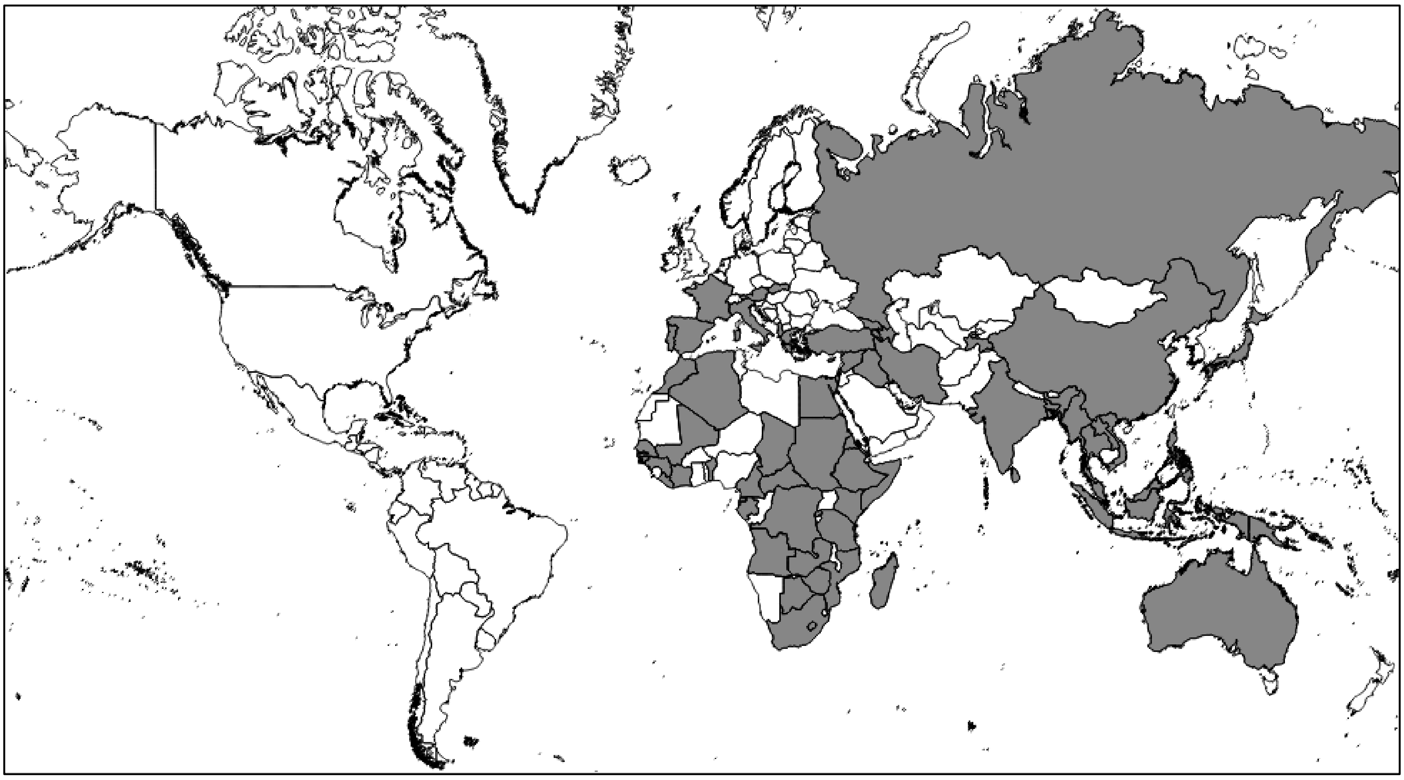

Figure S1.20 Distribution map of the genus *Peirates* (Albania, Algeria, Angola, Armenia, Australia, Austria, Azerbaijan, Bangladesh, Benin, Botswana, Cameroon, Central African Republic, Chad, China, Cyprus, Democratic Republic of the Congo, Egypt, Equatorial Guinea, Eritrea, Ethiopia, France, Gabon, Georgia, Greece, Guinea, Guinea-Bissau, Hong Kong, India, Indonesia, Iran, Iraq, Israel, Italy, Ivory Coast, Japan, Kenya, Laos, Lesotho, Liberia, Madagascar, Malaysia, Mali, Morocco, Mozambique, Myanmar, New Caledonia, Papua New Guinea, Philippines, Portugal, Russia, Rwanda, Senegal, Slovakia, Somalia, South Africa, Spain, Sri Lanka, Sudan, Syria, Tajikistan, Tanzania, Thailand, Turkey, Vietnam, Zambia, Zimbabwe)

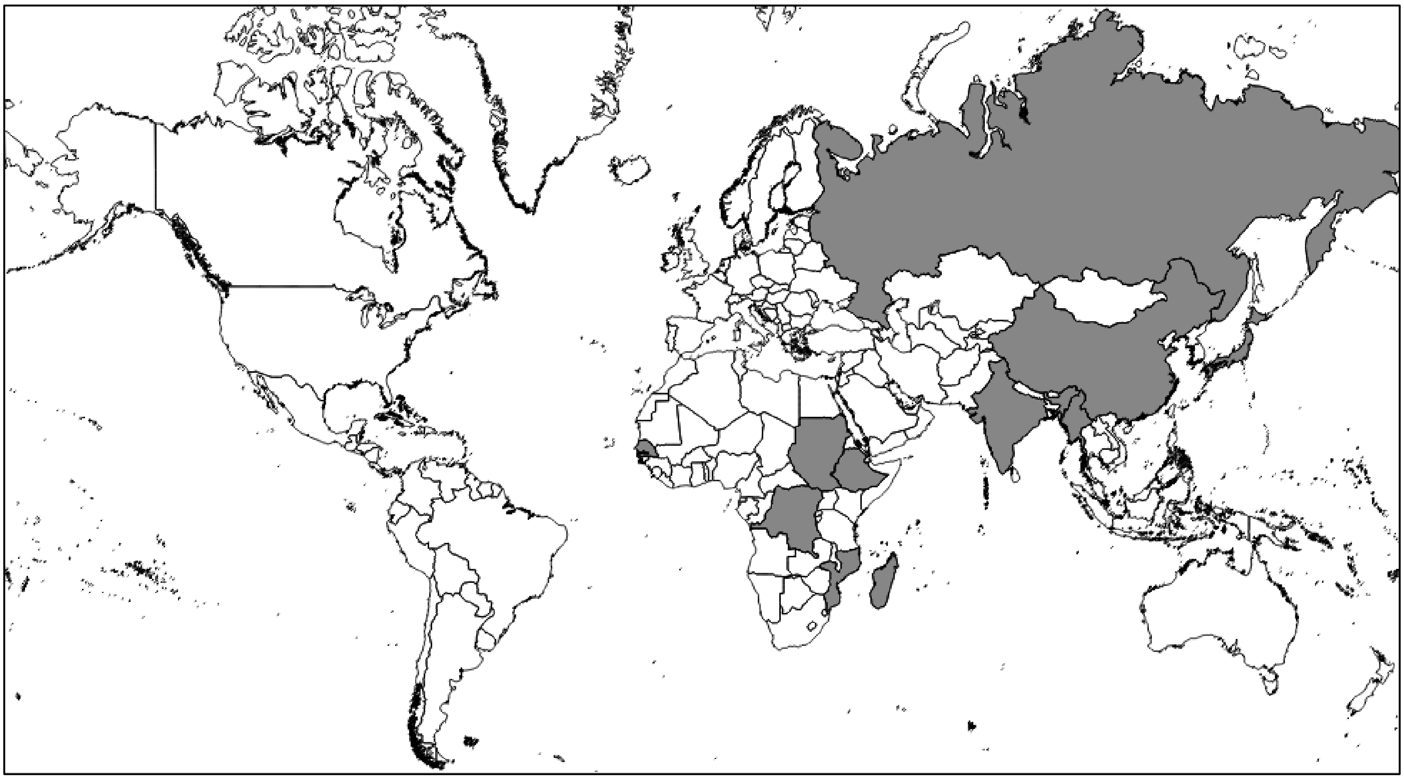

Figure S1.21 Distribution map of the genus *Phalantus* (China, Democratic Republic of the Congo, Ethiopia, Gambia, Hong Kong, India, Japan, Madagascar, Mozambique, Myanmar, Russia, Senegal, Sudan)

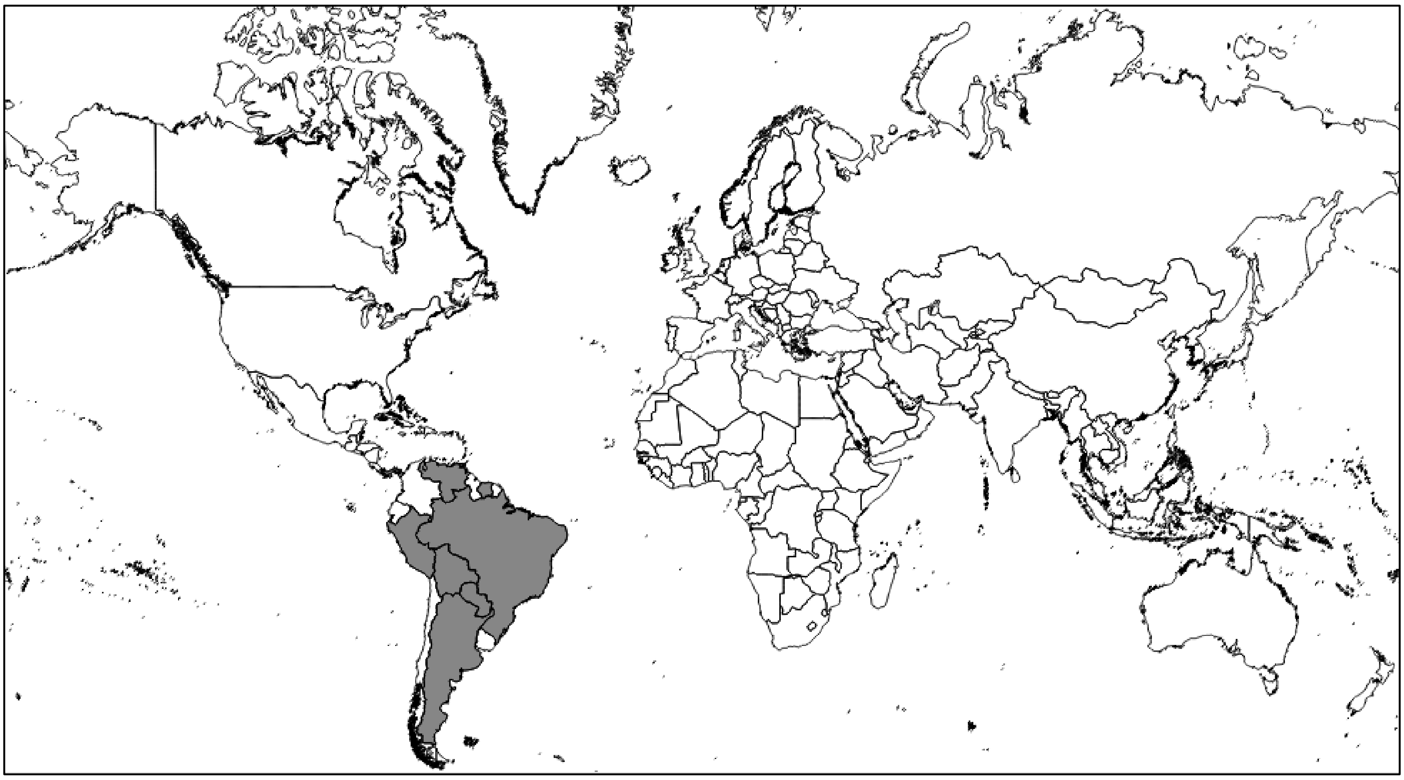

Figure S1.22 Distribution map of the genus *Phorastes* (Argentina, Bolivia, Suriname, Brazil, Venezuela, Peru, Panama, Paraguay, Trinidad and Tobago)

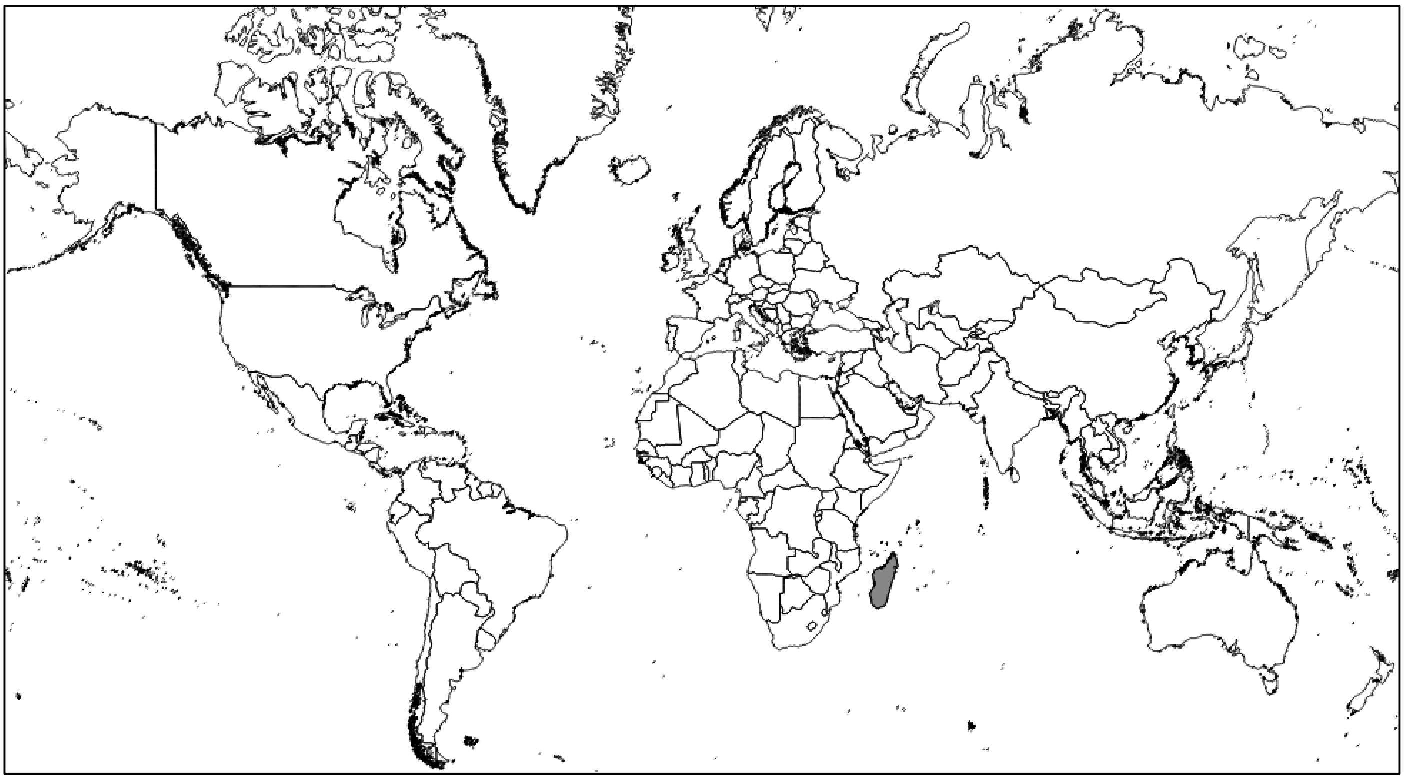

Figure S1.23 Distribution map of the genus *Pseudolestomerus* (Madagascar)

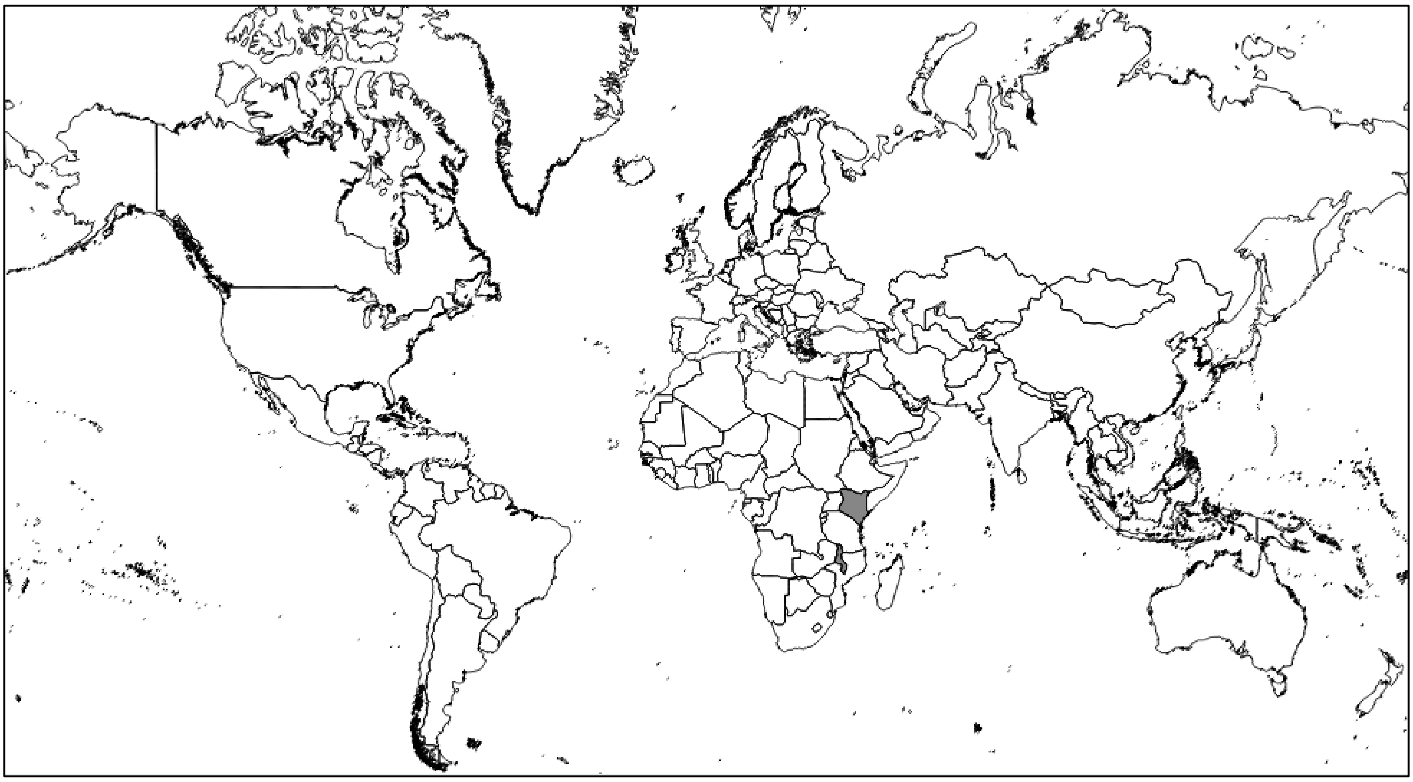

Figure S1.24 Distribution map of the genus *Pteromalestes* (Kenya, Malawi)

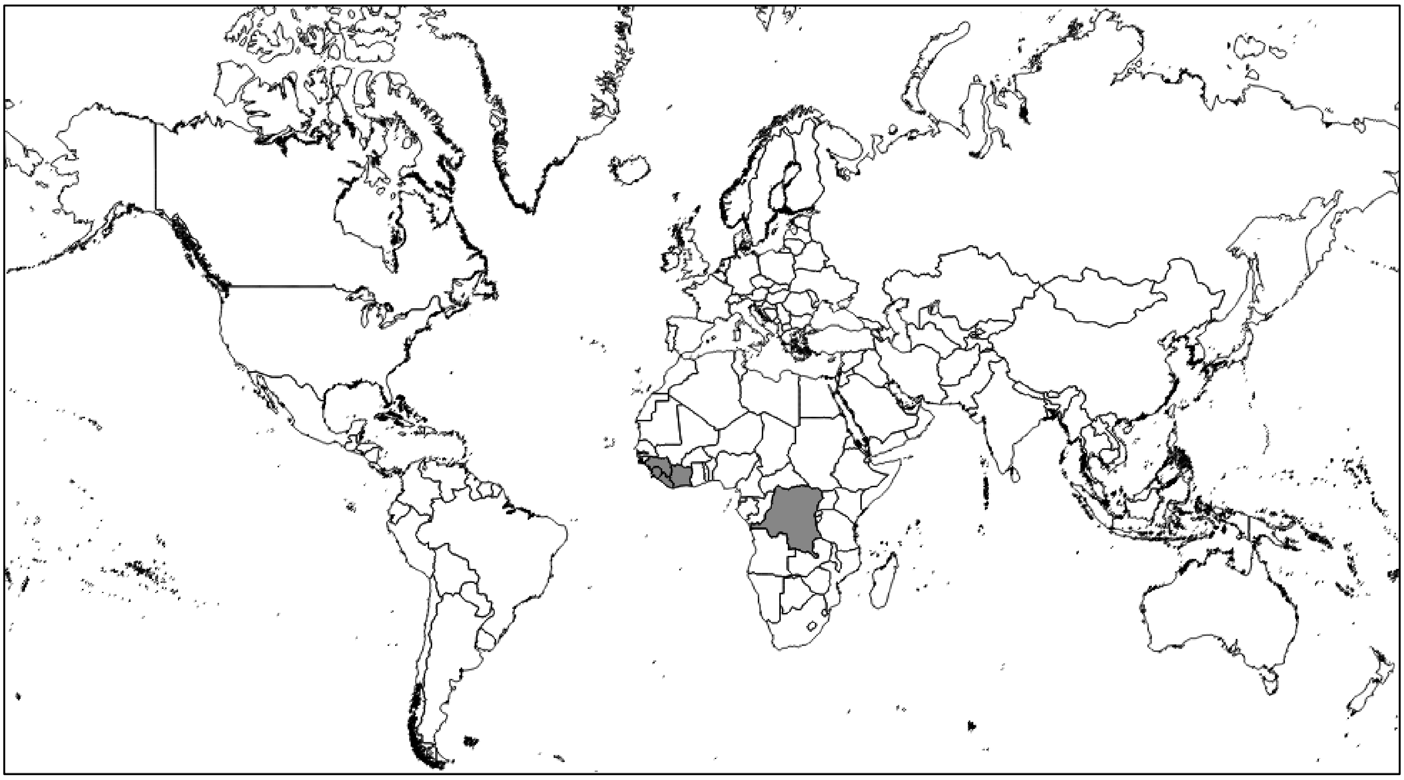

Figure S1.25 Distribution map of the genus *Rapites* (Democratic Republic of the Congo, Guinea, Ivory Coast, Liberia, Sierra Leone)

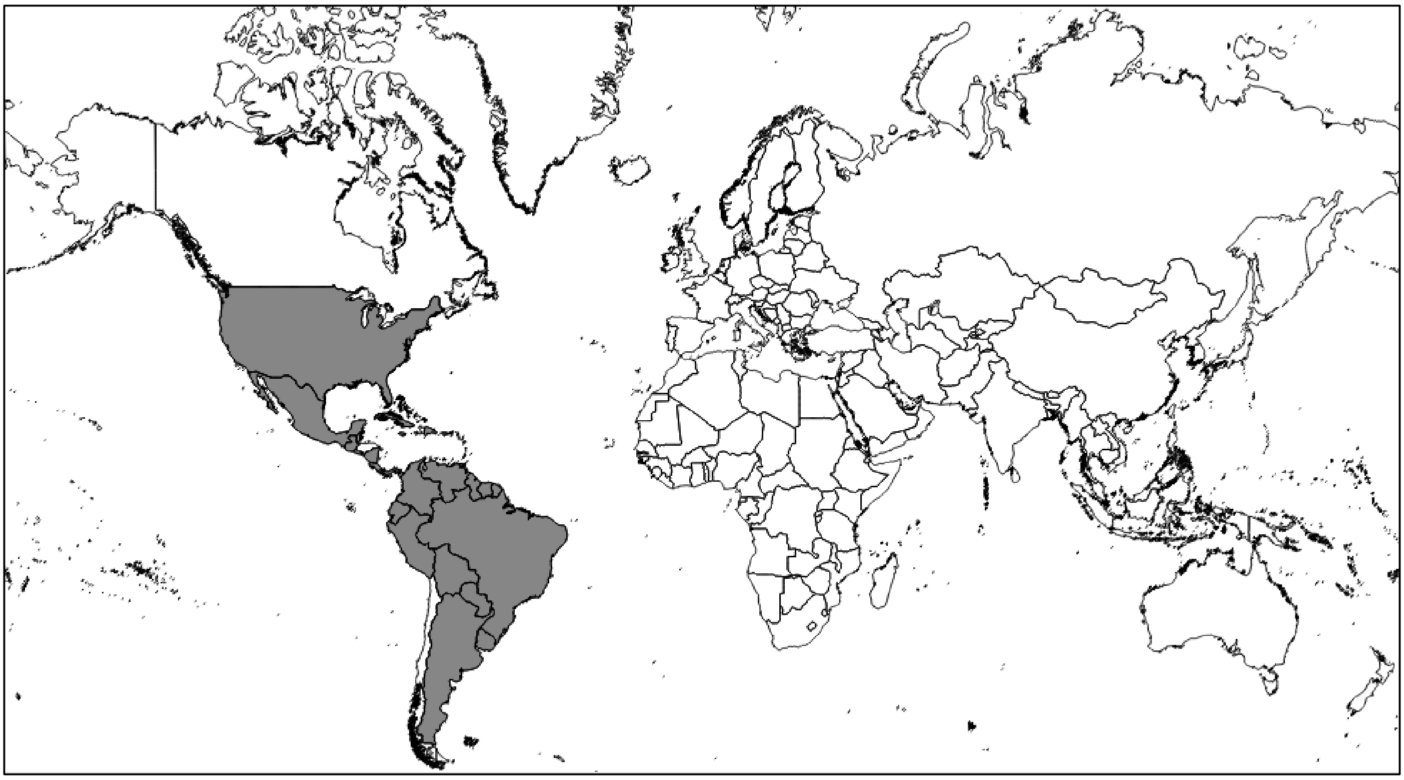

Figure S1.26 Distribution map of the genus *Rasahus* (Argentina, Belize, Bolivia, Brazil, Colombia, Costa Rica, Cuba, Dominican Republic, Ecuador, El Salvador, French Guiana, Guatemala, Guyana, Jamaica, Mexico, Nicaragua, Panama, Paraguay, Peru, Puerto Rico, Suriname, Trinidad and Tobago, United States, Uruguay, Venezuela)

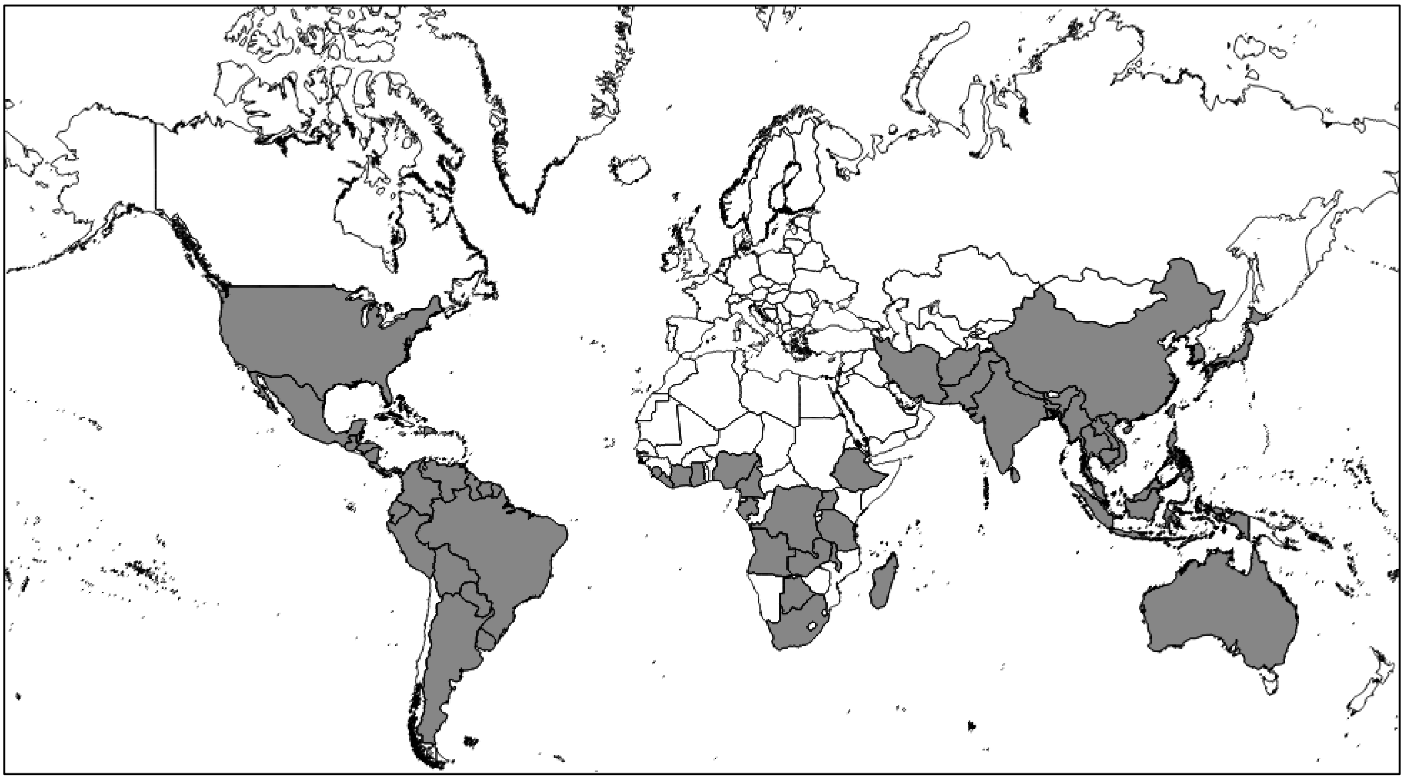

Figure S1.27 Distribution map of the genus *Sirthenea* (Afghanistan, Angola, Argentina, Australia, Bangladesh, Belize, Bolivia, Botswana, Brazil, Cambodia, Cameroon, China, Colombia, Costa Rica, Democratic Republic of the Congo, Dominican Republic, Ecuador, Ethiopia, French Guiana, Gabon, Ghana, Guadeloupe, Guatemala, Guyana, Honduras, Hong Kong, India, Indonesia, Iran, Ivory Coast, Japan, Laos, Liberia, Madagascar, Malawi, Malaysia, Martinique, Mexico, Myanmar, Nepal, Nicaragua, Nigeria, Pakistan, Panama, Paraguay, Peru, Philippines, Saint Lucia, Sierra Leone, South Africa, South Korea, Sri Lanka, Suriname, Taiwan, Tanzania, Thailand, Trinidad and Tobago, Uganda, United States, Uruguay, Venezuela, Vietnam, Zambia)

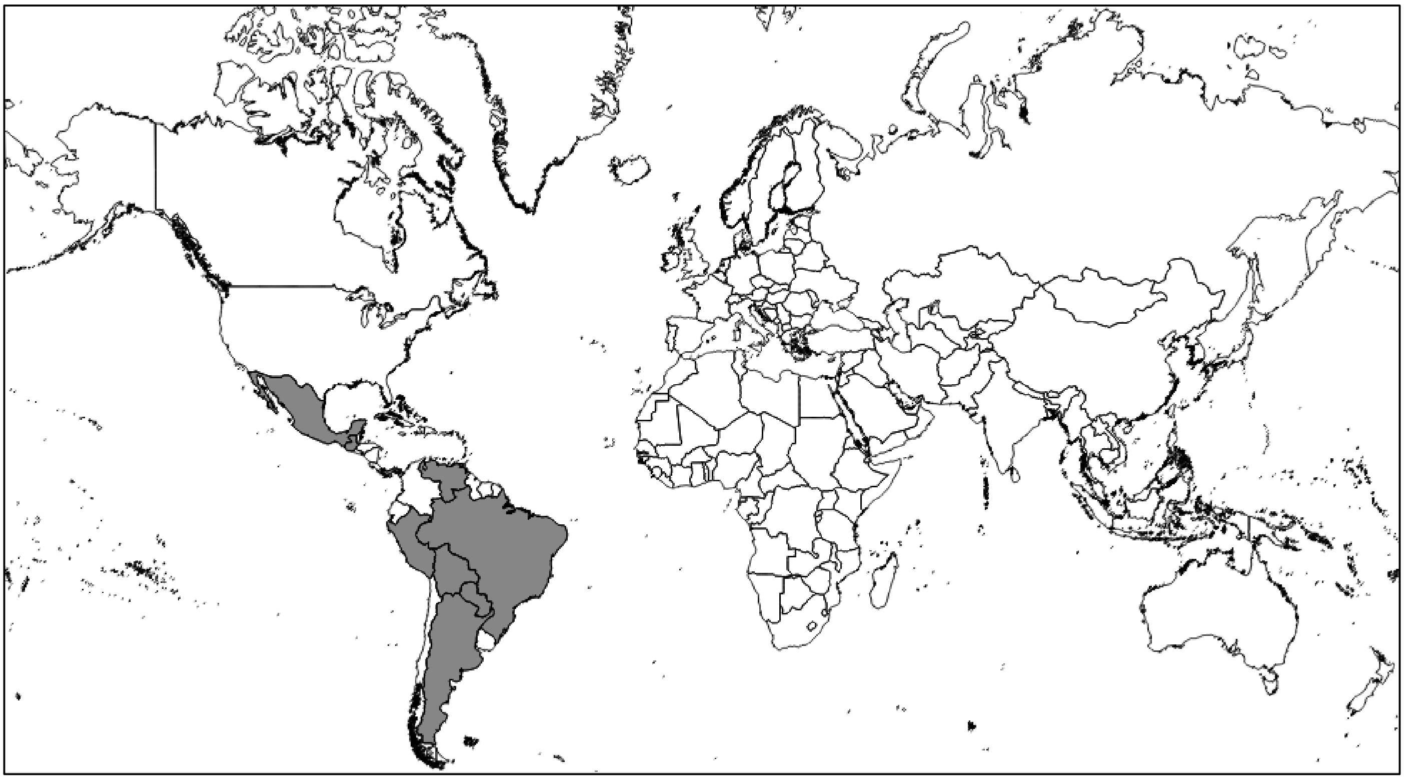

Figure S1.28 Distribution map of the genus *Thymbreus* (Argentina, Bolivia, Brazil, Guatemala, Mexico, Panama, Paraguay, Peru, Venezuela)

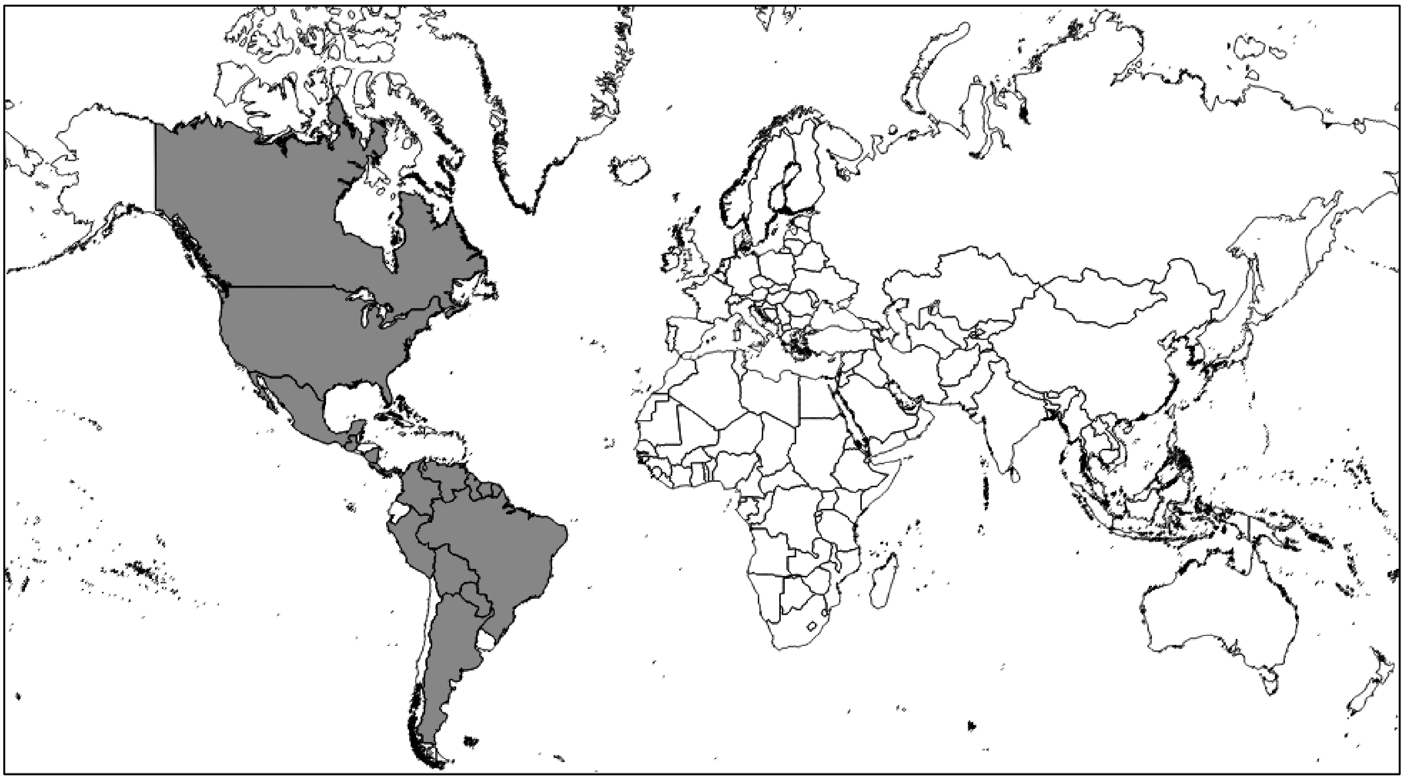

Figure S1.29 Distribution map of the genus *Tyfides* (Argentina, Bolivia, Brazil, Canada, Colombia, Costa Rica, French Guiana, Grenada, Guatemala, Guyana, Mexico, Nicaragua, Panama, Paraguay, Peru, Suriname, Trinidad and Tobago, United States, Venezuela)

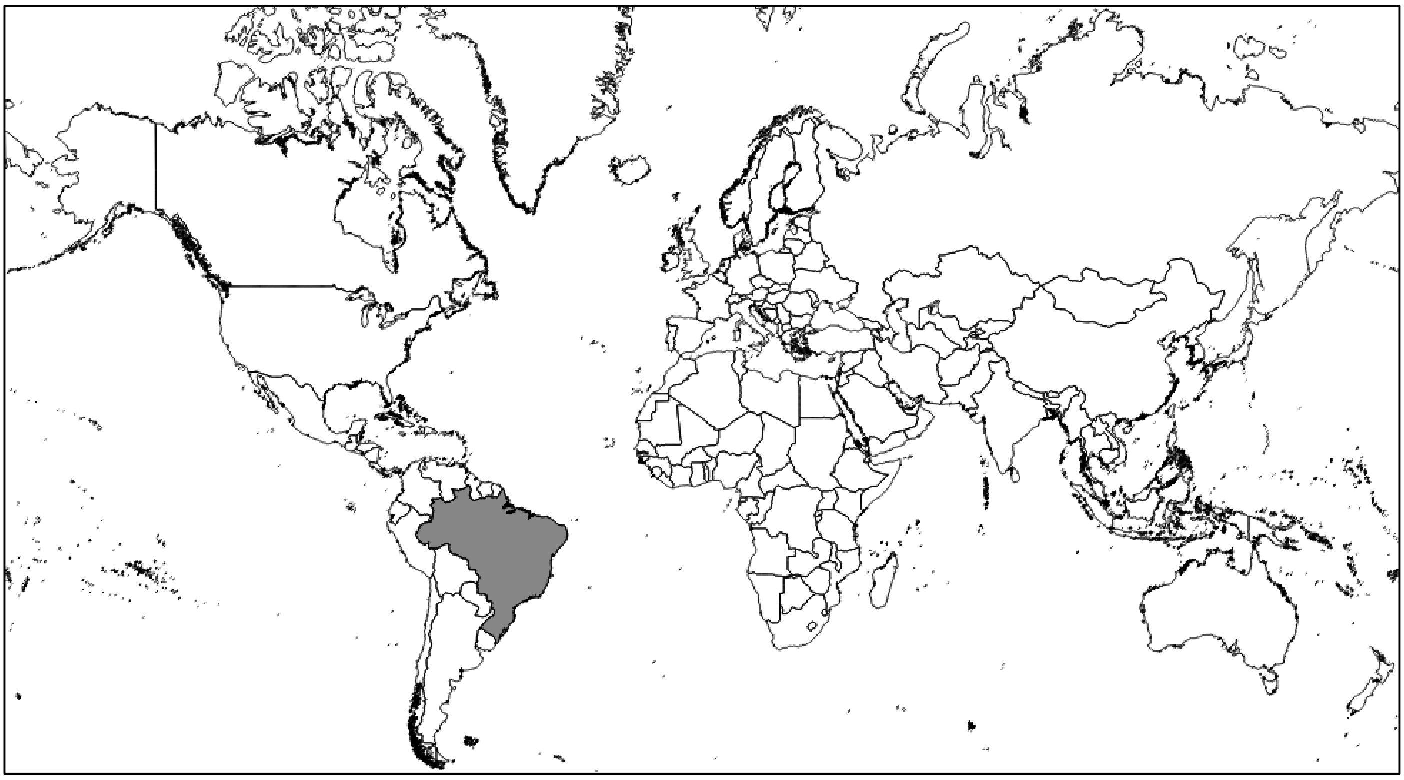

Figure S1.30 Distribution map of the genus *Zeikiria* (Brazil)

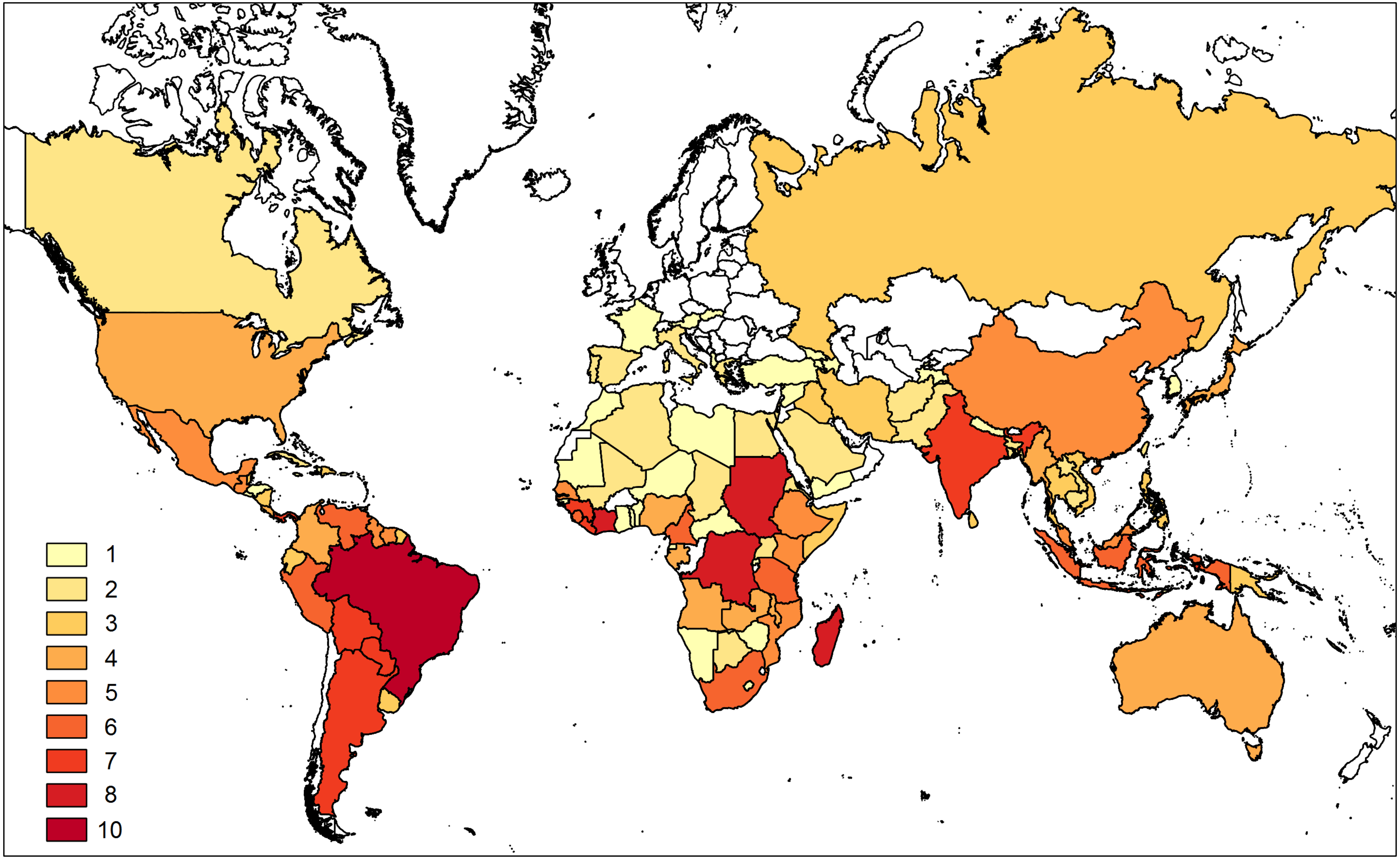

Figure S1.31 Map of the World showing the number of Peiratinae genera occurring in each country
