# Supplementary material for: Distribution Pattern and Climate Preferences of the Representatives of the Cosmopolitan Genus Sirthenea Spinola, 1840 (Heteroptera: Reduviidae: Peiratinae)
Source: PLoS One. 2015 Oct 23;10(10):e0140801. doi: 10.1371/journal.pone.0140801 (PMC4619806; doi:10.1371/journal.pone.0140801)
Supplement: S3 Appendix — (PDF) [file pone.0140801.s003.pdf]

Supporting Information

Distribution pattern and climate preferences of the representatives of the genus *Sirthenea* Spinola, 1840 (Heteroptera: Reduviidae: Peiratinae)

PLOS ONE

Dominik Chłond*, Agnieszka Bugaj-Nawrocka

Department of Zoology, Faculty of Biology and Environmental Protection, University of Silesia, Katowice, Poland

* Correspondence: Dominik Chłond, Department of Zoology, Faculty of Biology and Environmental Protection, University of Silesia, Bankowa 9, 40-007 Katowice, Poland.

e-mail: dominik.chlond@us.edu.pl; abugaj-nawrocka@us.edu.pl

Supporting Information S3: Maxent model outputs and climatic diagrams

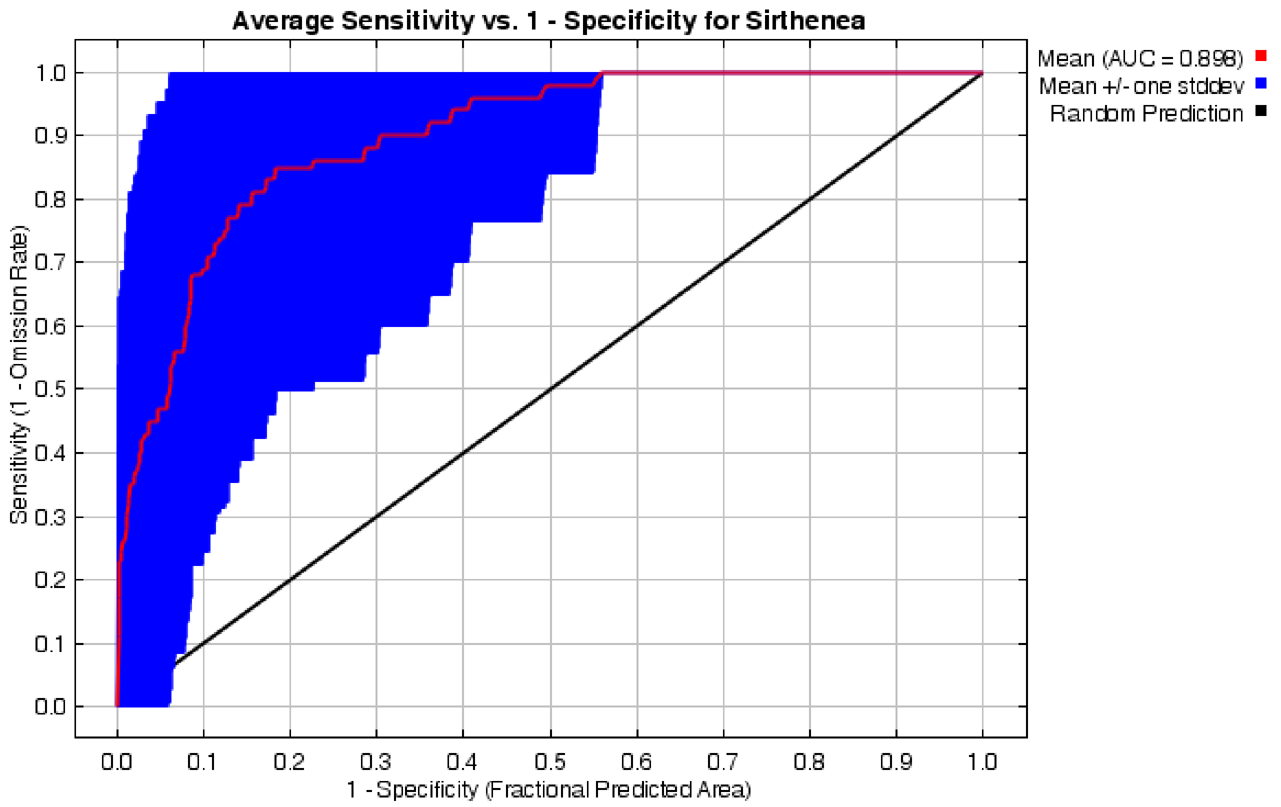

Figure S3.1. The receiver operating characteristic (ROC) curve generated in Maxent, showing an average of 50 repetitions of the model for representatives of the genus *Sirthenea* in Africa.

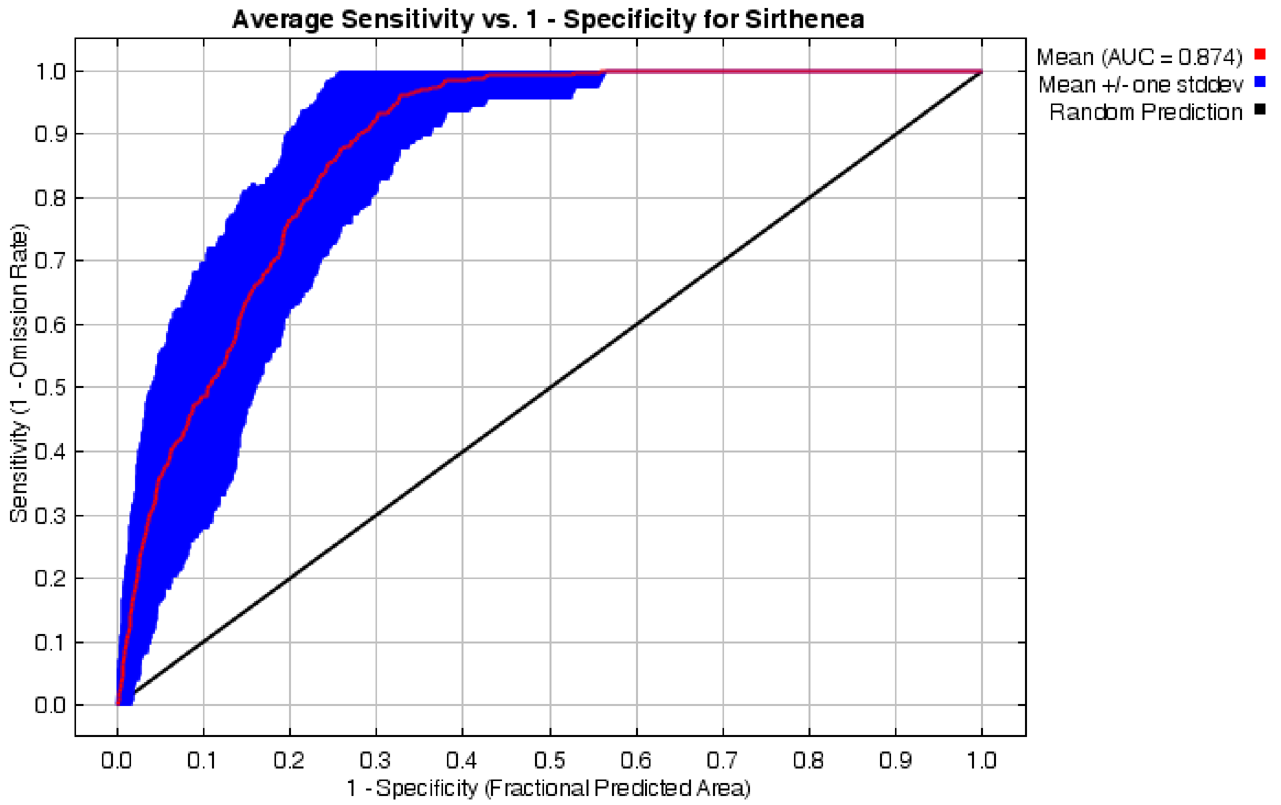

Figure S3.2. The receiver operating characteristic (ROC) curve generated in Maxent, showing an average of 50 repetitions of the model for representatives of the genus *Sirthenea* in Americas.

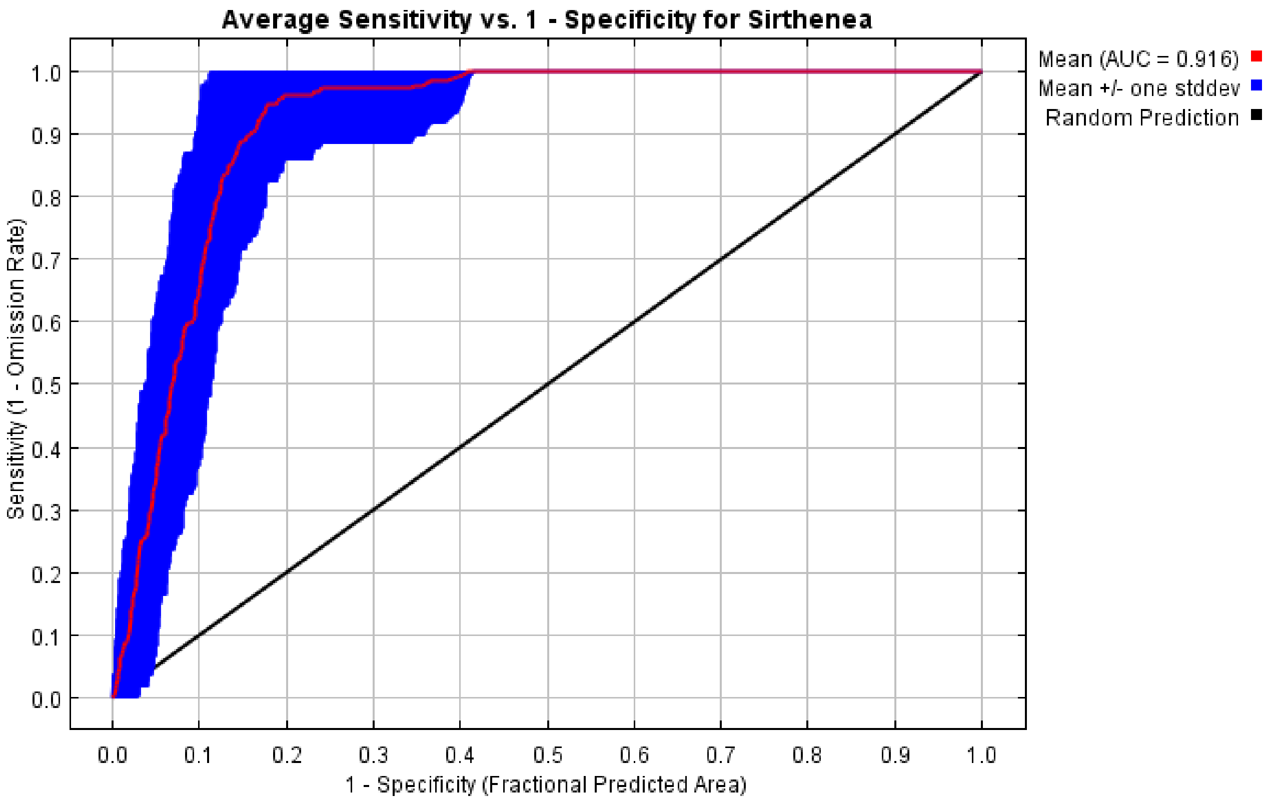

Figure S3.3. The receiver operating characteristic (ROC) curve generated in Maxent, showing an average of 50 repetitions of the model for representatives of the genus *Sirthenea* in Asia and Australia.

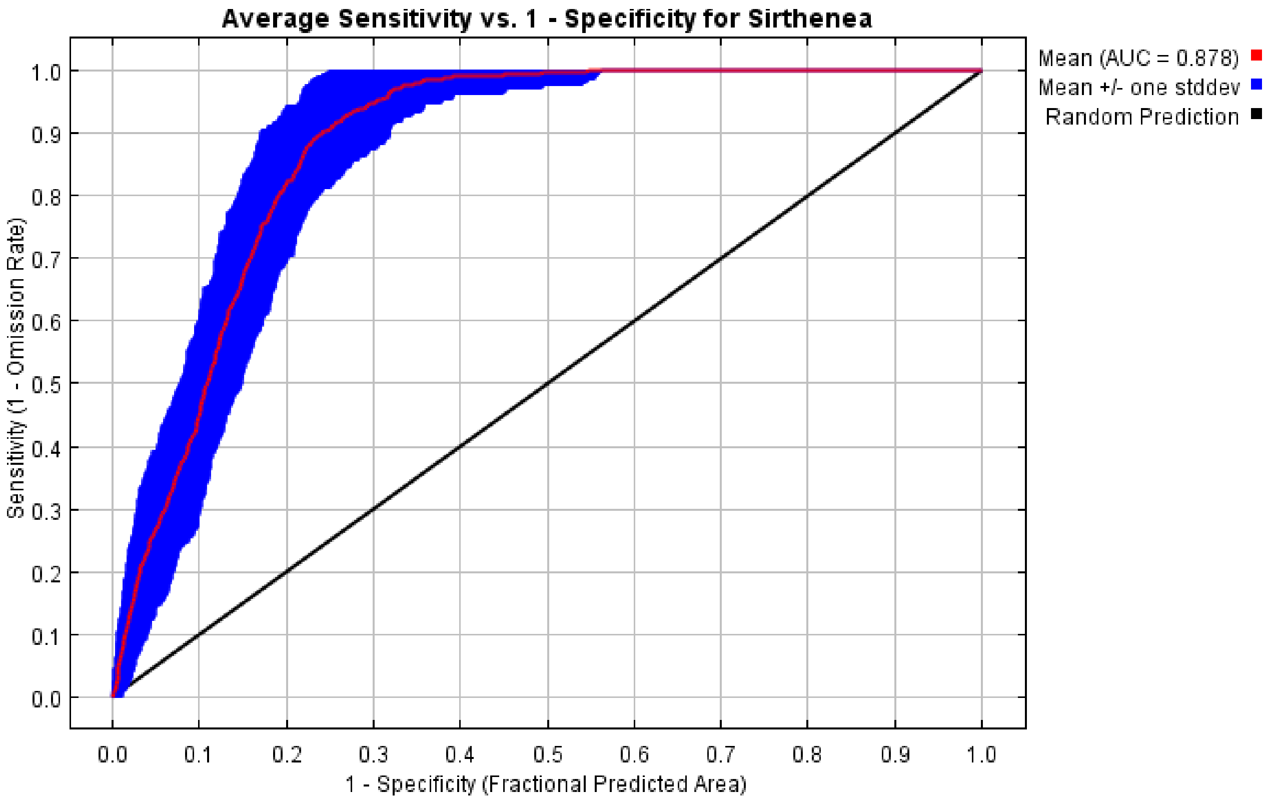

Figure S3.4. The receiver operating characteristic (ROC) curve generated in Maxent, showing an average of 50 repetitions of the model for representatives of the genus *Sirthenea* around the world.

(a) Africa

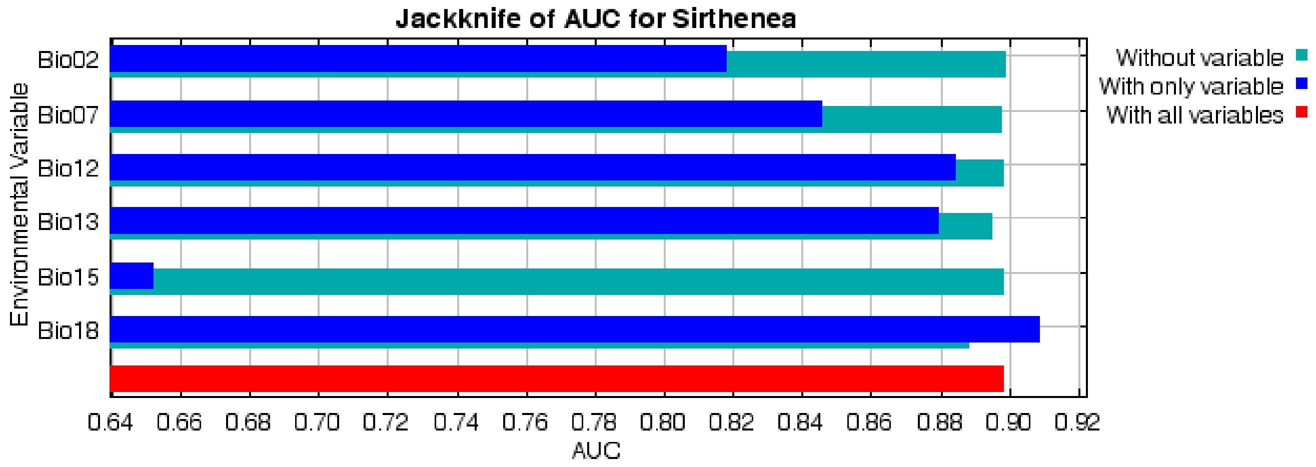

(b) Americas

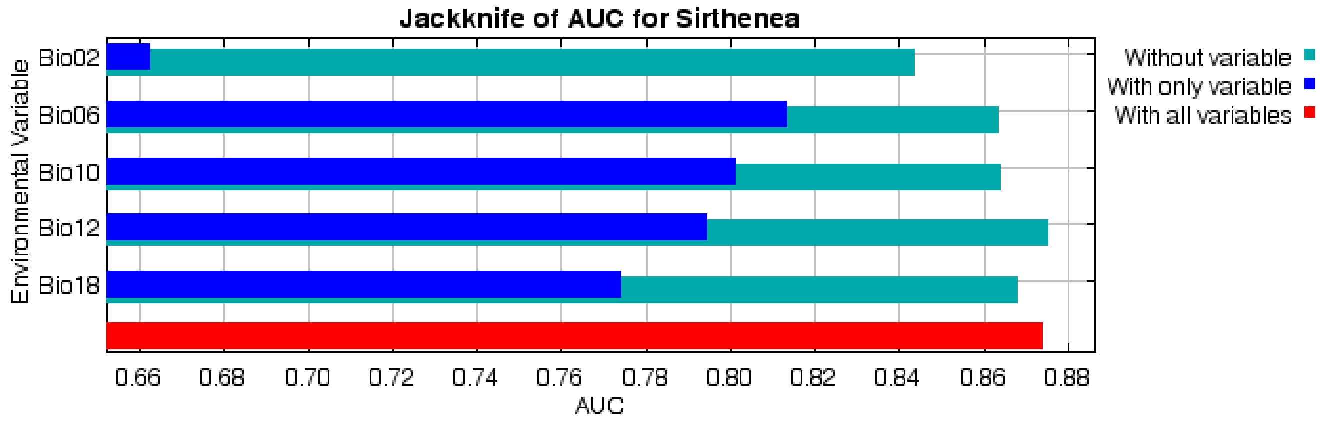

(c) Asia and Australia

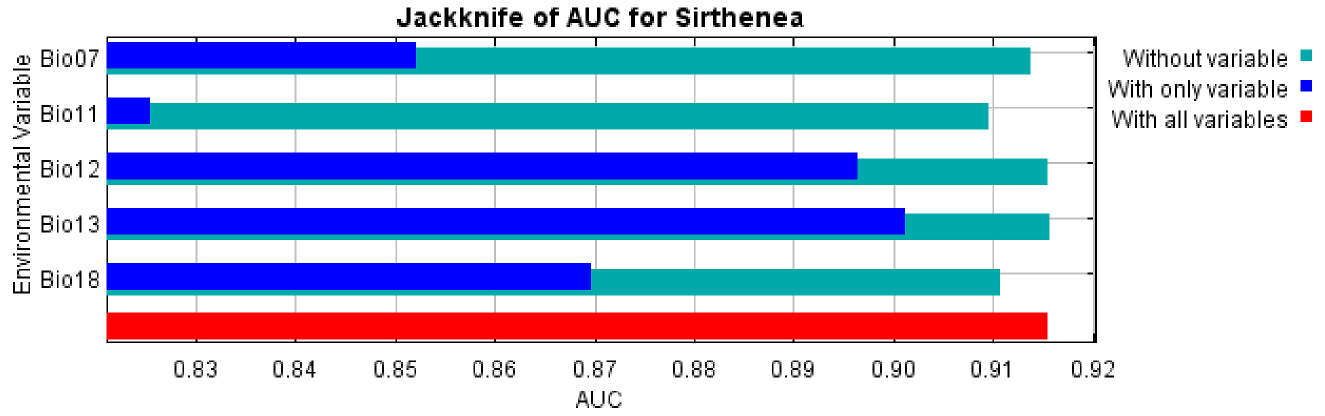

(d) the whole world

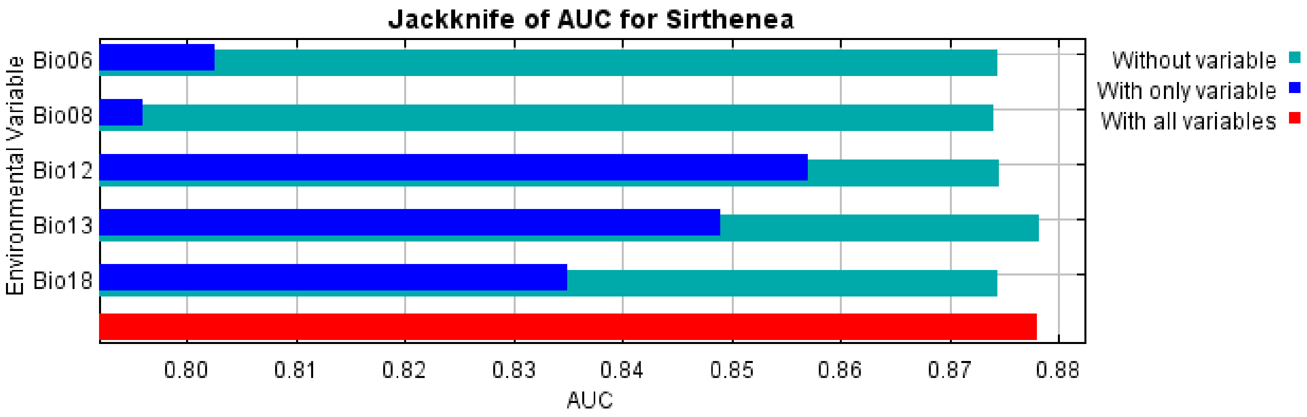

Figure S3.5. Results of jackknife test of variable importance using AUC on test data for (a) Africa, (b) Americas, (c) Asia and Australia, and (d) the whole world. The jackknife test in blue bars shows individual environmental variable importance relative to the red bar which shows all environmental variables. Light blue bar shows whether a variable has any information that isn't present in the other variables, and a dark blue bar shows whether a variable has any useful information by itself. Values shown are averages over replicate runs.

(a) Africa

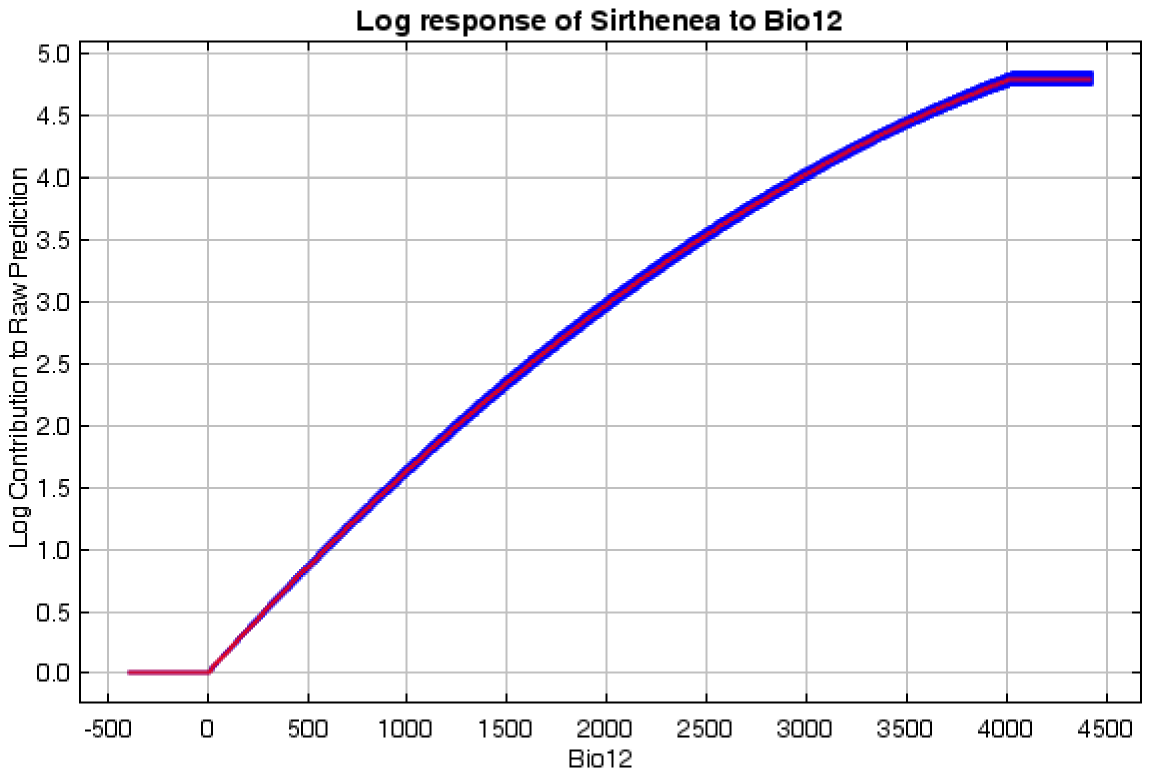

Log response of Sirthenea to Bio12
Log Contribution to Raw Prediction
5.0
4.5
4.0
3.5
3.0
2.5
2.0
1.5
1.0
0.5
0.0
-500
0
500
1000
1500
2000
2500
3000
3500
4000
4500
Bio12

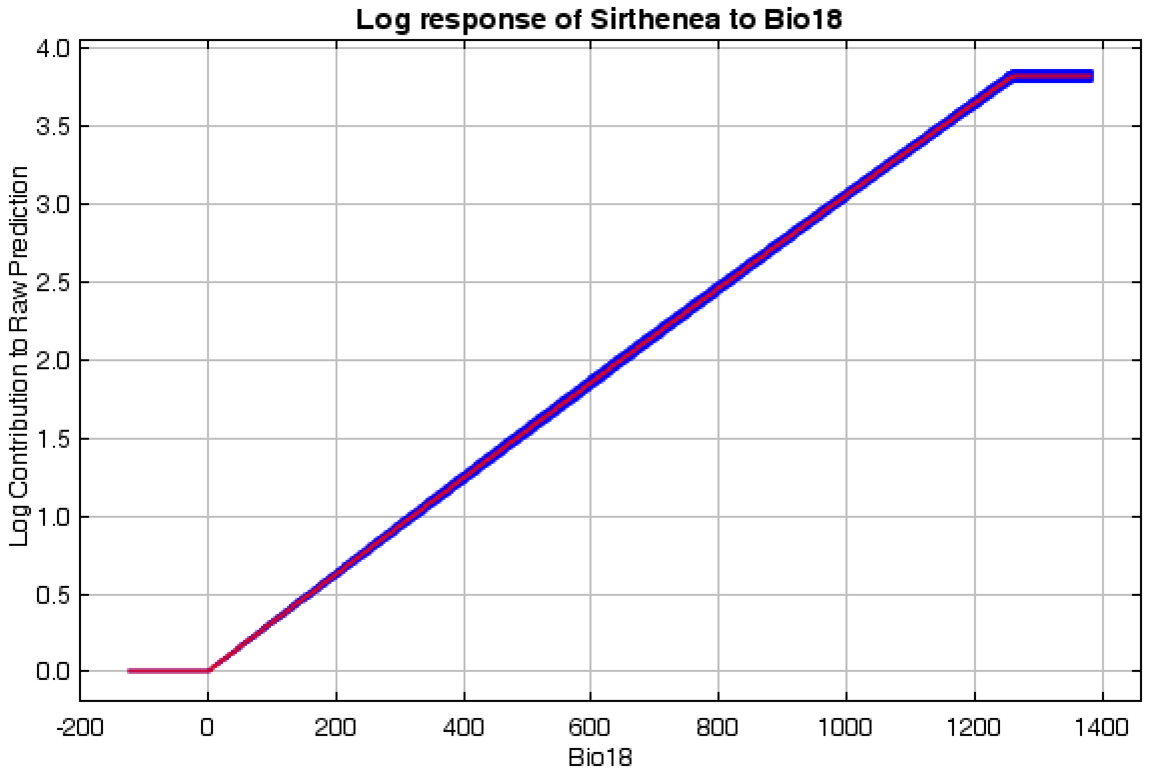

Log response of Sirthenea to Bio18
Log Contribution to Raw Prediction
4.0
3.5
3.0
2.5
2.0
1.5
1.0
0.5
0.0
-200
0
200
400
600
800
1000
1200
1400
Bio18

(b) Americas

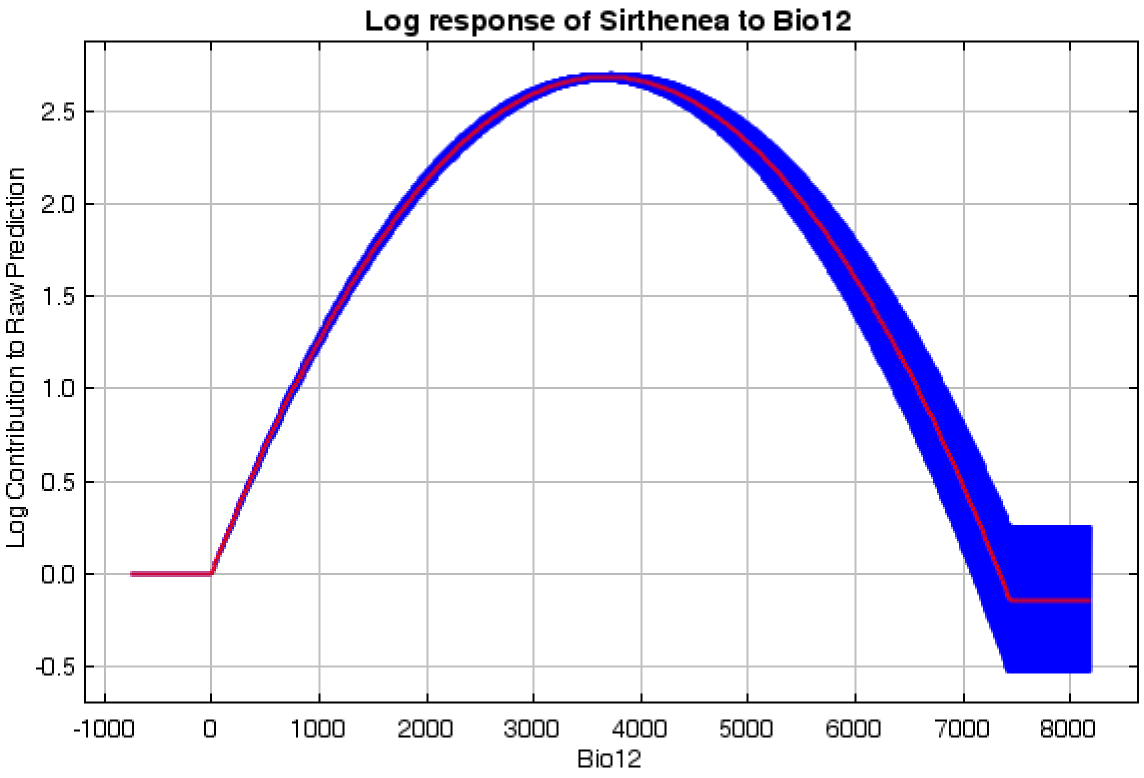
Log response of Sirthenea to Bio12
Log Contribution to Raw Prediction
2.5
2.0
1.5
1.0
0.5
0.0
-0.5
-1000
0
1000
2000
3000
4000
5000
6000
7000
8000
Bio12

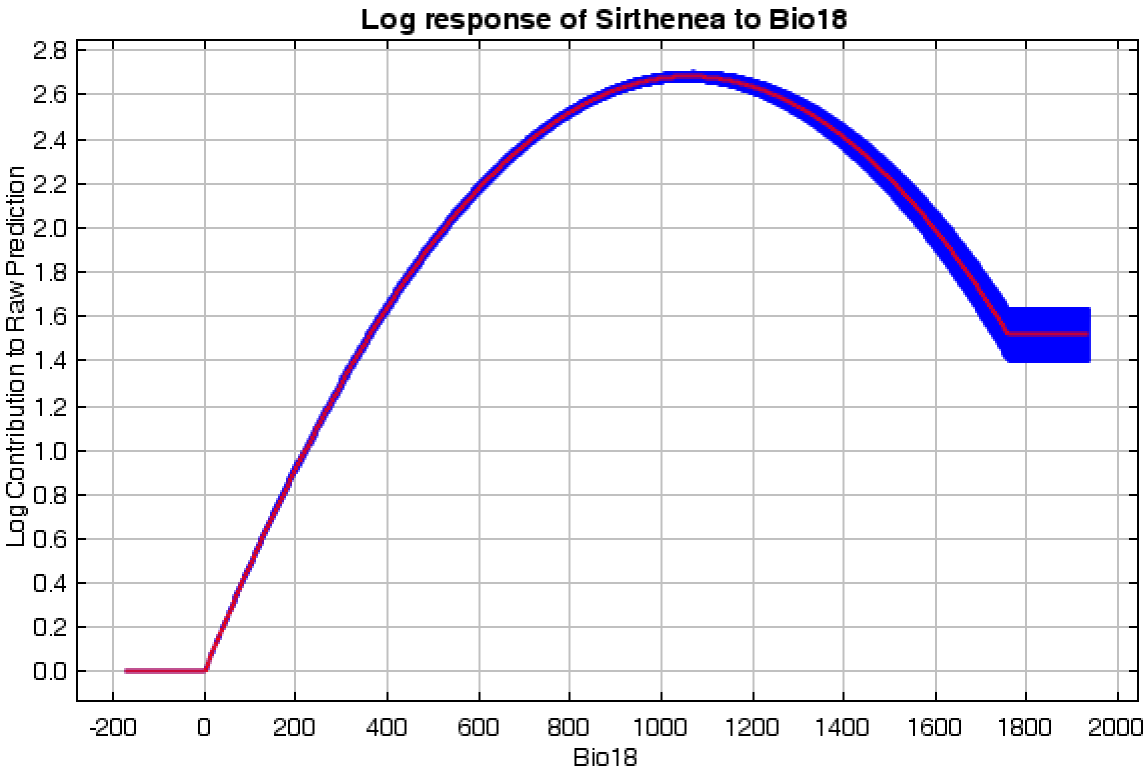
Log response of Sirthenea to Bio18
Log Contribution to Raw Prediction
2.8
2.6
2.4
2.2
2.0
1.8
1.6
1.4
1.2
1.0
0.8
0.6
0.4
0.2
0.0
-200
0
200
400
600
800
1000
1200
1400
1600
1800
2000
Bio18

(c) Asia and Australia

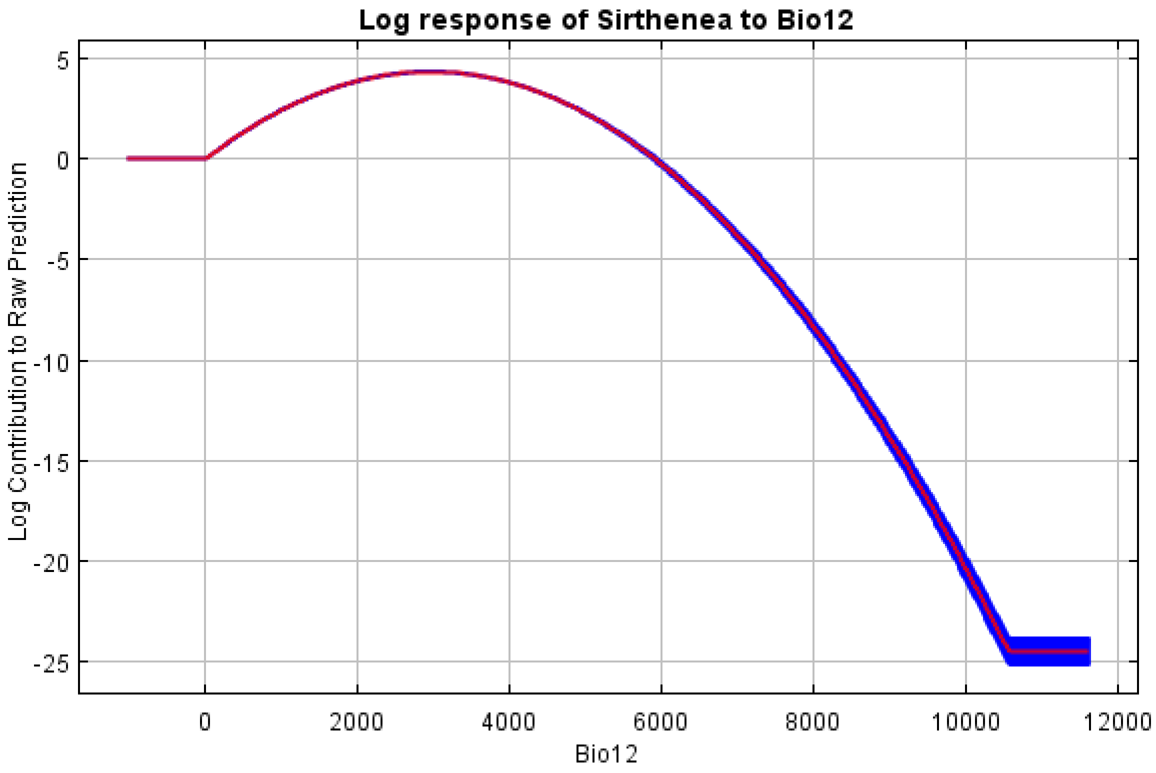

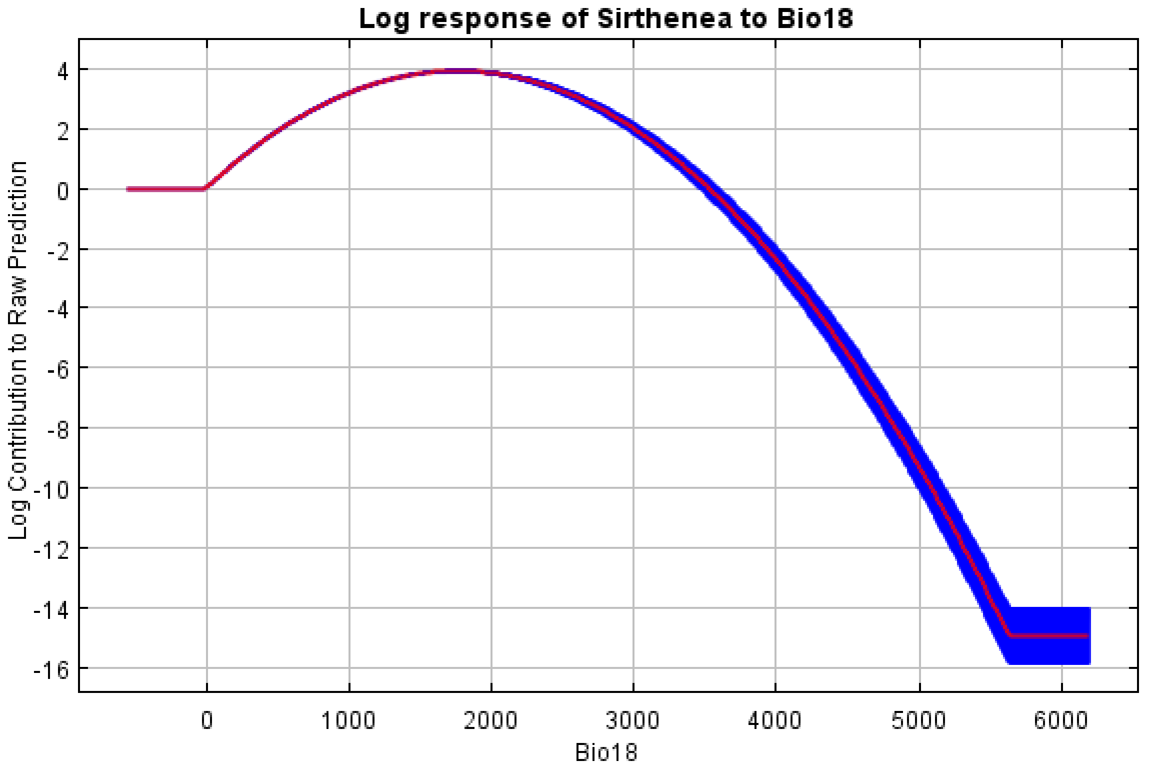

(d) the whole world

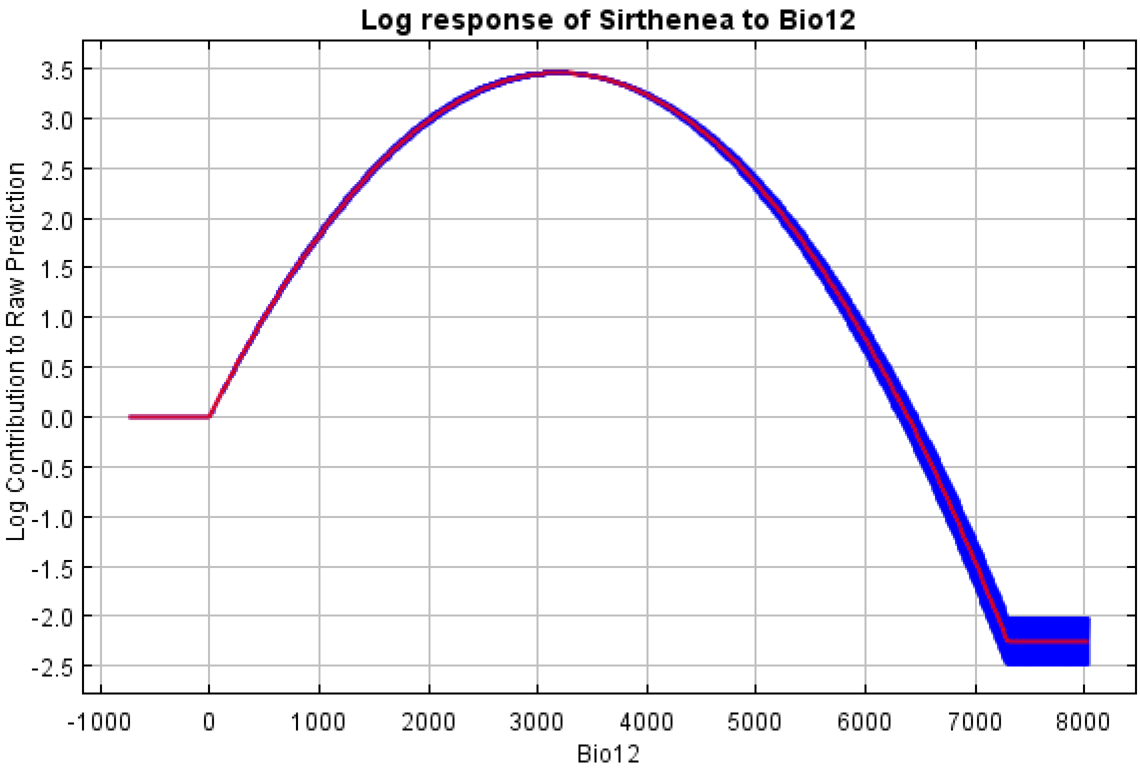

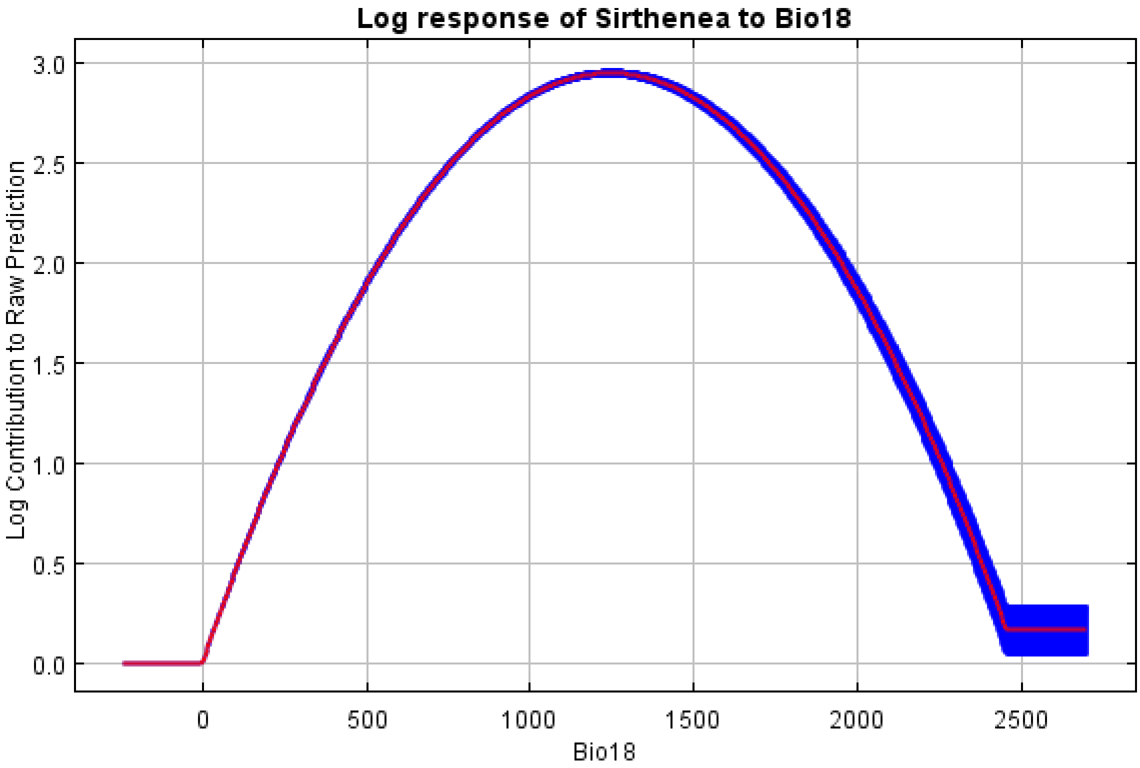

Figure S3.6. Results of response curves for annual precipitation (Bio12) and precipitation of warmest quarter for (a) Africa, (b) Americas, (c) Asia and Australia and (d) the whole world. The curves show

the mean response of the 50 replicate Maxent runs (red) and the mean +/- one standard deviation (blue, two shades for categorical variables).

(a) Americas

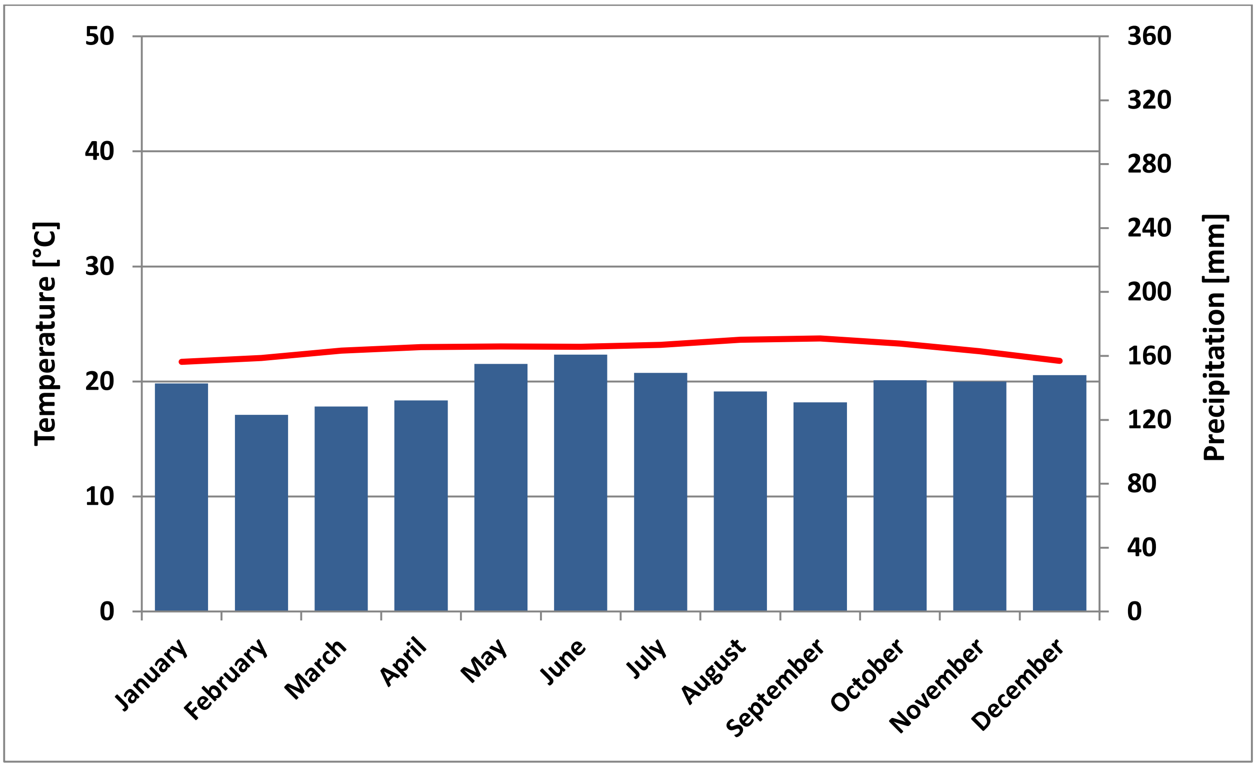

(b) Africa

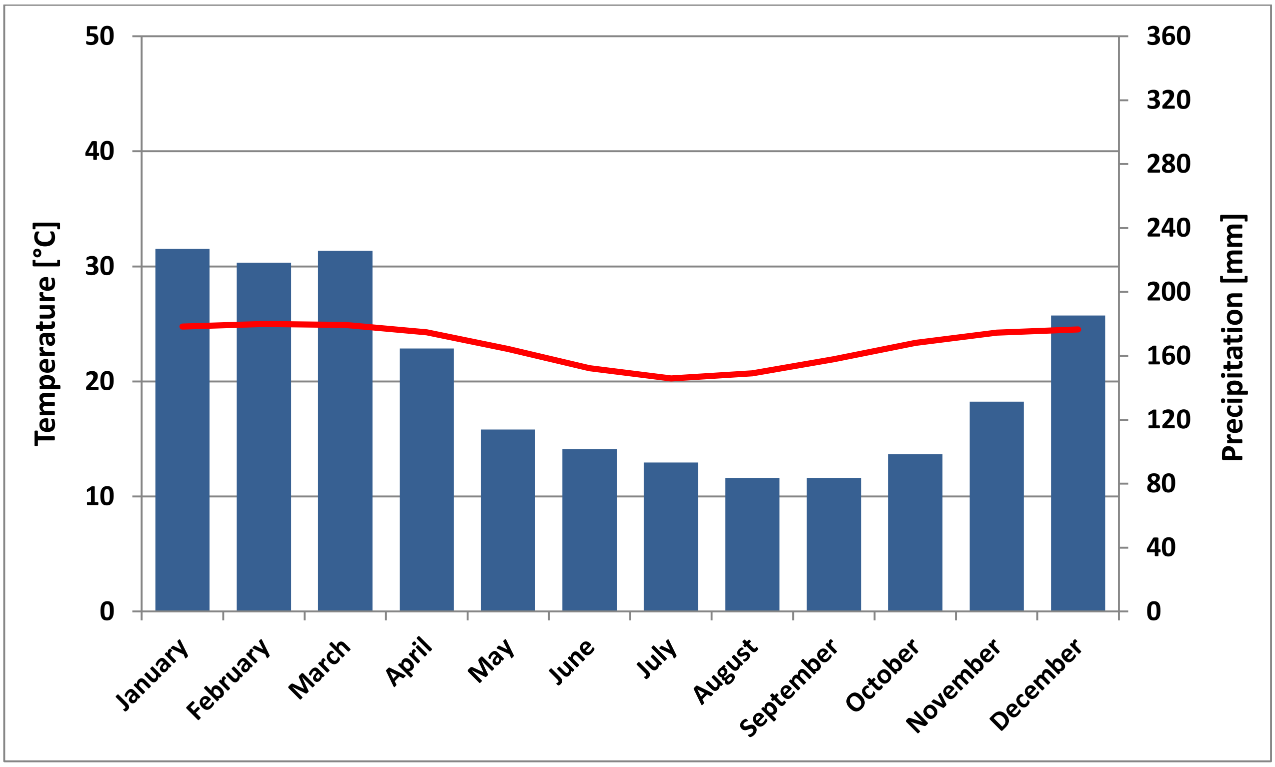

(c) Asia and Australia

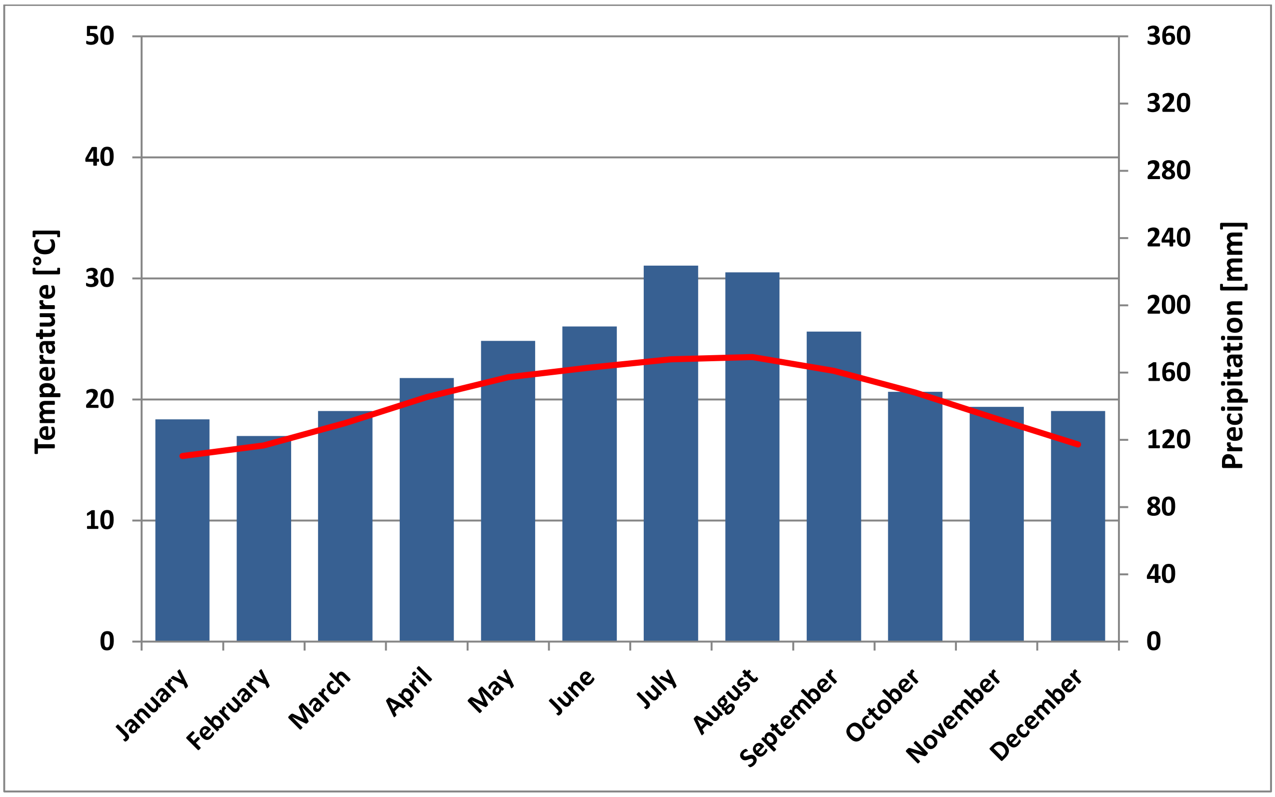

Figure S3.7. The climatic diagrams for (a) Americas, (b) Africa and (c) Asia and Australia. Line shows the temperature distribution during the year and bars represent the distribution of precipitation throughout the year. For each month, the average value from all known locations of the species is given.
