# Supplementary material for: Distribution Pattern and Climate Preferences of the Representatives of the Cosmopolitan Genus Sirthenea Spinola, 1840 (Heteroptera: Reduviidae: Peiratinae)
Source: PLoS One. 2015 Oct 23;10(10):e0140801. doi: 10.1371/journal.pone.0140801 (PMC4619806; doi:10.1371/journal.pone.0140801)
Supplement: S4 Appendix — (PDF) [file pone.0140801.s004.pdf]

Supporting Information

Distribution pattern and climate preferences of the representatives of the genus *Sirthenea* Spinola, 1840 (Heteroptera: Reduviidae: Peiratinae)

PLOS ONE

Dominik Chłond*, Agnieszka Bugaj-Nawrocka

Department of Zoology, Faculty of Biology and Environmental Protection, University of Silesia, Katowice, Poland

* Correspondence: Dominik Chłond, Department of Zoology, Faculty of Biology and Environmental Protection, University of Silesia, Bankowa 9, 40-007 Katowice, Poland.

e-mail: dominik.chlond@us.edu.pl; abugaj-nawrocka@us.edu.pl

Supporting Information S4: Detailed maps of potentially suitable niches for representatives of the genus *Sirthenea*

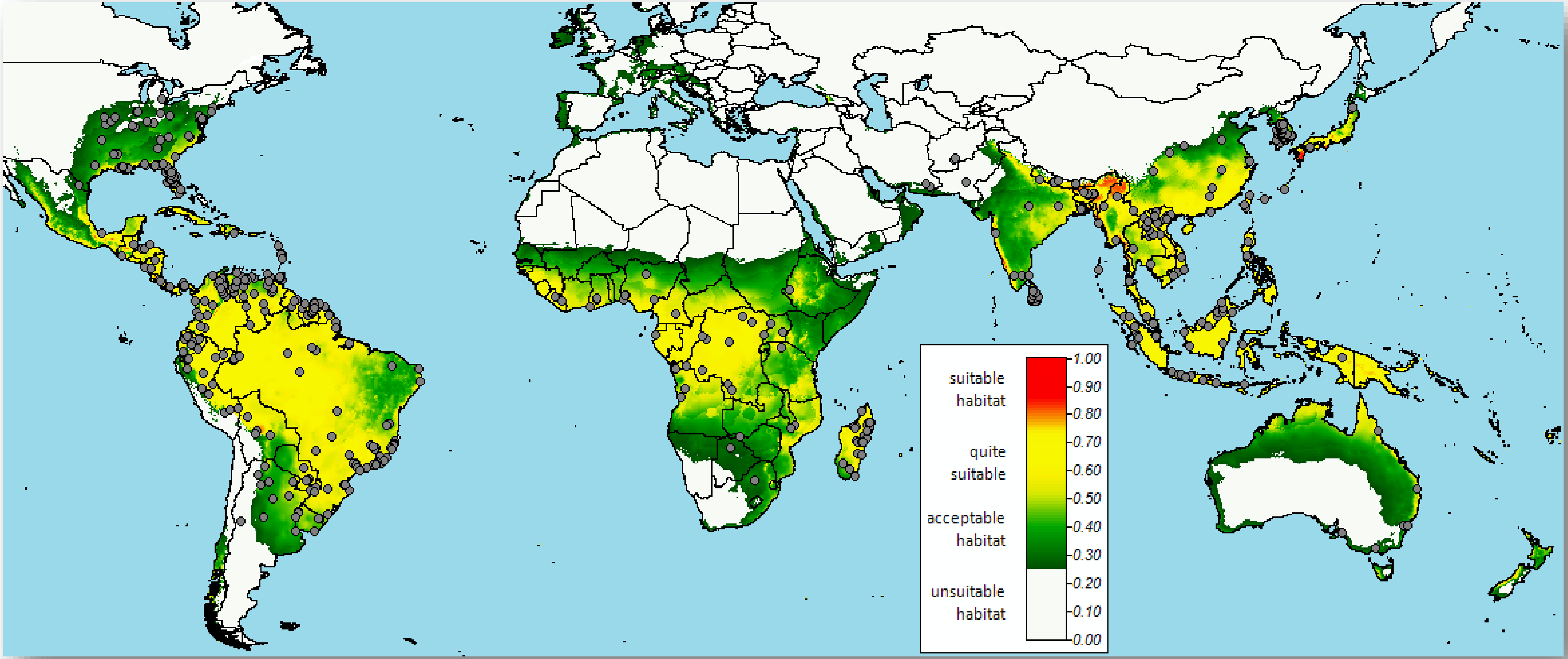

Figure S4.1. Map of the known distribution of representatives of the genus *Sirthenea* against the background of the predicted suitable niches around the world.

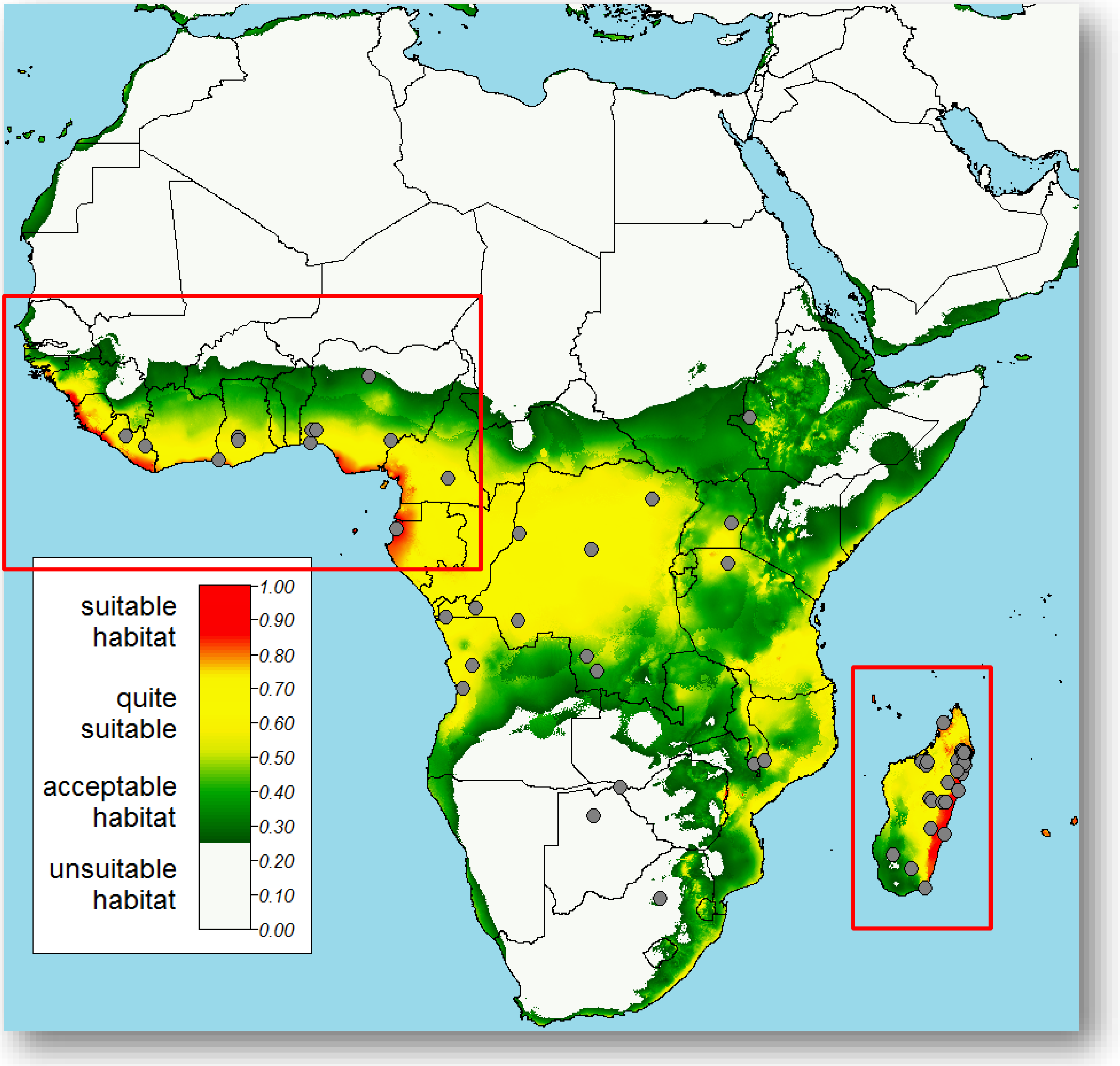

Figure S4.2. Predicted probability of presence of suitable niches for the representatives of the genus *Sirthenea* in Africa according to all selected variables. Inland continuous lines represent country boundaries. Red borders indicate the areas which are shown below in details.

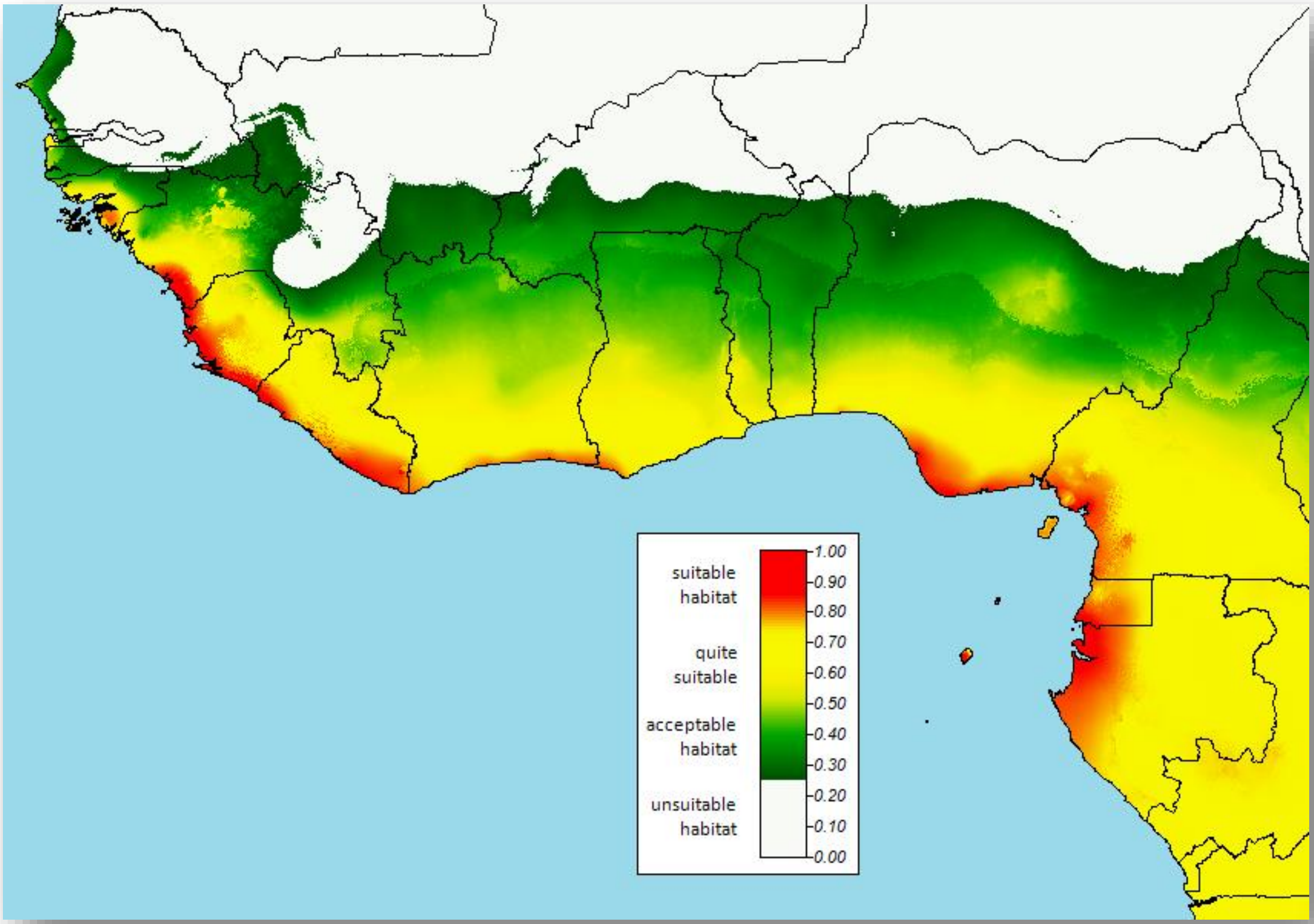

Figure S4.3. Predicted probability of presence of suitable niches for the representatives of the genus *Sirthenea* according to all selected variables. Map shows the western part of Africa, near the Gulf of Guinea.

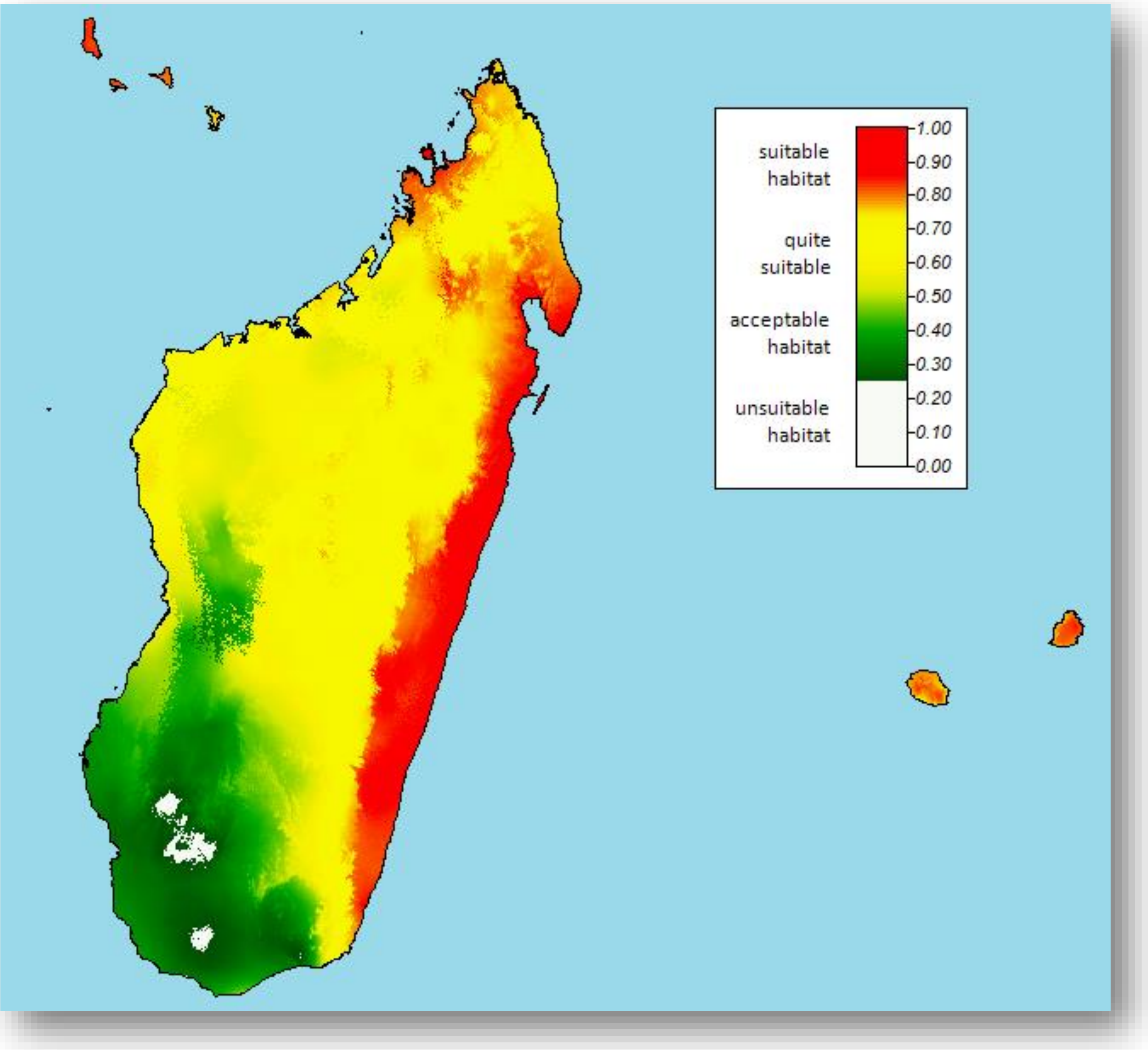

Figure S4.4. Predicted probability of presence of suitable niches for the representatives of the genus *Sirthenea* according to all selected variables. Map shows Madagascar.

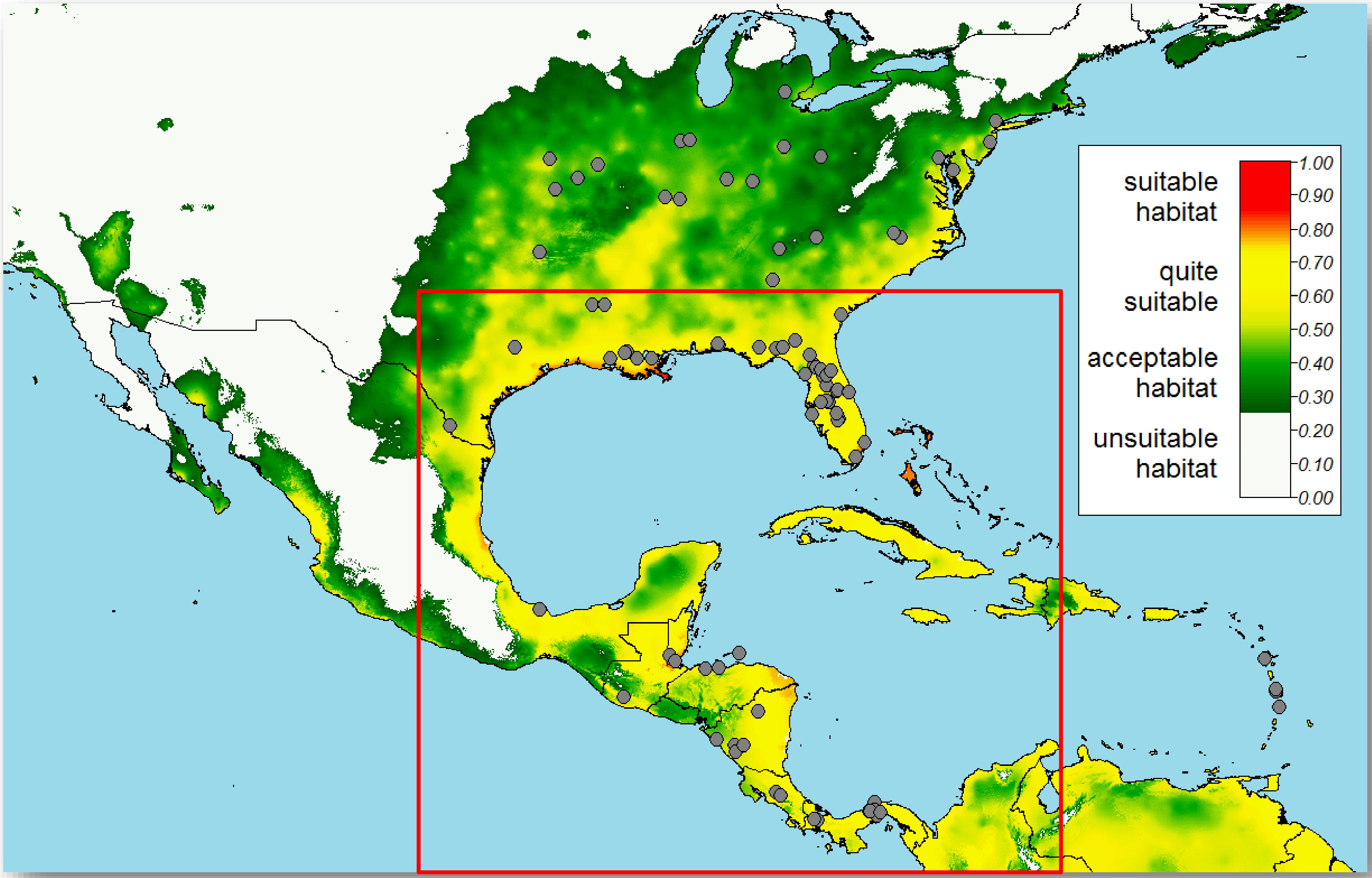

Figure S4.5. Predicted probability of presence of suitable niches for the representatives of the genus *Sirthenea* in North America according to all selected variables. Inland continuous lines represent country boundaries. Red borders indicate the areas which are shown below in details.

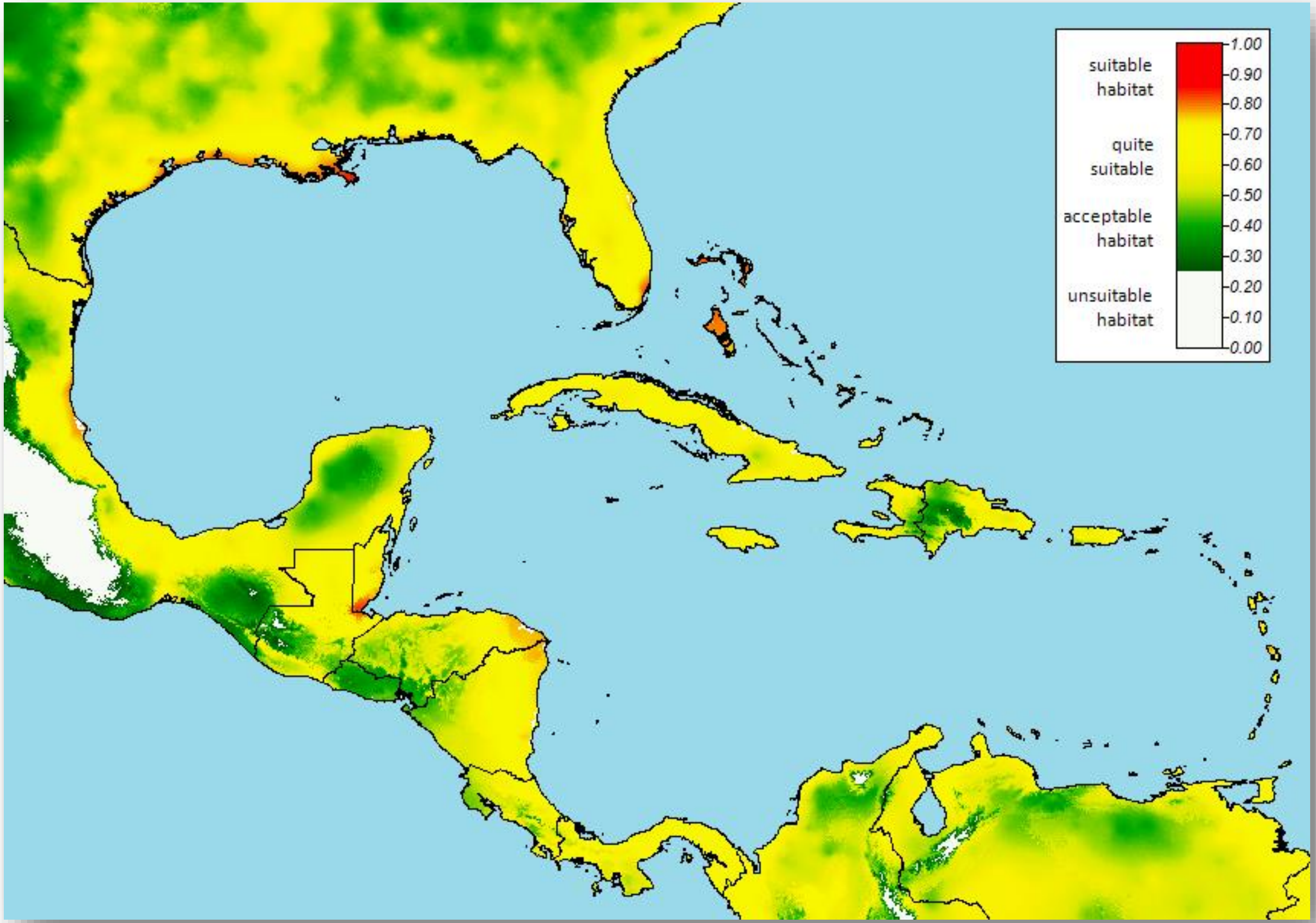

Figure S4.6. Predicted probability of presence of suitable niches for the representatives of the genus *Sirthenea* according to all selected variables. Map shows the areas around the Gulf of Mexico and Caribbean Sea.

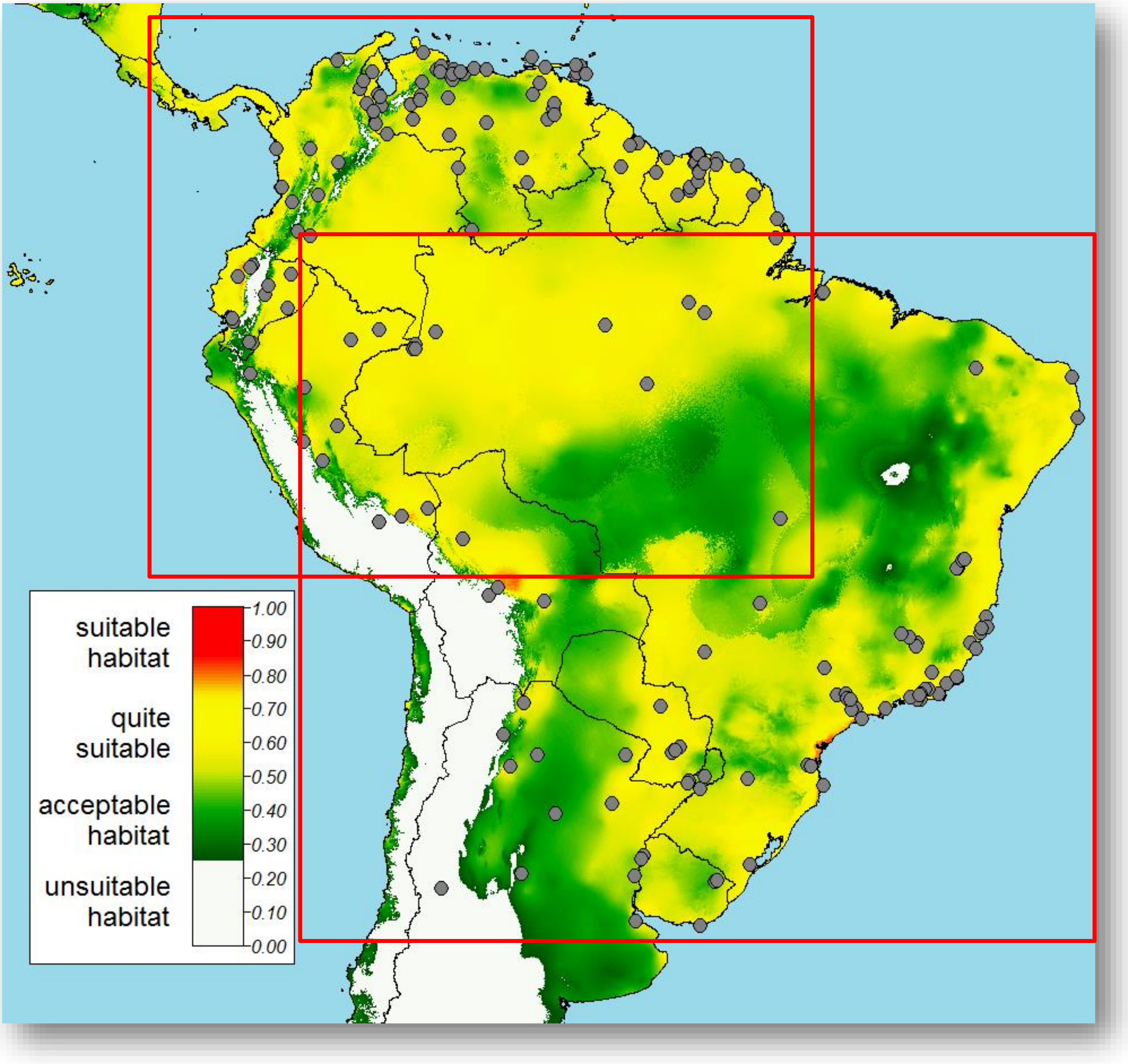

Figure S4.7. Predicted probability of presence of suitable niches for the representatives of the genus *Sirthenea* in South America according to all selected variables. Inland continuous lines represent country boundaries. Red borders indicate the areas which are shown below in details.

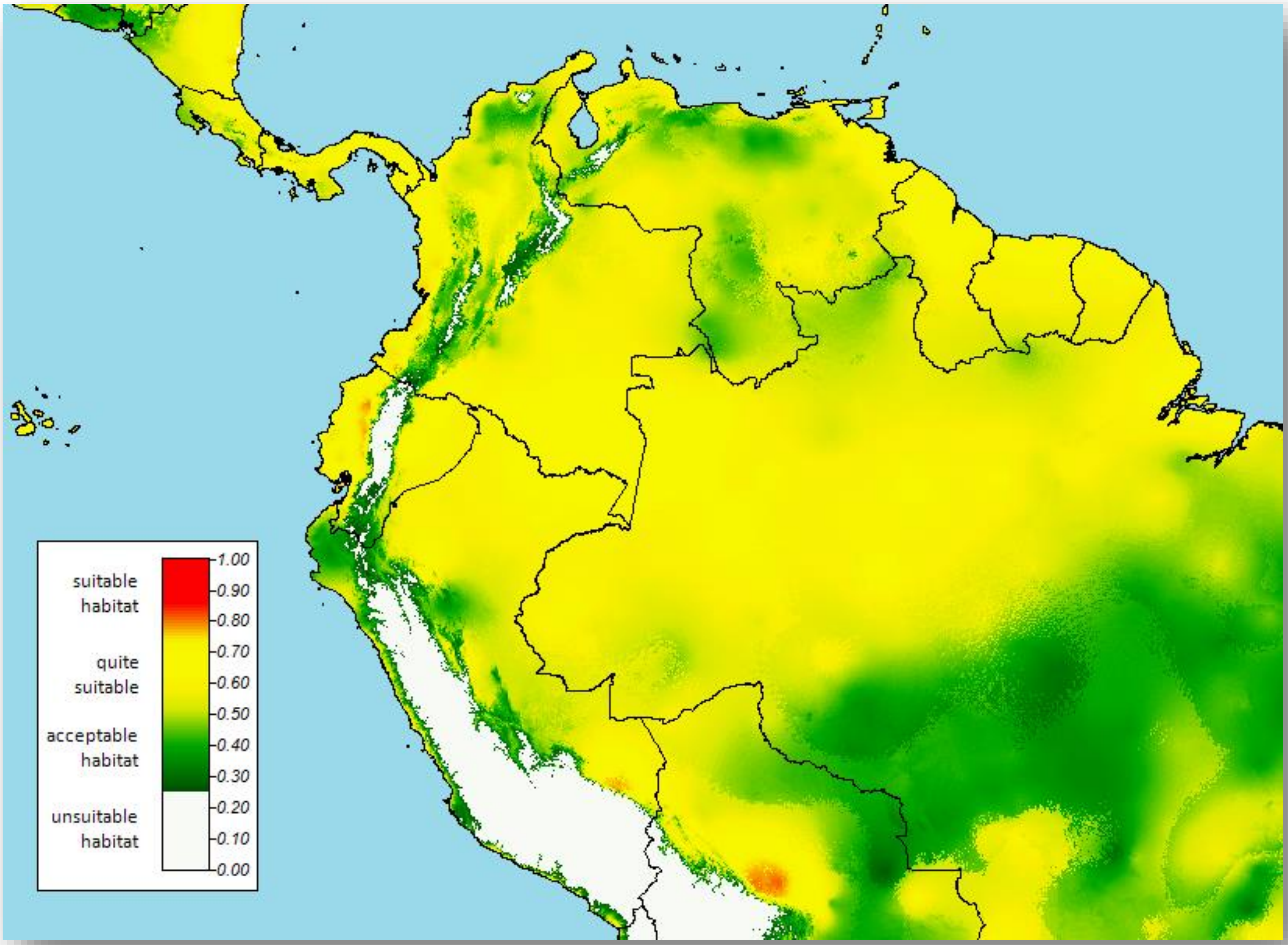

Figure S4.8. Predicted probability of presence of suitable niches for the representatives of the genus *Sirthenea* according to all selected variables. Map shows the north-western part of the continent with the Orinoco Basin, Guiana Shield and the Amazon Basin.

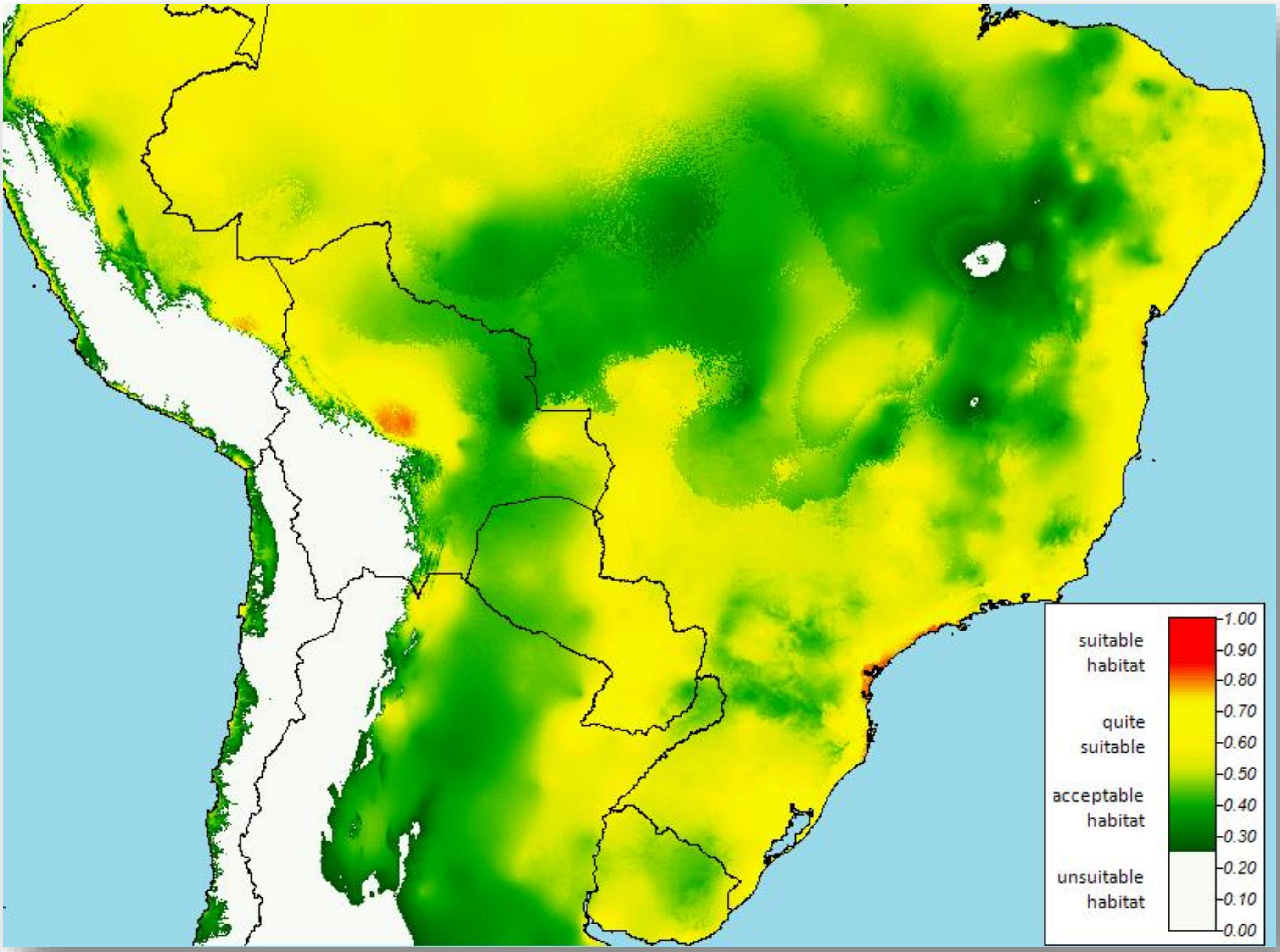

Figure S4.9. Predicted probability of presence of suitable niches for the representatives of the genus *Sirthenea* according to all selected variables. Map shows the central part of the continent with the Río de la Plata Basin and the Brazilian Highlands.

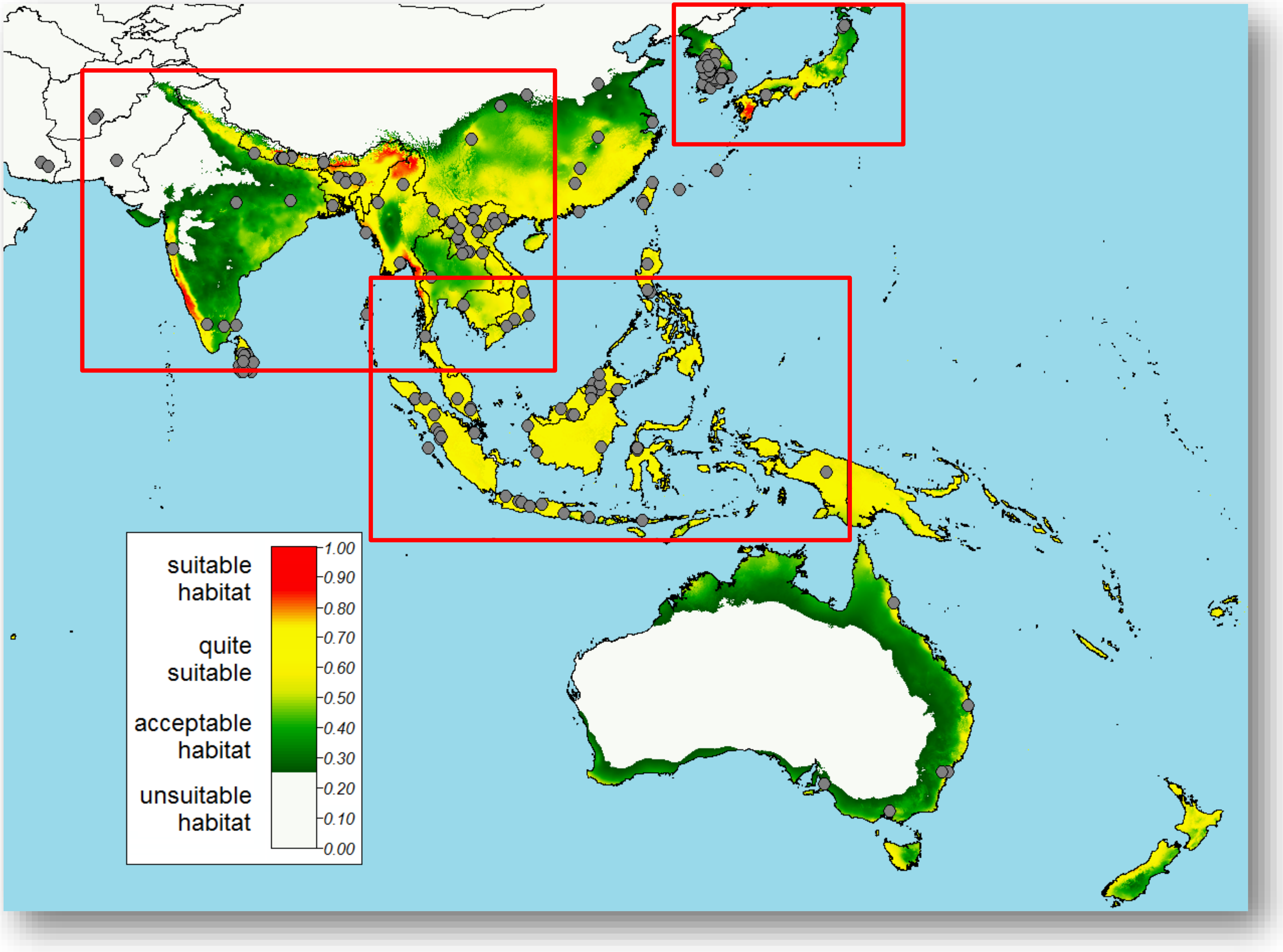

Figure S4.10. Predicted probability of presence of suitable niches for the representatives of the genus *Sirthenea* in Asia, Australia and Oceania according to all selected variables. Inland continuous lines represent country boundaries. Red borders indicate the areas which are shown below in details.

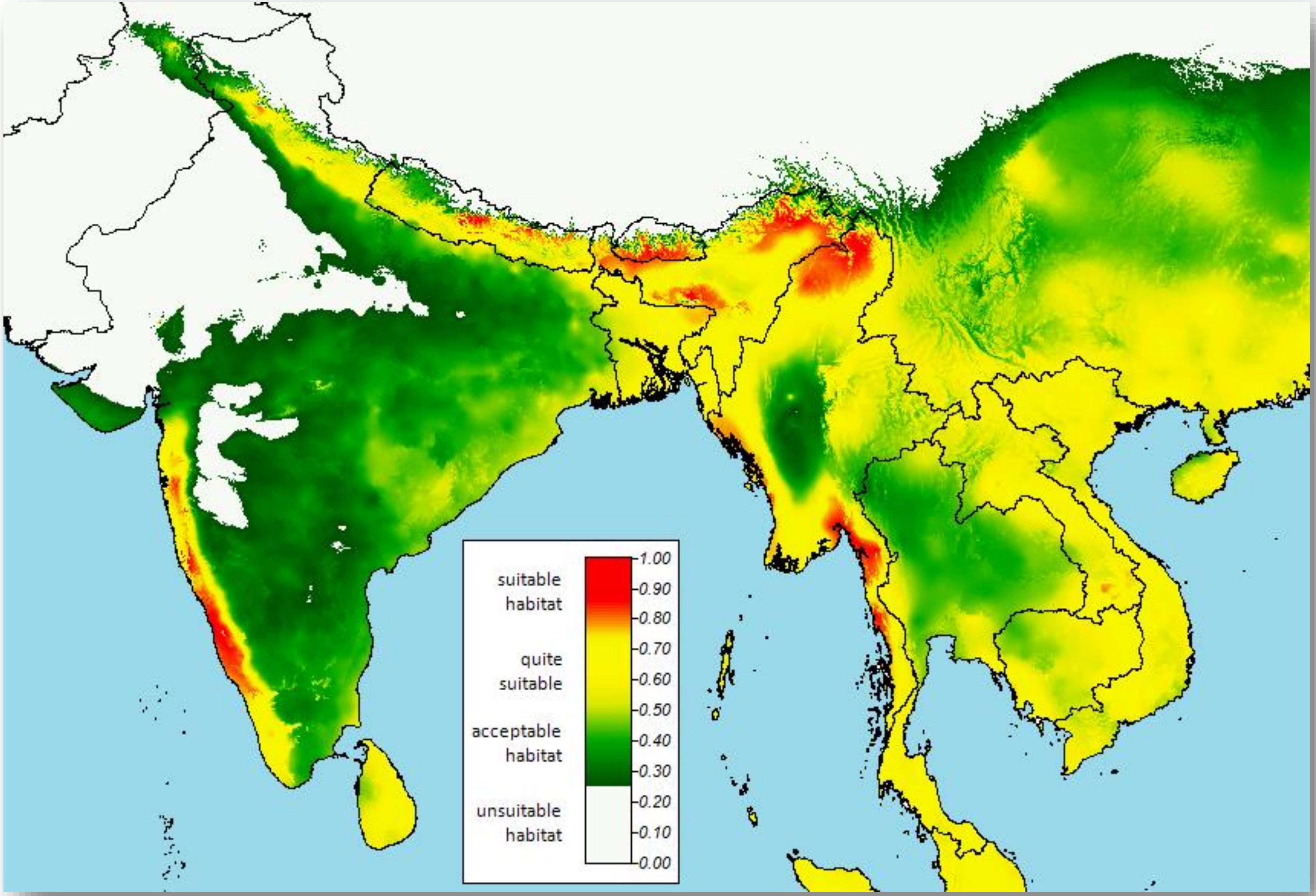

Figure S4.11. Predicted probability of presence of suitable niches for the representatives of the genus *Sirthenea* according to all selected variables. Map shows the Indian subcontinent and the mainland Southeast Asia (Indochina).

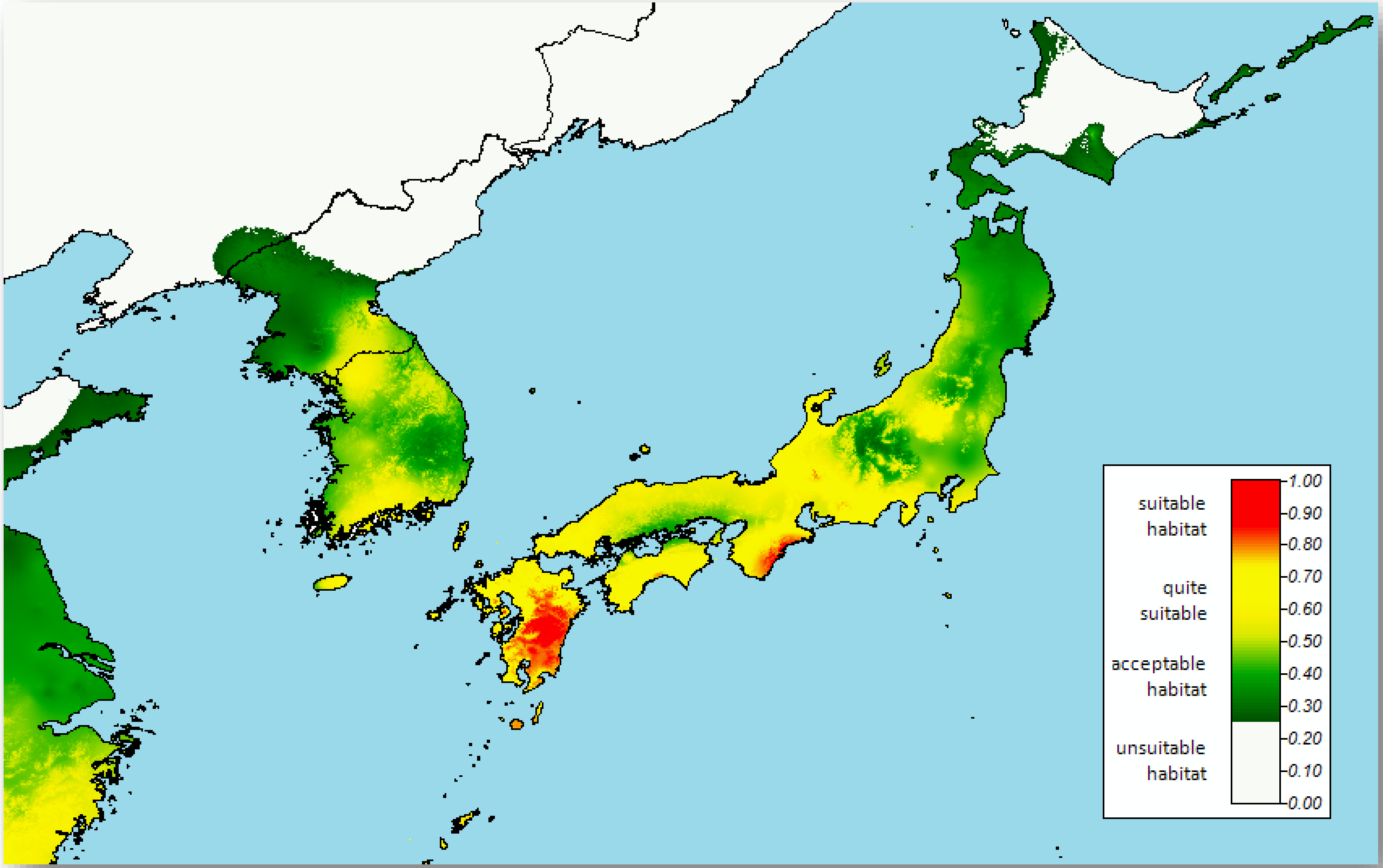

Figure S4.12. Predicted probability of presence of suitable niches for the representatives of the genus *Sirthenea* according to all selected variables. Map shows the Korean Peninsula and the Japanese archipelago.

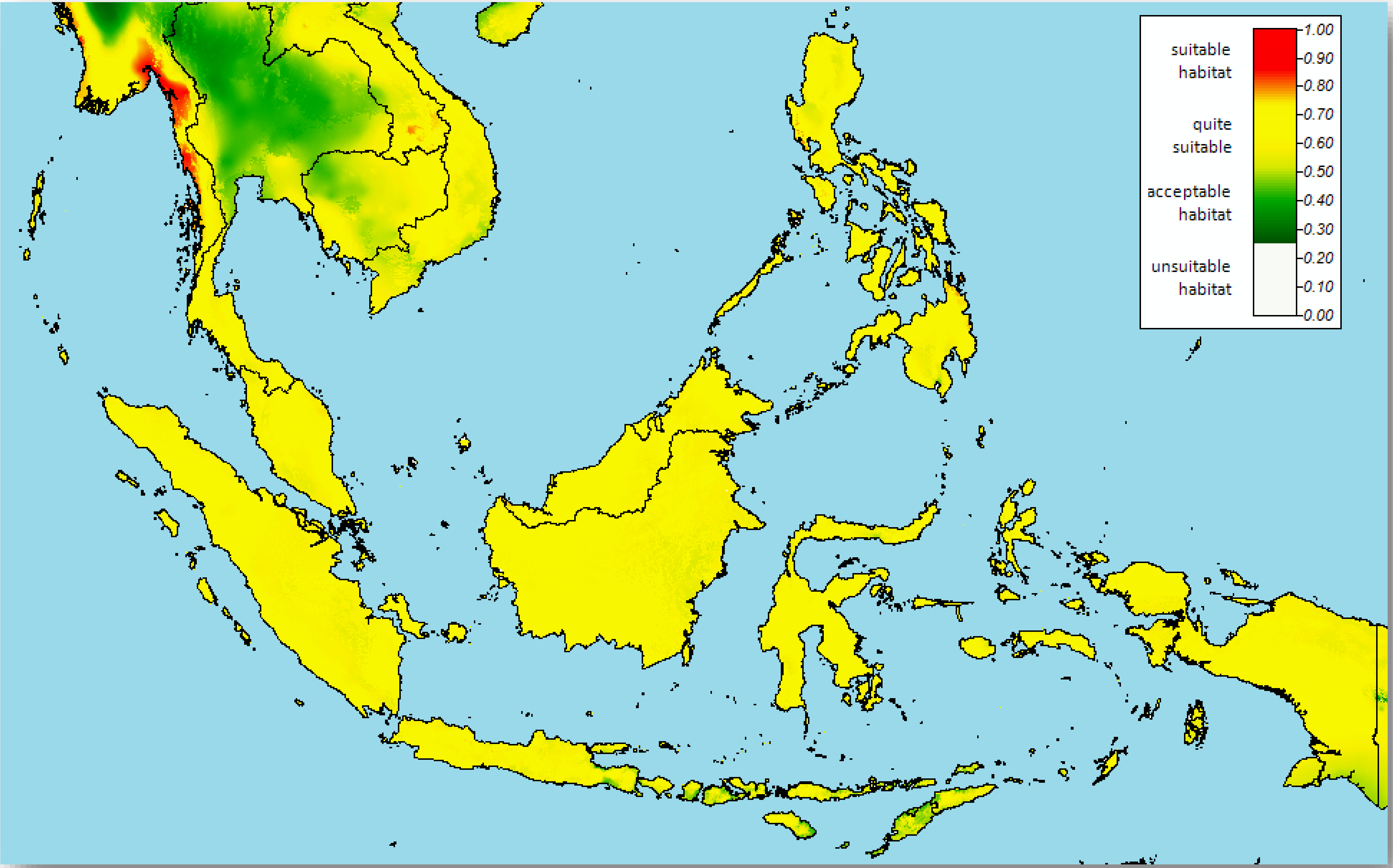

Figure S4.13. Predicted probability of presence of suitable niches for the representatives of the genus *Sirthenea* according to all selected variables. Map shows the Malay Archipelago.
